# Stefano Marcaccini: a pioneer in isocyanide chemistry

**DOI:** 10.1007/s11030-023-10641-7

**Published:** 2023-04-12

**Authors:** Ana G. Neo, José Luis Ramiro, María García-Valverde, Jesús Díaz, Carlos F. Marcos

**Affiliations:** 1 Laboratory of Bioorganic Chemistry & Membrane Biophysics (L.O.B.O.), Universidad de Extremadura, 10003 Cáceres, Spain; 2 Departamento de Química, Facultad de Ciencias, Universidad de Burgos, 09001 Burgos, Spain

**Keywords:** Isocyanides, Multicomponent reactions, Heterocycles, Organic synthesis, Cycloadditions

## Abstract

Stefano Marcaccini was one of the pioneers in the use of isocyanide-based multicomponent reactions in organic synthesis. Throughout his career at the University of Florence he explored many different faces of isocyanide chemistry, especially those geared towards the synthesis of biologically relevant heterocycles. His work inspired many researchers who contributed to other important developments in the field of multicomponent reactions and created a school of synthetic chemists that continues today. In this manuscript we intend to review the articles on isocyanide multicomponent reactions published by Dr. Marcaccini and analyse their influence on the following works by other researchers. With this, we hope to highlight the immense contribution of Stefano Marcaccini to the development of isocyanide chemistry and modern organic synthesis as well as the influence of his research on future generations. We believe that this review will not only be a well-deserved tribute to the figure of Stefano Marcaccini, but will also serve as a useful inspiration for chemists working in this field.

**Graphical abstract:**

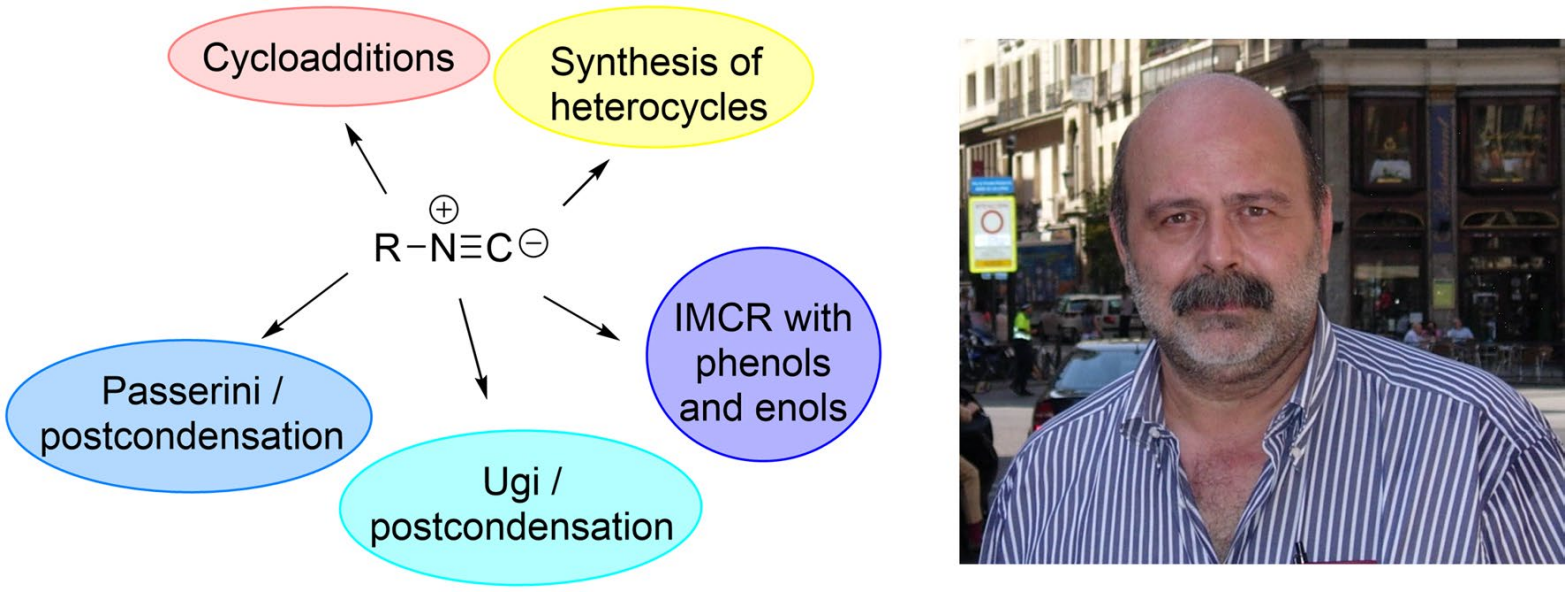

## Introduction

Stefano Marcaccini was born in Prato (Florence) on the 31 of January of 1956. He studied at the University of Florence, where he got a PhD in Organic Chemistry in 1982 under the supervision of Professor Valerio Parrini. Between 1994 and 1995, he held a position as associate professor of Heterocyclic Chemistry at the University of Siena. He then returned to the University of Florence, where he spent most of his career, conducting outstanding research on the synthesis of heterocycles that led to novel methods based primarily on isocyanide reactions.

He became a world recognised expert on the chemistry of isocyanides, having published a hundred of research papers on this field. His contribution has an unquestionable impact on the current state of the art of multicomponent reactions. He mentored many students, and he was appreciated, not only for his quality as educator and his extraordinary chemical intuition, but especially for his exceptional generosity and kindness. His work inspired many researchers and sparked the creation of a school of synthetic chemists that is still very active today.

Stefano Marcaccini died at Prato on the 1st of October 2012. He is remembered as a remarkably gifted chemist and an excellent person.
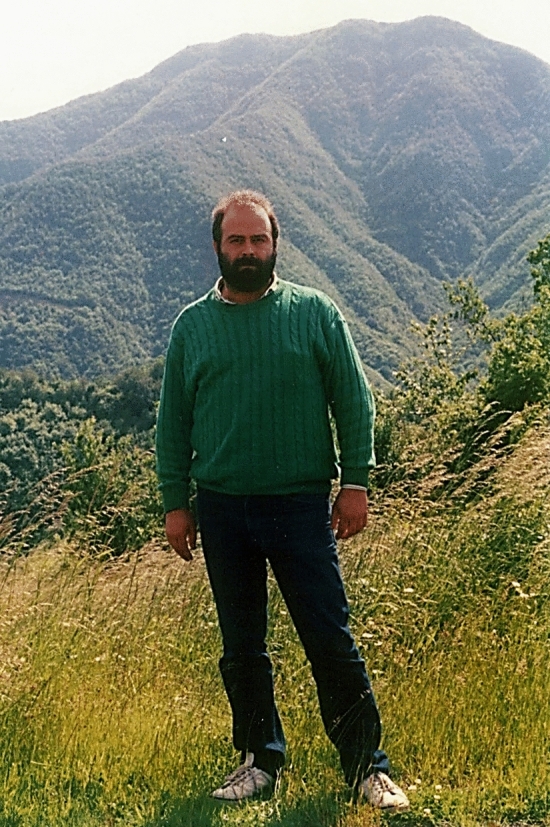


## Synthesis of heterocycles from bifunctional isocyanides

### Isocyanoacetate derivatives

In the mid-80s, Marcaccini’s work was focused on heterocyclic chemistry. Thus, with the aim of developing novel ways to achieve heterocyclic scaffolds he turned the spotlight into isocyanide chemistry, specifically isocyanoacetate derivatives (**1**). He reasoned that the acidic position α to the isocyanide could be used to cyclise onto suitable functional groups introduced with other reagents. He was probably inspired by the seminal research of Schöllkopf on the coupling of isocyanoacetate (**1**) with acyl chlorides (**6**) [1, 2], and of Van Leusen, on *p*-toluenesulfonylmethyl isocyanide (TOSMIC) chemistry [3].

**Scheme 1:**
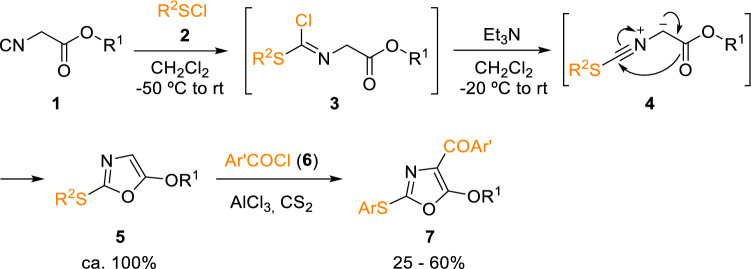
Synthesis of substituted oxazoles

Thus, he reacted 2-isocyanoacetate (**1**) with sulphur electrophiles, such as sulfenyl chlorides (**2**), generating a reactive intermediate (**3**), which would undergo an intramolecular cyclisation in the presence of a base. He and his co-workers synthesised different heterocyclic systems using this strategy. For example, in his first work with isocyanides, 2-arylthio-5-alkoxyoxazoles (**5**) were constructed in almost quantitative yields in a one pot process (Scheme 1) [4]. A few years later, Marcaccini and Torroba expanded this study by acylating oxazole (**5**) to afford trisubstituted oxazoles (**7**; Scheme 1) [5]. Most likely, these reactions proceed through nitrilium ylide intermediates (**4**), which have ever since been revealed as a common and very useful feature of the chemistry of isocyanoacetate derivatives offering a broad variety of a common and very useful feature applications in the multicomponent synthesis of heterocycles [6].

**Scheme 2:**
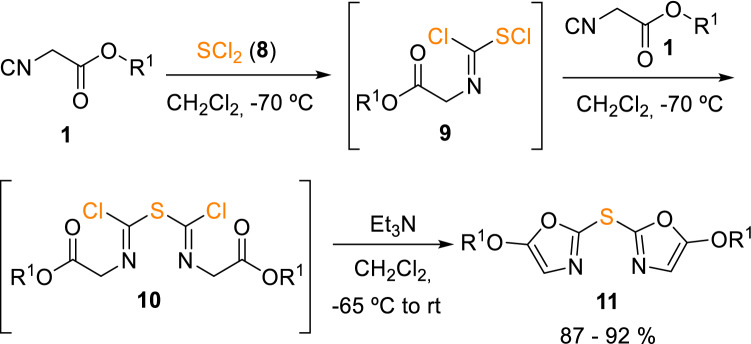
Synthesis of 5,5′-dialkoxy-2,2′-dioxazolylsulfides

Similarly, dioxazolylsulfides (**11**) were prepared from alkyl isocyanoacetates (**1**) and sulphur dichloride (**8**). A 2:1 molar ratio was used in this case to favour the formation of a labile intermediate (**10**), which easily cyclised in presence of a base leading to bisoxazolyl sulfane (**11**) in high yields (Scheme 2) [7].

**Scheme 3:**
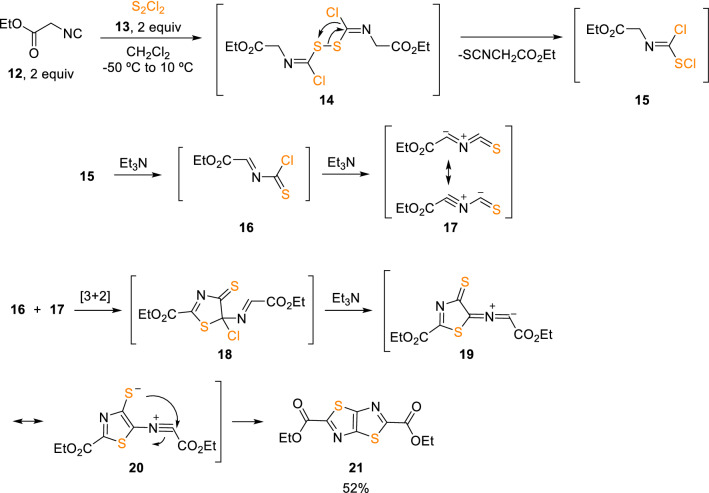
Synthesis of thiazolo[5,4-*d*]-thiazoles

Additionally, the use of alkyl isocyanoacetates (**12**) and S_2_Cl_2_ (**13**) unexpectedly resulted in a convenient synthesis of fused heterocyclic cores, such as thiazolo[5,4-*d*]-thiazoles (**21**; Scheme 3) [8]. This molecular system has been recognised as an important motif for molecular optic-electronic [9] and photovoltaic applications [10]. The mechanism of the reaction appears to involve a dimerisation of intermediate (**16**), which was confirmed as the same thiazolo [5,4-*d*]thiazole (**21**) that can be obtained by reacting SCl_2_ (**8**) with isocyanoacetate (**12**) in a 1:1 molar ratio.

**Scheme 4:**
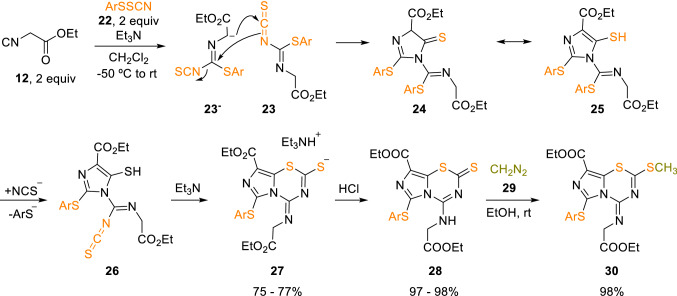
Synthesis of 6-arylthio-8-ethoxycarbonyl-4-ethoxycarbonylmethylaminoimidazo[5,1-*b*][1,3,5]thiadiazine-2-thiones

Other complex heterocyclic systems, such as 6-arylthio-8-ethoxycarbonyl-4-ethoxycarbonylmethylaminoimidazo[5,1-*b*][1,3,5]thiadiazine-2-thiones (**28**) could be obtained from alkyl isocyanoacetates (**12**) and arylsulfenyl thiocyanates (**22**). The intermediate salt (**27**) was treated with acid to afford thione (**28**), which could be further methylated to give the corresponding SCH_3_ derivatives (**30**; Scheme 4) [11].

**Scheme 5:**
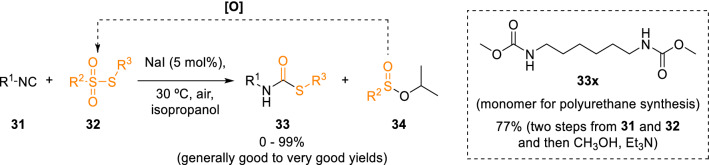
Secondary thiocarbamate synthesis from isocyanides

Inspired by the general idea of Marcaccini’s seminal works, different strategies have recently been developed to elicit the coupling of isocyanides with sulphur electrophiles. For example, Mampuys et al. synthesised secondary thiocarbamates (**33**) from isocyanides (**31**) and thiosulfonates (**32**) in a transition metal-free protocol enabled by the catalytic electron transfer role played by inexpensive NaI (Scheme 5) [12].

**Scheme 6:**
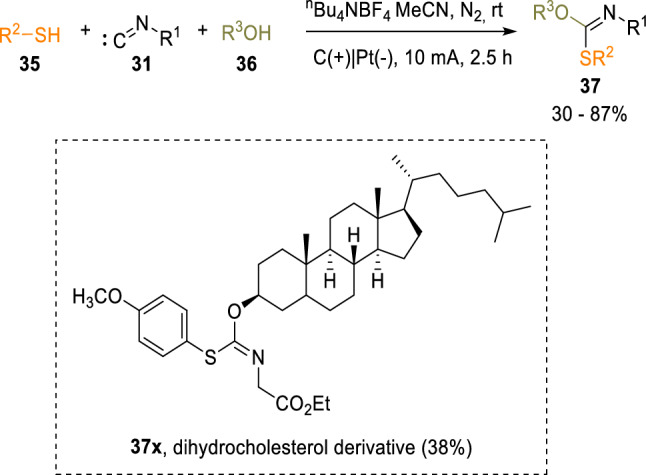
Electrochemical synthesis of imino-sulphide ethers

Using a novel perspective, Guan’s group employed an organic electrochemistry approach for the activation of isocyanides (**31**) with sulphur electrophiles. In this approach, the reaction of isocyanides (**31**) and thiols (**35**) afforded a wide range of imino sulphide ethers (**37**), which are important as pharmaceuticals and as key intermediates in sugar chemistry (Scheme 6) [13].

**Scheme 7:**
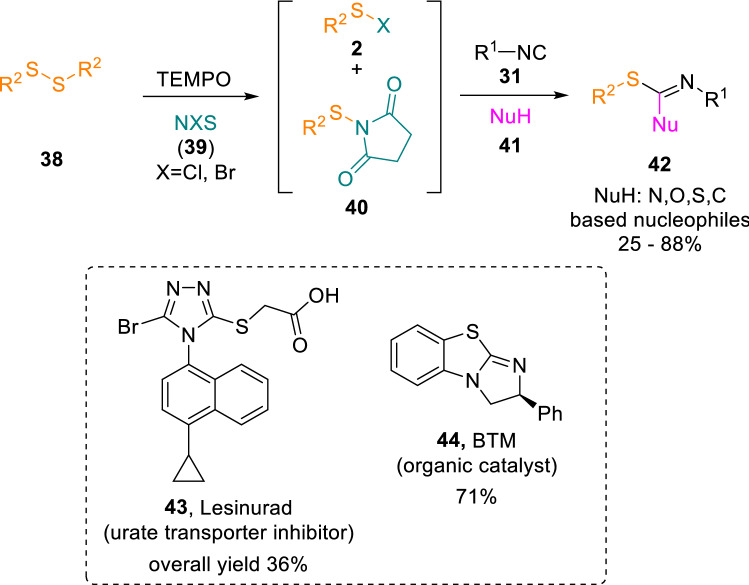
Activated disulphides in reactions with isocyanides and nucleophiles

An interesting related approach has been reported by Sun and co-workers, who reacted disulphides (**38**) with isocyanides (**31**) in the presence of TEMPO and *N*-halosuccinimides (**39**) to give insertion products that can further incorporate a wide range of nucleophiles (**41**) [14]. This sequence led to the multicomponent construction of different molecular scaffolds (**42**) in mild conditions (Scheme 7).

**Scheme 8:**
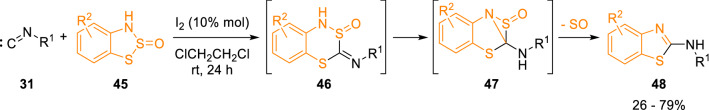
Marcos’ synthesis of 2-amino-benzothiazoles

Isocyanide insertion into S–S bonds can be also used in the construction of heterocycles. Thus, Marcos et al. developed a straightforward method for the synthesis of 2-amino-benzothiazoles (**48**) through an iodine-catalysed insertion of isocyanides into the S–S bond of benzodithiazole 2-oxides (**45**), with concomitant extrusion of sulphur monoxide (Scheme 8) [15].

**Scheme 9:**
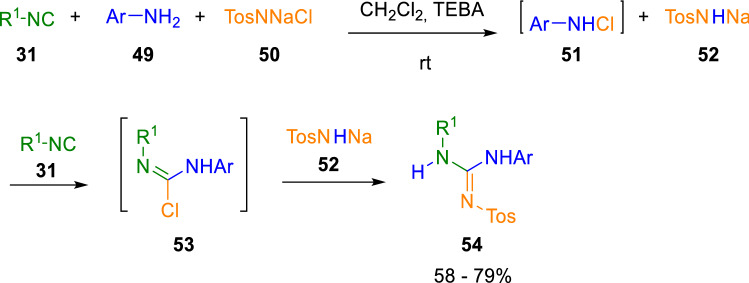
Synthesis of sulfonylguanidines

The insertion of isocyanides into N–Cl bonds is also possible. Marcaccini´s group exploited the chemistry of isocyanides to synthesise different unusual molecules not easily attainable by other procedures, highlighting the great potential of this functional group [16]. For example, they easily obtained sulfonylguanidines (**54**) through the reaction of isocyanides (**31**) with anilines (**49**) and chloramine T (**50**) under phase transfer-catalysed conditions at room temperature (Scheme 9) [17].

The authors argue that the formation of *N*-chloroamines (**51**) is the crucial step in this synthesis. Specifically, they propose that the reaction takes place by the *N*-chlorination of aromatic amines (**49**) to give *N*-chloroanilines (**51**)**,** which react with isocyanides (**31**) to give the α-adducts (**53**). These then react with sodium tosylamide (**52**) to form the sulfonylguanidines (**54**).

**Scheme 10:**
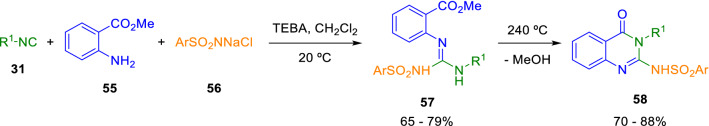
Synthesis of quinazoline derivatives

The use of bifunctional amines may facilitate post-condensation transformations leading to heterocyclic products. Therefore, when the reaction was carried out with methyl anthranilate (**55**) the resulting intermediate sulfonylguanidine (**57**) underwent a heating mediated cyclisation to readily give quinazoline derivatives (**58**; Scheme 10) [18].

### Isocyanoacetamide derivatives

Isocyanoacetamides (**59**), the corresponding amide derivatives of alkyl isocyanoacetates (**1**), are also powerful reagents for heterocyclic synthesis with exceptional characteristics [19].

**Scheme 11:**
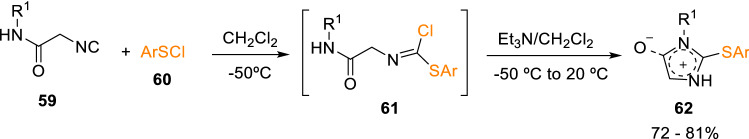
Synthesis of mesoionic 3-alkyl-2-arylthio-1,3-diazolium-4-olates from isocyanamides

Marcaccini et al., in parallel with their research on 2-isocyanoacetates (**1**), pioneered the reaction between isocyanoamides (**59**) and sulphur chloride derivatives, such as aryl sulfenyl chlorides (**60**), to afford mesoionic heterocycles (**62**; Scheme 11) [20]. As in similar reactions with isocyanoacetates (**1**), the product is obtained by a base mediated cyclisation of the α-addition intermediate (**61**).

**Scheme 12:**
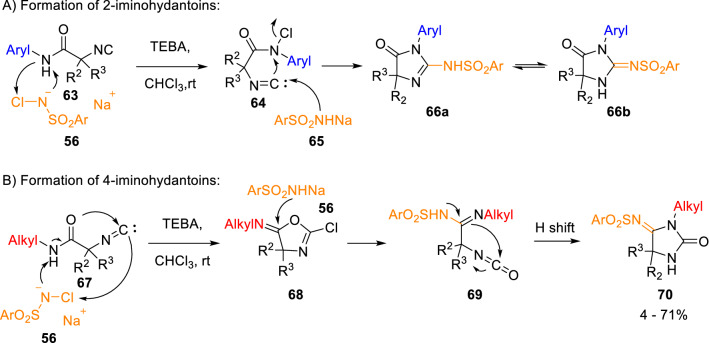
Regioselective synthesis of iminohydantoins

Following this, García-Valverde and Marcaccini developed a novel regioselective and experimentally simple synthesis of iminohydantoins from isocyanoacetamides (**63**) and chloroamines (**56**). 2-Iminohydantoins (**66**) and 4-iminohydantoins (**70**) were selectively obtained starting from *N*-aryl (**63**) or *N*-alkylisocyanoacetamides (**67**), respectively (Scheme 12) [21]. According to the mechanism proposed, regioselectivity is controlled by the electron density of the amide nitrogen. Aryl substituents generate lower electron density at the amide nitrogen, which favours a faster chlorination rection leading to (**64**) and, finally, to 2-iminohydantoins (**66**). Conversely, alkyl groups generate a higher electron density at the amide group, which causes slower rates of chlorination, and allow a competitive reaction of α-addition to the isocyanide group to give intermediate 2-chlorooxazolines (**68**), which undergo a ring-opening step and a rearrangement leading to 4-iminohydantoins (**70**). This work constitutes the first example of a regioselective cyclisation controlled by the nature of the substituent on the amide group [21].

**Scheme 13:**
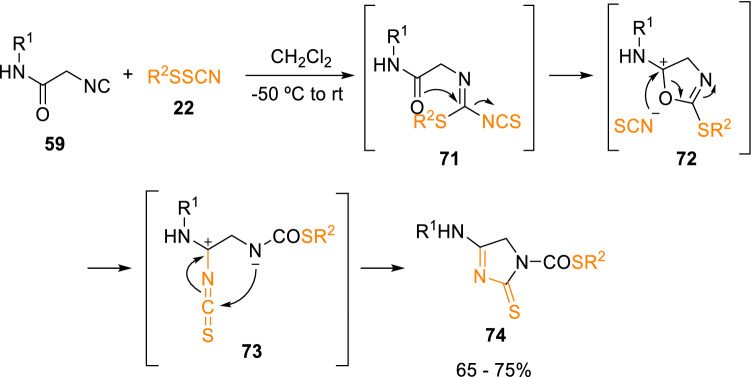
Synthesis of imidazole-2-thiones

Likewise, the coupling of isocyanoacetamides (**59**) and arylsulfenyl thiocyanates (**22**) led to imidazole-2-thiones (**74**) without the need of an additional base (Scheme 13) [22].

**Scheme 14:**
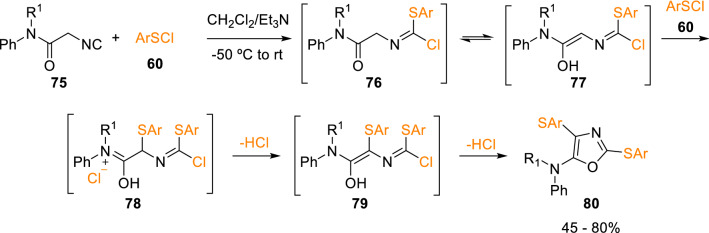
Trisubstituted oxazole synthesis by means of isocyanoacetamides

Interestingly, Marcaccini et al. also found that, in contrast to secondary isocyanoacetamides (**59**), *N,N*-disubstituted isocyanoacetamides (**75**) react with aryl sulfenyl chlorides (**60**) to give oxazoles (**80**). In this case, the enolisation of intermediate (**76**) allows the addition of a second aryl sulfenyl molecule (**60**) prior to cyclisation of the enolic tautomer (**79**; Scheme 14) [23].

**Scheme 15:**
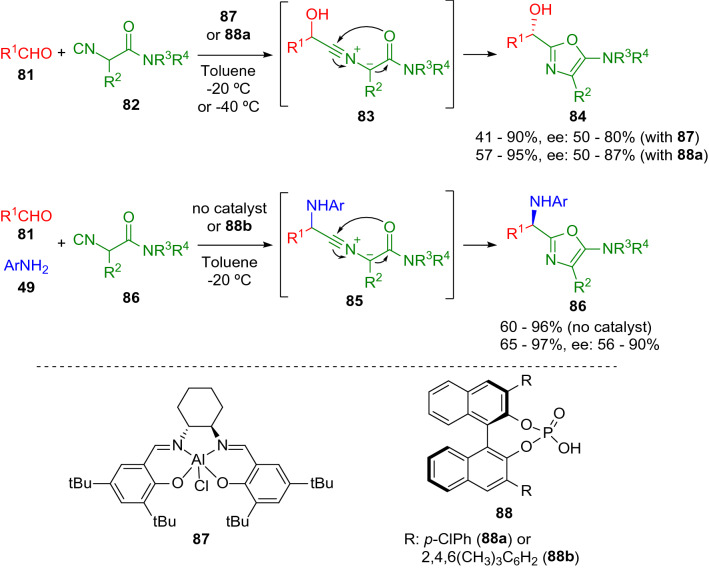
Zhu's oxazole synthesis from isocyanoacetamides

**Scheme 16:**
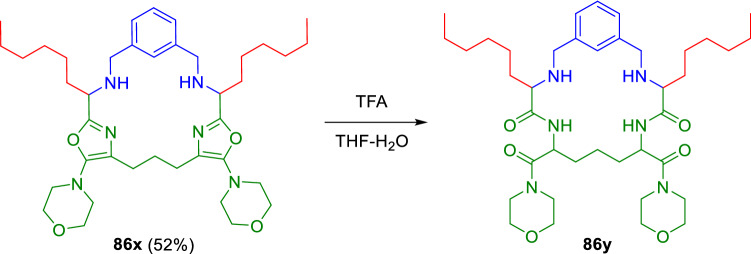
Cyclophanes synthesised from aldehydes, double amines and double isocyanides

Zhu and collaborators made a remarkable contribution with the development of novel two- and three-component oxazole synthesis [19, 24] involving aldehydes (**81**), isocyanoacetamides (**82**) and amines (**49**; Scheme 15). The same methodology was followed to make oxazole-derived cyclophanes with high atom economy in a one-step procedure (Scheme 16) [25]. Moreover, Zhu introduced a related enantioselective methodology using Lewis acid catalysis with a chiral (salen)Al(III)Cl complex (**87**; Scheme 15), [26] or BINOL-derived organophosphoric acids (**88**; Scheme 15) [27, 28].

**Scheme 17:**
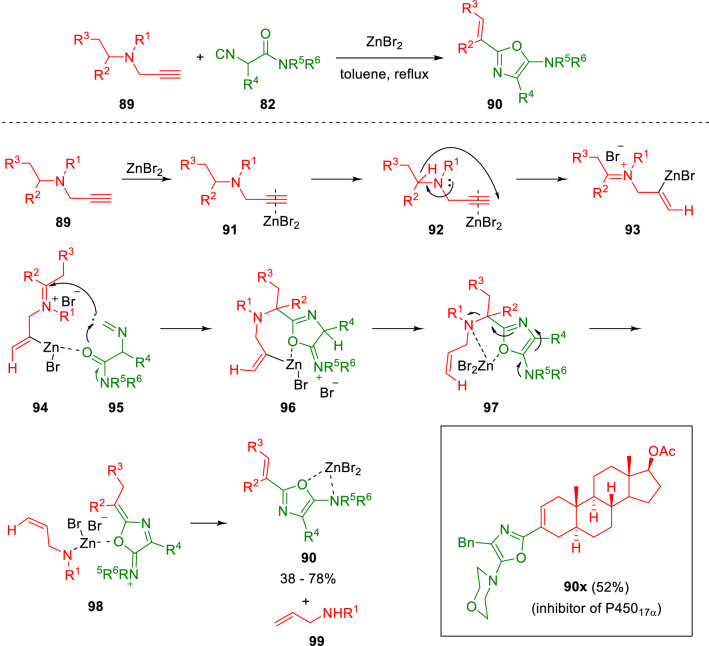
Propargylamines in oxazole synthesis from isocyanoacetamides

The substrate scope was then expanded by the introduction of propargylamines (**89**) to achieve alkenyloxazoles (**90**) with a novel substitution pattern in a reaction mediated by a stochiometric amount of ZnBr_2_ (Scheme 17) [29]. The transformation, wherein the propargylamine (**89**) acts as a vinyl cation synthetic equivalent, involves a domino sequence incorporating a 1,5-hydride shift, trapping of the in situ generated iminium salt (**93**) by the isocyanide (**82**), cyclisation to the corresponding oxazole (**96**), and 1,6-elimination to yield alkenyloxazole (**90**). This strategy has been used to prepare oxazoles incorporating a steroid skeleton, known to be potent P450_17α_ inhibitors.

**Scheme 18:**
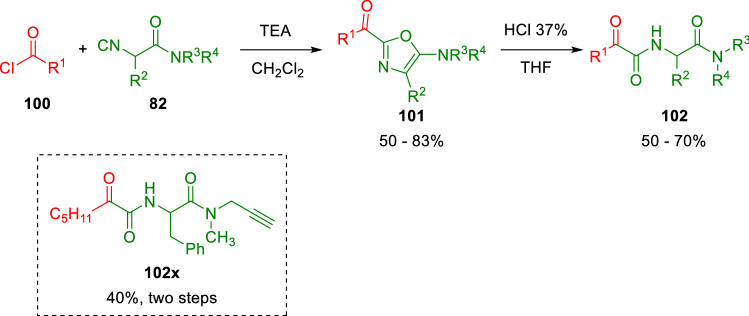
Synthesis of α-ketoamides from isocyanoacetamides

Furthermore, oxazole derivatives have been exploited as starting materials for post-condensation transformations. For example, α-ketoamides (**102**) can be produced through acid hydrolysis of 2-acyl oxazoles (**101**; Scheme 18) [30].

**Fig. 1:**
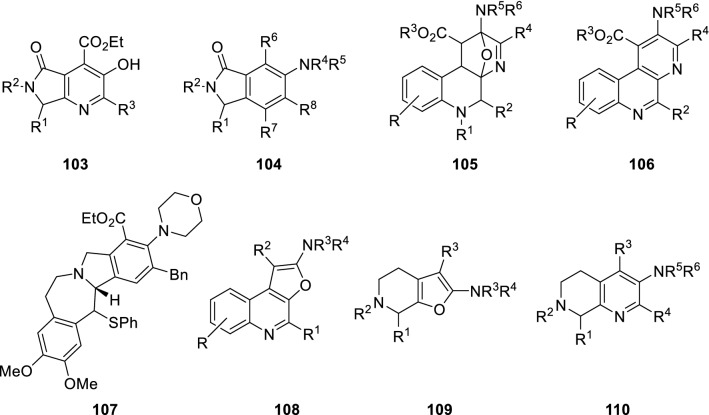
Heterocyclic scaffolds from oxazole-Diels–Alder reaction pathway

The oxazole scaffold can also be used as a diene in post-Ugi inter- or intramolecular Diels–Alder type cycloadditions. The dienophile is introduced, either as part of one of the starting materials of the Ugi condensation, usually the amine component, or incorporated as external reagent. This approach has been used for synthesis of different heterocycles and other complex compounds, such as pyrrolopyridines (**103**) [31], hexasubstituted benzenes (**104**) [32], tetrahydroquinolines (**105**) [33], phenantrolines (**106**) [34], polycyclic natural product-like scaffolds (**107**) [35], furoquinolines (**108**) [36], tetrahydrofuropyridines (**109**) [37], and naphthyridines (**110**) [38] (Fig. 1).

**Scheme 19:**
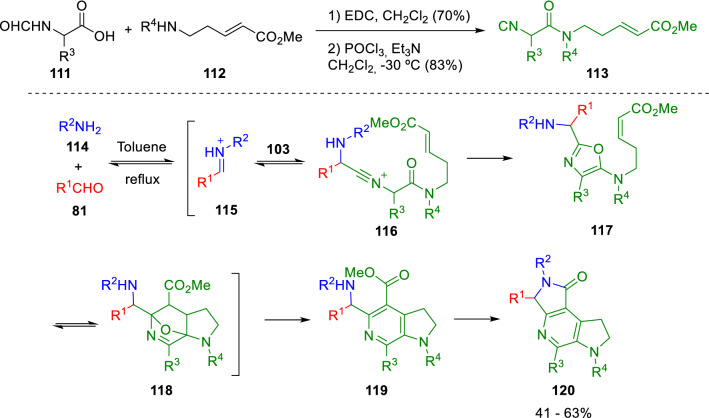
6-Azaindoline synthesis by isocyanoacetamide chemistry

Remarkably, Zhu and Fayol developed the synthesis of an isocyanoacetamide containing a dienophile motif (**113**) that made possible a post-Ugi Diels–Alder cycloaddition resulting in 6-azaindolines (**120**; Scheme 19) [39].

**Scheme 20:**
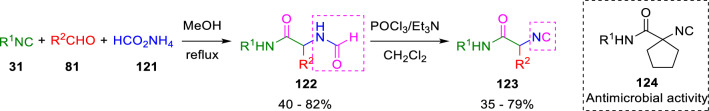
Isocyanide synthesis through post-condensation reaction of Ugi adduct

Aiming to access isocyanoacetamides of different natures, Marcaccini and collaborators have previously designed an alternative reaction pathway to synthesise novel isocyanoamides (**123**) through the dehydration of *N-*formamide Ugi adducts (**122**; Scheme 20) [40]. This work is a good example of the potential of the post-condensation transformation of Ugi adducts. Furthermore, the prepared isocyanoacetamides showed interesting biological properties, for example, cyclic isocyanoacetamides **124** showed good in vitro antimicrobial activity towards *C. albicans* and other microbial agents [41, 42]. More importantly, they are suitable substrates for further use in different isocyanide-based reactions.

**Scheme 21:**
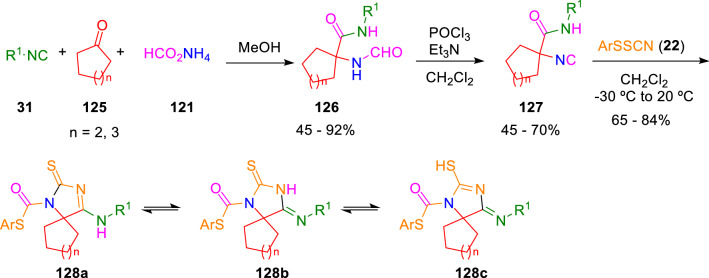
1,3-Diazaspiro-2-thiones construction through novel isocyanoamides

In fact, this ingenious strategy moved Marcaccini to explore more exotic isocyanoacetamides for heterocyclic construction. For instance, the condensation between novel isocyanide (**127**) and arylsulfenyl thiocyanates (**22**) furnished 1,3-diazaspiro-2-thiones (**128**), which can exist in three different tautomeric forms (Scheme 21) [43].

**Scheme 22:**
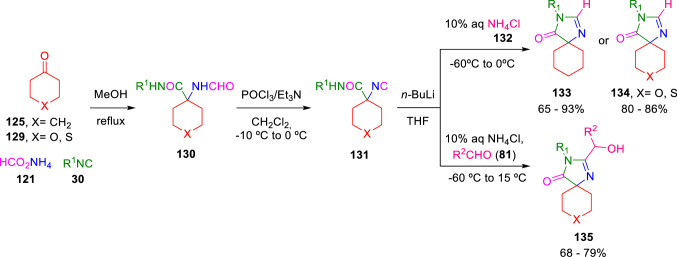
Synthesis of 2,3-disubstituted spiroimidazolones

In addition, 2,3-disubstituted spiroimidazolones (**133**–**135**) were formed by *n-*butyllithium treatment of isocyanoacetamides (**131**) and subsequent trapping of the resulting carbanion with NH_4_Cl (**132**) or aldehydes (**81**; Scheme 22) [43, 44].

**Scheme 23:**
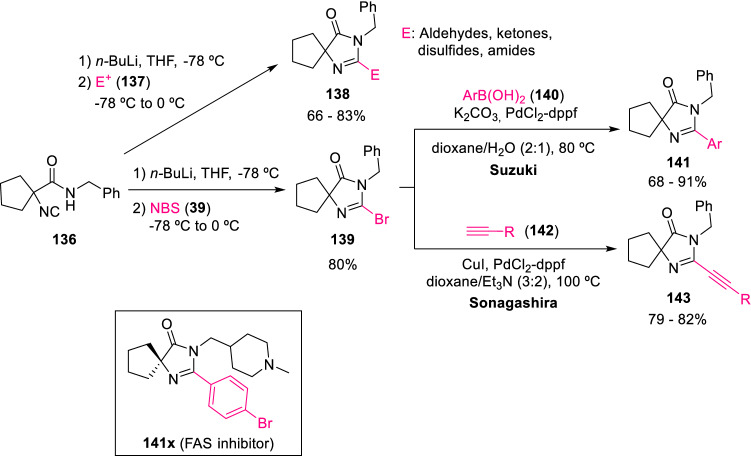
Diversity expansion of 2,3-substituted spiroimidazolones

Bischoff et al. expanded the scope of attainable 2,3-substituted spiroimidazolones by trapping the carbanion intermediate with electrophiles other than aldehydes, such as ketones, amides, or disulphides (**137**) to give 3-substituted spiroimidazolones (**138**). Furthermore, treatment of carbanion with NBS (**39**) introduced a bromine atom resulting in brominated imidazolones (**139**), which were then further subjected to Suzuki or Sonagashira couplings, broadening the diversity of spiroimidazolone products (**141**, **143**; Scheme 23) [45]. Bischoff also employed a palladium and copper catalysed C–H fuctionalisation of imidazolones to synthesise analogues of fatty acid synthetase (FAS) inhibitors (141x) [46].

**Scheme 24:**
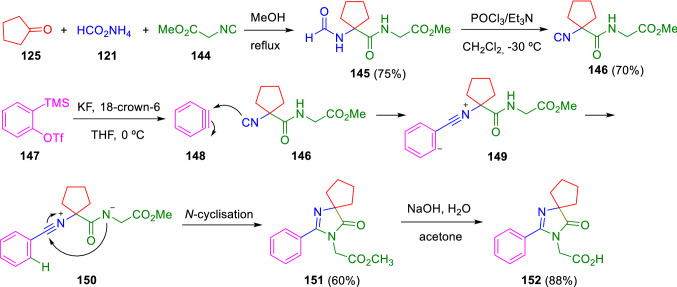
Key intermediate of CGRP antagonists’ synthesis by means of isocyanoamide from an Ugi adduct

An analogous strategy was performed by Pirali’s group in the construction of a key intermediate (**152**) in the synthesis of CGRP receptor antagonists. In this sequence, after the Ugi adduct dehydration, the corresponding isocyanoacetamide (**146**) was made to react with a suitable benzyne (**148**) to give intermediate (**149**), which affords the final imidazolone (**152**) through a cyclisation/hydrolysis sequence (Scheme 24) [47].

**Scheme 25:**
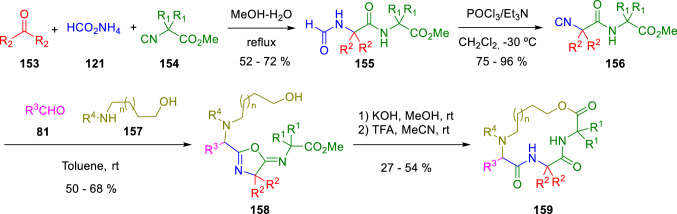
Macrocyclodepsipeptides construction employing isocyanoacetamides

Zhu and Pirali chose the same synthetic procedure to afford *α,α*-disubstituted *α*-isocyanoacetamides (**156**), which reacted in a three component reaction to give 5-iminooxazolines (**158**). Furthermore, the reaction between aldehydes (**81**), suitable amino alcohols (**157**) and isocyanoacetamides (**156**), followed by saponification and acid cyclisation resulted in different sized macrocyclodepsipeptides (**159**; Scheme 25) [48].

**Scheme 26:**
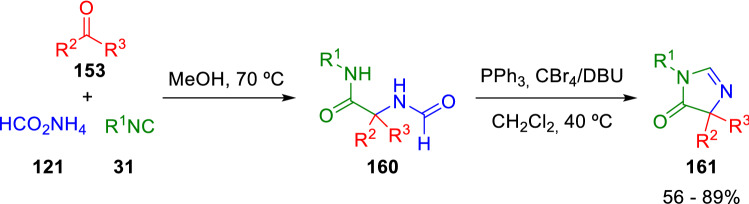
Ugi adduct N-formamide dehydration and cyclisation with novel base-dehydrating agent system

It is worthwhile to mention here some further studies that helped develop Marcaccini´s proposal. For example, Savic *et. al*. proposed the use of DBU as a base and a PPh_3_/CBr_4_ system as a dehydrating agent to transform *N-*formamide Ugi adducts (**160**) into 2-unsubstituted imidazolones (**161**; Scheme 26) [49]. Additionally, Meier et al. have recently revisited several isocyanide syntheses and have proposed *p-*TsCl as a cheaper and greener dehydrating agent [50].

### 2,2’-Diethoxy-isocyanoethane

**Scheme 27:**
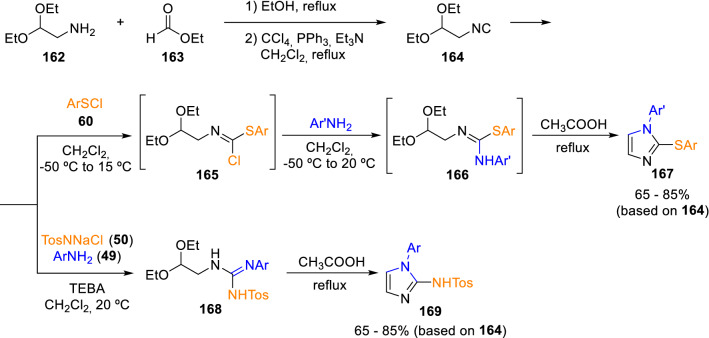
2,2-Diethoxyisocyanoethane in heterocyclic synthesis

Along with the use of isocyanoacetate (**1**) and isocyanoacetamides (**59**) in the construction of heterocyclic scaffolds, Marcaccini synthesised for the first time 2,2-diethoxy-1-isocyanoethane (**164**) through dehydration of the corresponding *N-*formamide. He then reacted this novel isocyanide with sulphur electrophiles (**60**) or chloramine T (**50**), followed by an intramolecular cyclisation in acidic medium, to synthesise substituted imidazole cores (**167** and **169**) (Scheme 27) [51, 52].

**Scheme 28:**
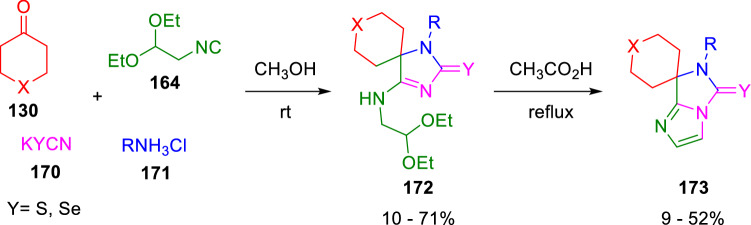
Synthesis of spiro spiroimidazo[1,5-*a*]imidazole-5-thiones

Marcaccini also reacted 2,2-diethoxy-1-isocyanoethane (**164**) in an Ugi four-component condensation (U-4CC) together with cycloketones (**130**), amine hydrochlorides (**171**) and potassium thiocyanate or selenocyanate (**170**), to obtain spiroimidazo[1,5-*a*]imidazole-5-thiones (**173**) in the presence of acetic acid (Scheme 28) [53].

**Scheme 29:**
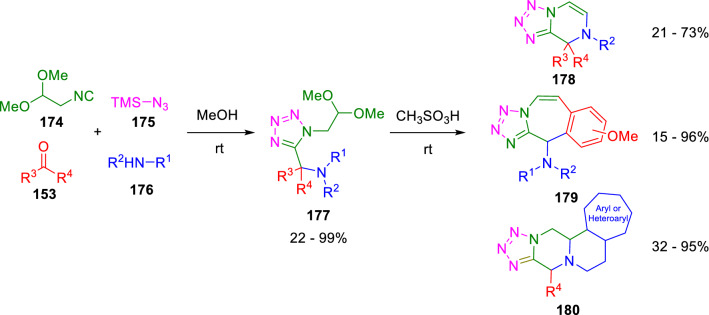
Synthesis of diverse heterocyclic scaffolds employed 2,2-diethoxyisocyanoethane

2,2-Dialkoxy-1-isocyanomethanes (**174**) have facilitated the access to different heterocyclic skeletons. They were even categorised as universal isocyanides for heterocyclic synthesis by Dömling in 2014, who synthesised several Ugi tretrazole derivatives (**178**, **179**, **180**) using **174** in an Ugi-tetrazole reaction followed by acid mediated cyclisation of the Ugi adduct (**177**; Scheme 29) [54].

**Scheme 30:**
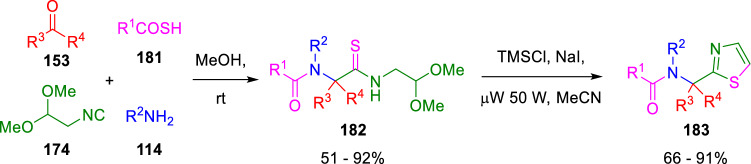
Kazmaier’s synthesis of thiazoles

Kazmaier synthesised thiazoles (**183**) following a thio-Ugi/cyclisation sequence, in which the ring closing stage was similar to Marcaccini´s works (Scheme 30) [55], demonstrating the synthetic potential of this isocyanide (**174**).

**Scheme 31:**
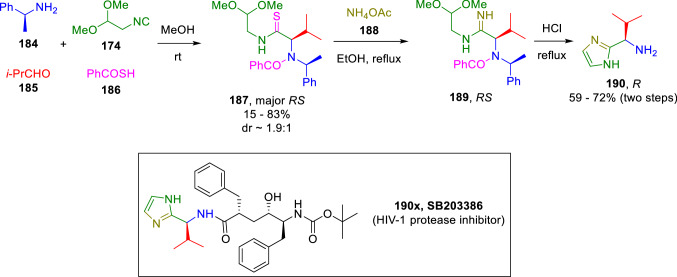
Nenanjdenko’s synthesis of chiral imidazoles

Moreover, chiral imidazoles (**190**) have been diastereoselectively obtained by Nenanjdenko using a similar strategy (Scheme 31) [56]. This reaction readily provides a key intermediate in the synthesis of orally bioactive HIV-1 protease inhibitor SB203386 (190x).

**Scheme 32:**
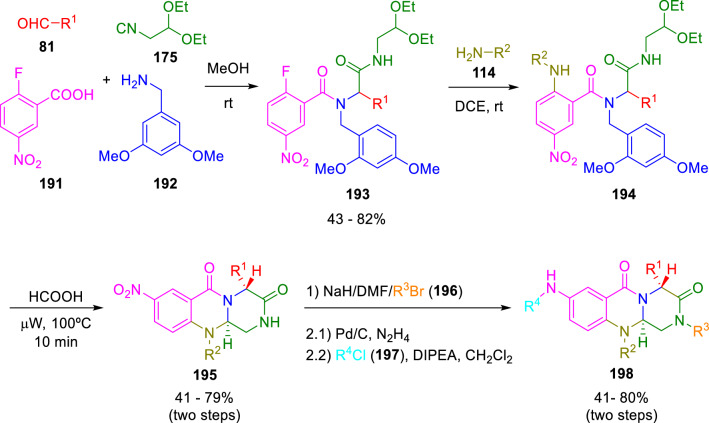
Synthesis of brevianamides M–N and fumiquinazolines A-C via isocyanide chemistry

Hulme and Gunawan also took advantage of diethoxyisocyanoethane (**174**) synthetic features to achieve an Ugi/*N*-acyliminium ion cyclisation cascade to afford tricyclic system (**198**), characteristic of marine alkaloids brevianamides M–N and fumiquinazolines A-C (Scheme 32) [57].

### Alkyl (*Z*)-3-(dimethylamino)-2-isocyanoacrylates

Alkyl (*Z*)-3-dimethylamino-2-isocyanoacrylates (**199**), or Schöllkopf´s isocyanides, were synthesised by Meerwein in 1961 [58]. These are highly versatile isocyanides for heterocyclic synthesis that enable post-condensation transformations of MCR adducts, due to concurrence of isocyanide, alkene and ester functionalities together with a Michael acceptor and a dimethylamino leaving group [59].

**Scheme 33:**
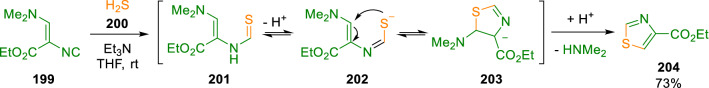
Thiazole construction from alkyl-(*Z*)-2-dimethylamino-2-isocyanoacrylate

Schöllkopf, popularised their use [2] and reported their first application in heterocyclic synthesis [60], employing (**199**) and H_2_S in the preparation of thiazole (**204**; Scheme 33). The mechanism of this reaction involves the formation of an intermediate methanethioamide (**201**) and the subsequent intramolecular Michael cyclisation with elimination of dimethylamine.

**Scheme 34:**
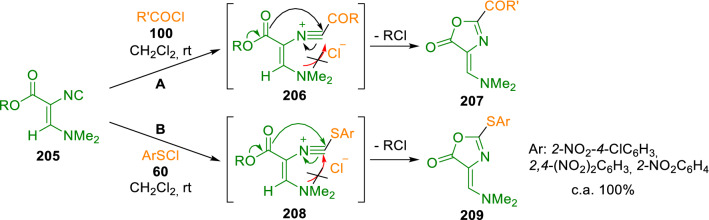
Synthesis of substituted oxazoles

Schöllkopf also reported the formation of imidazole rings from isocyanide (**199**) and alkyl or acyl halides [60, 61]. However, Marcaccini et al*.* revisited this synthesis and showed that the structures proposed by Schöllkopf were incorrect. Instead, substituted oxazoles (**207**) were shown to be obtained by reacting alkyl (*Z*)-3-dimethylamino-2-isocyanoacrylates (**205**) with acyl chlorides (**100**), as evidenced by their physical and spectral properties [62] (Scheme 34 A). Moreover, Marcaccini also reported the formation of oxazoles (**209**) when electron-deficient arylsulfenyl chlorides (**60**) were used as electrophiles. The mechanism of this reaction involves the attack of the isocyanide (**205**) on the electrophile and a subsequent ring closing step concerning not the dimethylamino group, but instead the ester group (Scheme 34) [63]. As far as we know, Marcaccini et al. were the only research group that reported oxazole synthesis from Schöllkopf´s isocyanide.

**Scheme 35:**
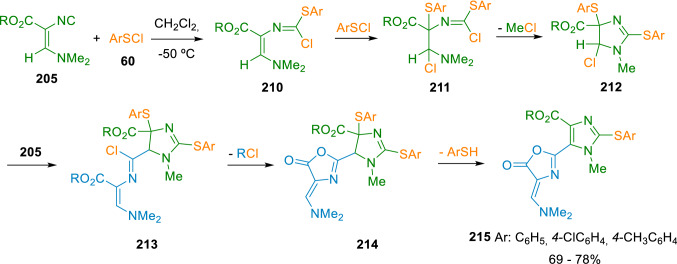
Synthesis of imidazolyloxazolones

Marcaccini et al. also attempted to obtain the imidazole core from Schöllkopf’s isocyanide. Thus, in contrast with previous results, reaction of isocyanide **205** with relatively electron-rich arylsulfenyl chlorides (**60**) gave access to the desired imidazole ring, which was then trapped by a second isocyanide molecule and further evolved into the final imidazolyloxazolones (**215**; Scheme 35) [64].

**Scheme 36:**
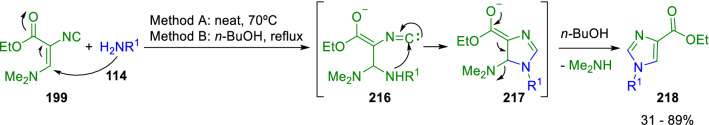
Imidazole synthesis from Schöllkopf´s isocyanide

The imidazole scaffold was also achieved by Helal and Lucas, by coupling Schöllkopf´s isocyanide (**199**) with primary amines (**114**). The reaction takes place with hindered or unhindered amines and proceeds with high regioselectivity and good functional group tolerance, achieving the final products (**218**) in moderate to good yields (Scheme 36) [65].

**Scheme 37:**
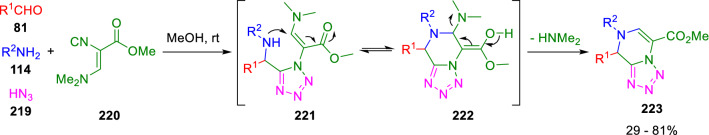
Synthesis of bicyclic pyrazines

Moreover, pyrazine core could be built through alkyl (*Z*)-3-dimethylamino-2-isocyanoacrylate chemistry. For example, Bienaymé and Bouzid achieved pyrazines (**223**) in one pot through an Ugi-tetrazole reaction from Schöllkopf´s isocyanide (**220**). In this process, an intramolecular Michael addition took place on the Ugi adduct (**221**) with a release of Me_2_NH to get the final product (Scheme 37) [66].

**Scheme 38:**
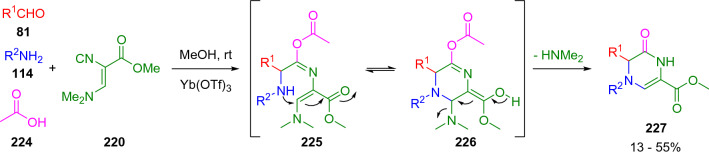
Pyrazine construction employing Schöllkopf´s isocyanide

Later, Illgen et al. [67] developed an Ugi reaction with Schöllkopf´s isocyanide (**220**) for pyrazine construction. In this case, the reaction pathway did not involve a Mumm rearrangement of the primary adduct (**225**), but instead an intramolecular Michael addition led to the final pyrazine (**227**; Scheme 38).

**Scheme 39:**
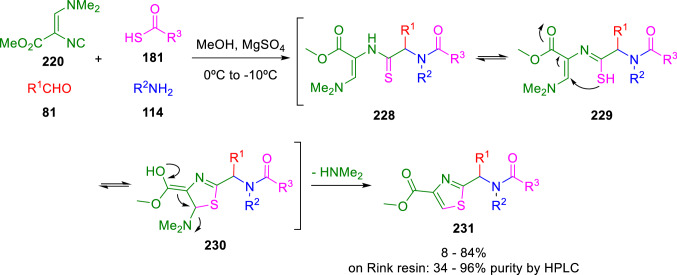
Ugi post-condensation reaction for thiazole synthesis from Schöllkopf´s isocyanide

Schöllkopf´s isocyanide (**220**) also readily gives Ugi and Passerini condensations with thiocarboxylic acids (**181**) [59, 68] and the resulting adducts (**228**) have been extensively employed to synthesise thiazoles by the post-condensation transformations. Hence, Dömling reported a thiazole (**231**) synthesis by the cyclisation of the mercaptoimine tautomer (**229**) of the Ugi adduct (**228**; Scheme 39) [69, 70]. This reaction has also been performed on solid support in a combinatorial fashion for the construction of chemical libraries [71].

**Scheme 40:**
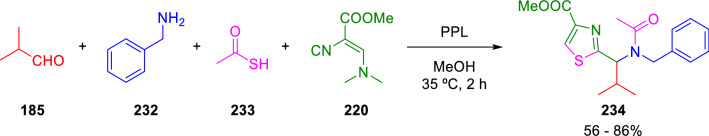
Synthesis of 2,4 substituted thiazoles by isocyanide chemistry catalysed by PPL

Recently, an enzyme-catalysed version of this synthesis has been reported by Zhang, (Scheme 40) [72]. Porcine pancreatic lipase (PPL) showed good catalytic activity, allowing the reaction to take place in mild conditions and improving yields of thiazole (**234**) in a reduced reaction time.

**Scheme 41:**
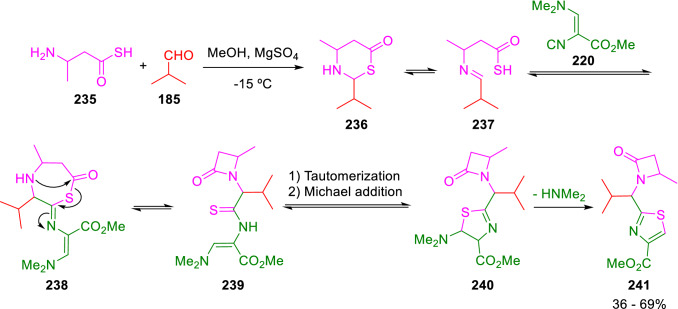
Synthesis of 1-thiazole-2-yl-methyl-azetidin-2-ones

Moreover, 1-thiazole-2-yl-methyl-azetidin-2-ones (**241**) were obtained by means of a U-4CC between Schöllkopf´s isocyanide (**220**), aldehydes (**185**) and β-aminothiocarboxilic acids (**235**) that facilitated the formation of a β-lactam ring through a 7-membered intermediate (**238**). Then, the thiazole is formed via Michael addition and elimination of Me_2_NH, as previously described (Scheme 41) [73].

**Scheme 42:**
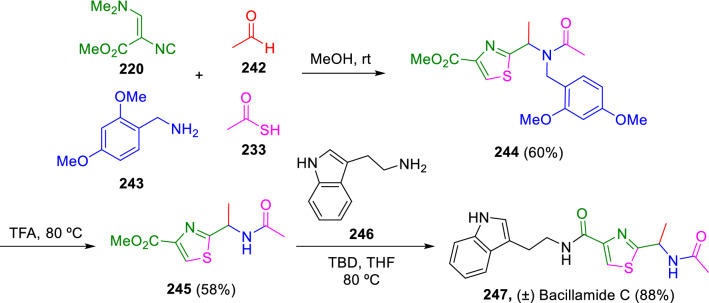
Synthesis of Bacillamide C analogues

Dömling applied an Ugi reaction with Schöllkopf´s isocyanide (**220**) to construct analogues of Bacillamide C (**247**), a microbial natural product with algicide and antibacterial bioactivity. The synthetic pathway is described in Scheme 42 [74].

**Scheme 43:**
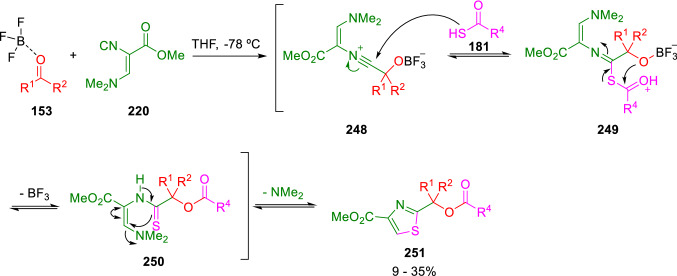
Passerini condensation to thiazole construction

Additionally, disubstituted thiazoles (**251**) have also been synthesised by a Passerini three-component condensation (P-3CC) of oxo-compounds (**153**), thiocarboxylic acids (**181**) and methyl (*Z*)-3-dimethylamino-2-isocyanoacrylate (**220**) under BF_3_·OEt_2_ Lewis acid catalysis, (Scheme 43) [75].

**Scheme 44:**
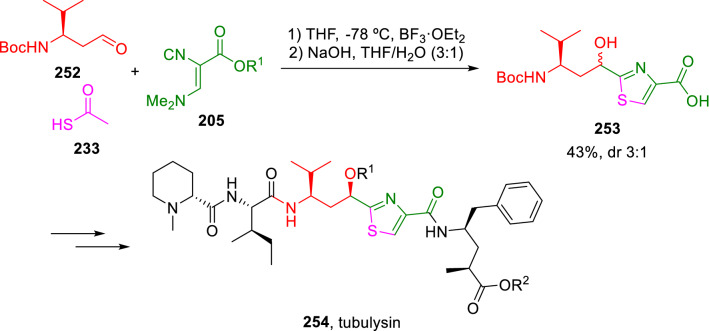
Total synthesis of tubulysin

This strategy was used as a key step in the total synthesis of antimitotic tubulysin analogues (**254**), as shown in Scheme 44 [76, 77].

### 2-Isocyanothioanisole

**Scheme 45:**
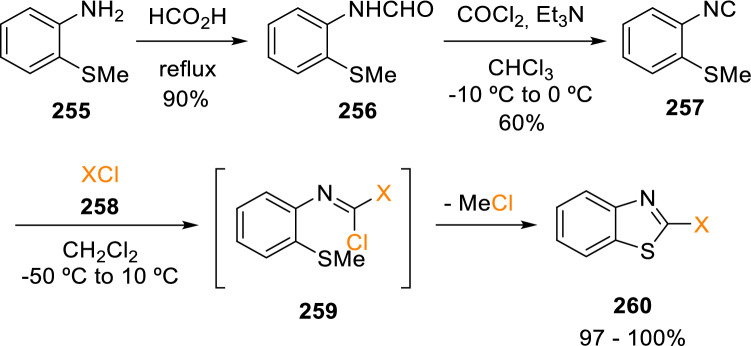
2-Functionalised benzothiazoles from 2-isocyanothioanisole

Aiming to obtain 2-functionalised benzothiazoles, Marcaccini et al. synthesised a novel isocyanide, 2-isocyanothioanisole (**257**). This compound reacts with different electrophiles (**258**) to form intermediates (**259**), which cyclise to yield 5-membered heterocycles (**260**; Scheme 45) [78].

Recently, the chemistry of 2-isocyanothioanisole (**257**) has reached popularity due to its implication in benzothiazole synthesis by means of radical or photochemical approaches.

**Scheme 46:**
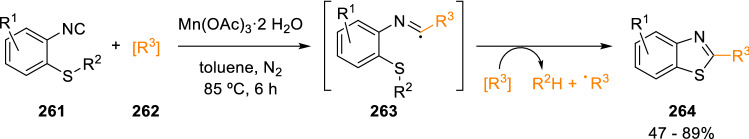
Radical synthesis of benzothiazoles

Wu et al. described the first example of an imidoyl radical coupling with sulphur atom on 2-isocyanoaryl thioethers (**261**). This radical cyclisation can be triggered by a broad scope of radical precursors, such as phosphorus oxides or alkyl radical precursors, yielding 2-substituted benzothiazoles (**264**) in good yields and broad functional group tolerance (Scheme 46) [79].

**Scheme 47:**
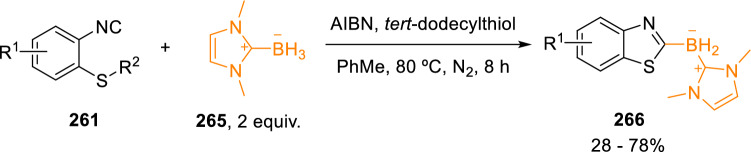
Benzothiazole synthesis employing a radical borylative cyclisation

Similarly, Liu’s group synthesised 2-borylated benzothiazoles (**266**) through radical borylative cyclisation of 2-isocyanothioanisole (**261**) with an *N*-heterocyclic carbene borane (**265**) using AIBN as radical precursor (Scheme 47) [80].

**Scheme 48:**
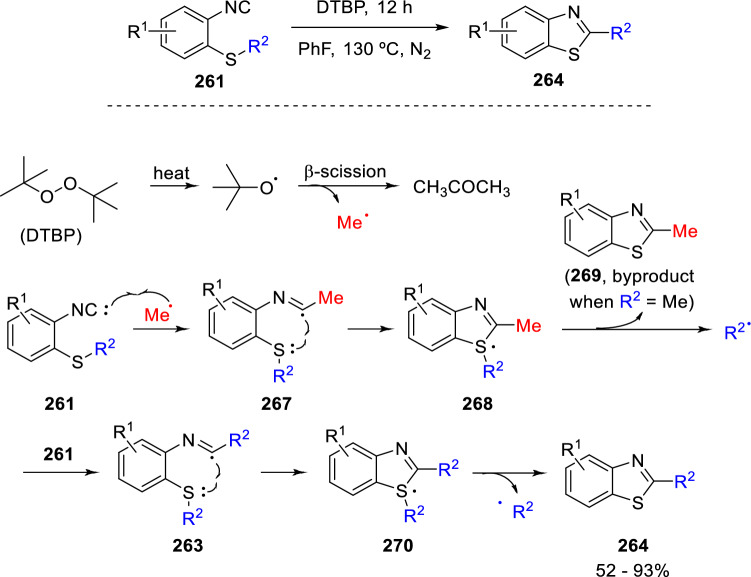
Radical synthesis of benzothiazoles by reinstallation of the alkyl substituent

An interesting and more atom-economical alternative was also conceived by Wu and collaborators. This radical cascade employs di-*tert*-butyl peroxide (DTBP) as radical precursor and achieves an intramolecular S to C transfer of the R^2^ group, which produces the final 2-substituted benzothiazoles (**264**; Scheme 48) [81].

**Scheme 49:**
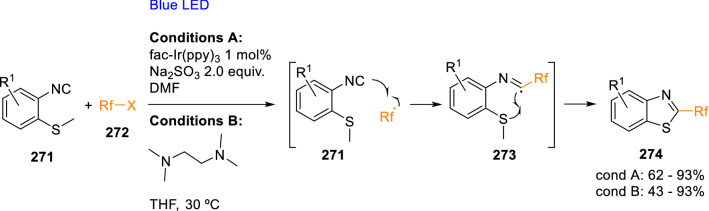
Photochemical synthesis of benzothiazoles

Radical couplings of 2-isocyanoaryl thioethers (**271**), which produce 2-substituted benzothiazoles (**274**), have also been carried out in photochemical conditions. For example, Yuan et al. synthesised benzothiazoles with fluorine containing motifs in position two (**274**) using visible light, an Ir^4+^/Ir^3+^ photocatalyst and Na_2_SO_3_ as a reductant (Scheme 49, *conditions A*) [82]. In a similar way, blue light-mediated fluoroalkylation to obtain substituted benzothiazoles (**274**) was performed with fluoroalkyl iodides using tetramethylethane-1,2-diamine (TMEDA) as electron donor to promote radical coupling with 2-isocyanothioanisole (**271**; Scheme 49, *conditions B*) [83].

**Scheme 50:**
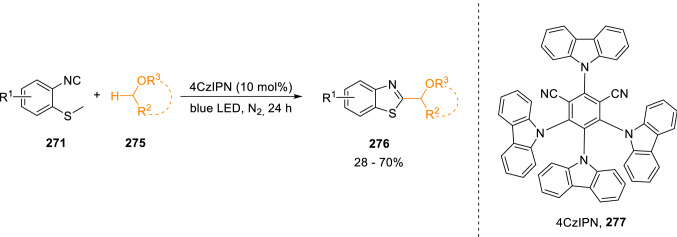
Photosynthesis of 2-benzothiazoles from 2-isocyanothioanisole and ethers

Recently, 4CzIPN (**277**) has gained attention as a photocatalyst for irradiation with blue light to form benzothiazoles from 2-isocyanoaryl thioethers (**271**). Wu et al. have designed a metal-free, oxidant-free protocol for the synthesis of 2-substitued benzothiazoles (**276**) based on a photocatalysed reaction between (**271**) and cyclic or acyclic ethers (**275**) through a SET pathway (Scheme 50) [84].

Lastly, Liu and collaborators have developed a phosphorus radical cascade employing their catalyst 4CzIPN-*t*Bu, a modification of 4CzIPN (**277**) in which *t*-Bu groups have been introduced on positions 3 and 6 of each carbazole motif. They used this catalyst to perform a PECT cycle to obtain 2-phosphorylated benzothiazoles from 2-isocyanoaryl thioethers (**271**), a reaction that was previously performed without photochemical activation [79]. Notably, they were also able to synthesise diverse heterocycles, such as phenanthridines or quinolines using different isocyanides [85].

## Intramolecular Ugi and Passerini reactions

Intramolecular Ugi reactions are possible when one of the starting materials contains two of the functional groups involved in the reaction. This strategy has been employed by numerous groups to increase the scaffold diversity of the classical Ugi reaction. Marcaccini developed diverse original intramolecular Ugi and Passerini reactions using bifunctional starting materials. In this way, he was able to prepare libraries of different privileged structures with relevance in the field of medicinal chemistry and chemical biology. These developments have had an important influence in the further contributions of other research groups [86, 87].

**Scheme 51:**
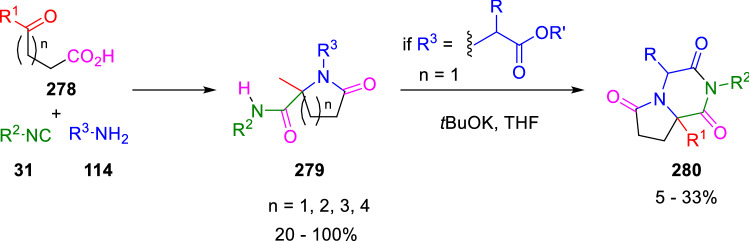
First examples of the use of oxoacids in the Ugi reaction

The use of oxocarboxylic acids (**278**) as bifunctional stating material in IMCR was developed by Short [88] and Harriman [89] for the preparation of five-, six-, seven- and eight-membered lactams (**279**) through Ugi four-centre 3-component reactions (U-4C-3CR; Scheme 51). Similarly, Ugi prepared diverse γ-lactams by using levulinic, 3-benzoylpropionic and phthalaldehydic acids. When amino esters are used as the amine component, it is possible to obtain 1,4-diazabicyclo[4.3.0]nonane-3,5,9-triones (**280**) [90].

**Scheme 52:**
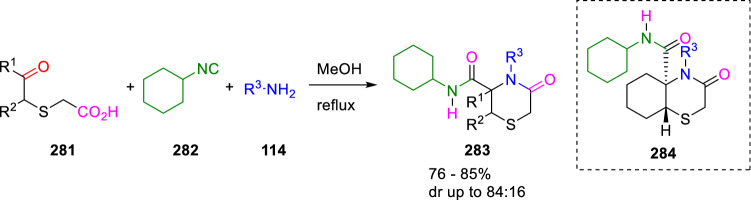
Ugi reaction of 5-oxo-3-thiacarboxylic acids

Stefano Marcaccini described for the first time the Ugi four-centre three-component reaction (U-4C-3CR) between 5-oxo-3-thiacarboxylic acids (**281**), benzylamines (**114**) and cyclohexyl isocyanide (**282**). The reaction takes place in refluxing methanol giving 5-oxothiomorpholine-3-carboxamides (**283**) with good yields and high diastereoselectivities (Scheme 52). The major diastereoisomer has been assigned *trans* configuration by NOESY experiments of the bicyclic compounds (**284**) [91].

**Scheme 53:**
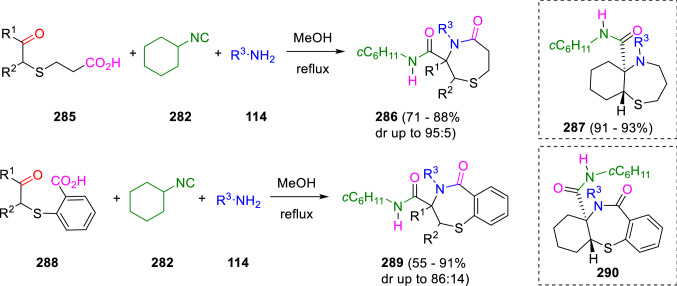
Intramolecular Ugi condensation of 6-oxo-4-thiacarboxylic acids

This strategy was extended to U-4C-3CR of 6-oxo-4-thiacarboxylic acids (**285**) to form biologically relevant hexahydro-1,4-thiazepin-5-ones (**286**) and 1,4-benzothiazepin-5-ones (**289**). This condensation takes place in some cases with high stereoselectivity (Scheme 53) [92]. Furthermore, chemoselective reduction with LiAlH_4_/AlCl_3_ of the cyclic carbonyl group gives bicyclic 1,4-thiazepine-3-carboxamides (**287**).

**Scheme 54:**
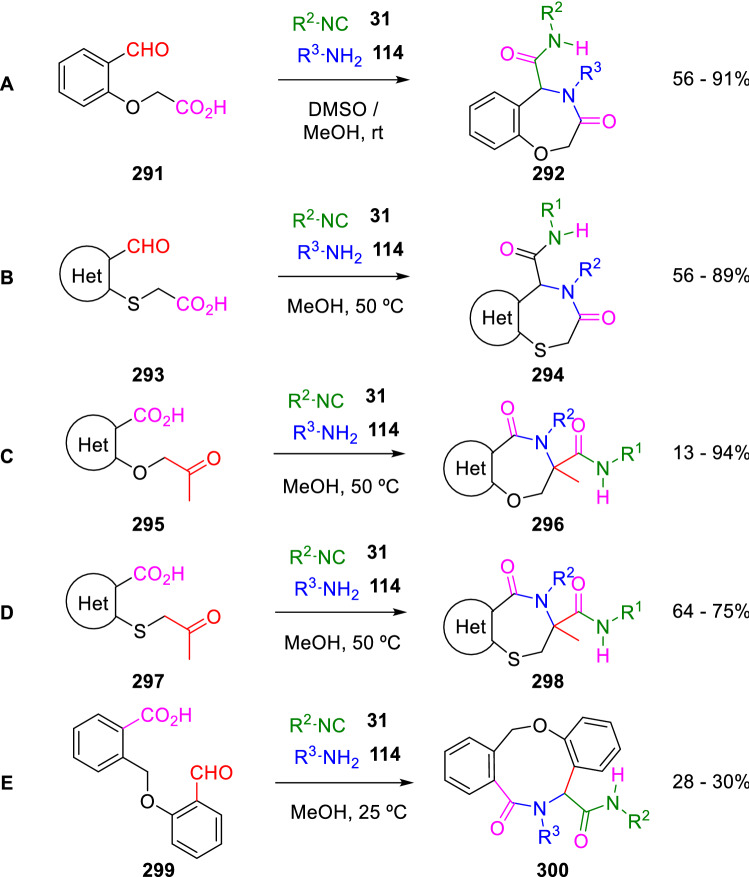
Synthesis of oxazepine and thiazepine-fused carbocycles and heterocycles

Ivachtchenko used a similar protocol to synthesise 3-oxo-1,4-thiazepine-5-carboxamides (**294**) [93], 5-oxo-1,4-oxazepine-3-carboxamides (**296**) [94] and 5-oxo-1,4-thiazepine-3-carboxamides (**298**) [93] fused to diverse heterocycles from the oxoacids (**293**), (**295**) and (**297**), respectively (Scheme 54, B–D). He also obtained nine-membered heterocyclic scaffolds (**300**) by the reaction of the aromatic aldehyde-acid (**299**; Scheme 54, E) [95]. As well, Zhang et al. have previously used different oxoacids, including 2-(2-formylphenoxy)acetic acid (**291**) to prepare diverse lactams and oxazepines, such as **292** (Scheme 54, A) [96].

**Scheme 55:**
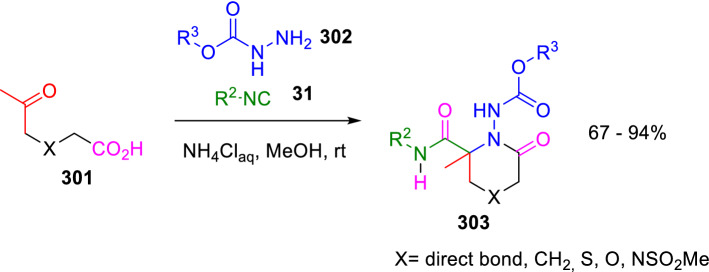
Synthesis of *N*-amino lactams

On the other hand, the reaction of similar ketoacids (**301**) with isocyanides (**31**) and *Boc-* or *Cbz-*protected hydrazine (**302**) was used by Krasavin for the synthesis of *N*-aminolactams (**303**), which are proline-like *β*-turn secondary structure mimics (Scheme 55) [97].

**Scheme 56:**
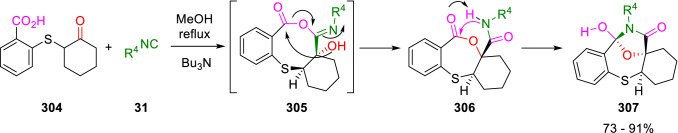
Intramolecular Passerini condensation to prepare tetracyclic 1,4-benzothioxepin orthoamides

Marcaccini performed an intramolecular Passerini condensation between oxothiocarboxylic acid (**304**) and isocyanides (**31**) under tertiary amine catalysis. This reaction affords seven-membered lactones (**306**) that suffer spontaneous intramolecular nucleophilic attack of the amide NH to give unexpected tetracyclic 1,4-benzothioxepin orthoamides (**307**). The observed stereochemistry of the major product is due to an axial attack of the isocyanide to yield a *cis*-fused α-adduct (**305**), which, evolves to give the *trans*-fused Passerini adduct (**306**; Scheme 56) [92]. This is an appealing result as the *N*-amidoalkoxycarbinol moiety (orthoamide) was considered an unstable intermediate in the reaction of an ester with an amide anion or an imide with an alkoxy anion. At the time, the only known stable compounds having this structural feature were peptide ergot alkaloids (ergopeptines).

**Scheme 57:**
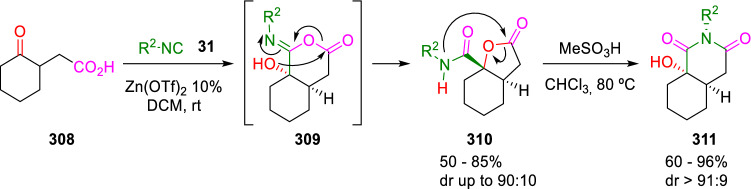
Synthesis of lactones by intramolecular Passerini reaction

In contrast, Orru and Ruijter found that when the ketoacid (**308**) or other γ- and δ-keto acids are used in a Passerini three-centre two-component reaction (P-3C-2CR), the corresponding *trans*-fused lactone (**310**) is obtained with good diastereoselectivity. Interestingly, these Passerini adducts (**310**) are readily rearranged to less strained *cis*-fused α-hydroxyimides (**311**) under acidic conditions (Scheme 57) [98].

**Scheme 58:**
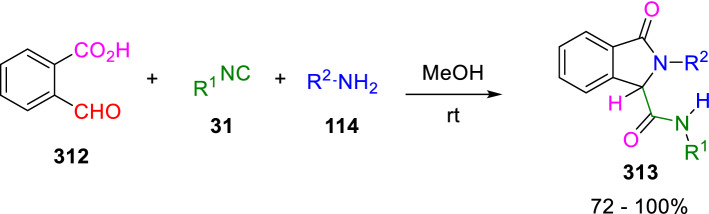
Synthesis of isoindolinone-carboxamide derivatives

As with other oxoacids, 2-formylbenzoic acid (**312**) was reported to suffer U-4C-3CRs with amines (**114**), and isocyanides (**31**) to give 2-isoindolinone-7-carboxamide analogues (**313**; Scheme 58) [96, 99].

**Scheme 59:**
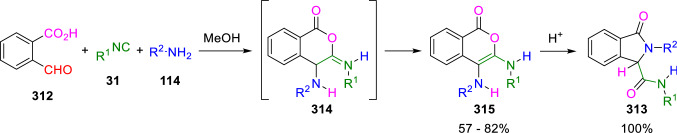
Obtention of isochromenone Ugi primary adduct and they transformation

Remarkably, Marcaccini developed an interrupted Ugi condensation from 2-formylbenzoic acid (**312**), scarcely basic amines (**114**) and isocyanides (**31**). In this reaction, as in the regular Ugi condensation, the nitrilium intermediate is intramolecularly attacked by the carboxylic acid to give the usual primary adduct (**314**). However, in this case, this intermediate does not suffer a Mumm rearrangement, but is instead stabilised by a tautomerisation leading to isochromenone enediamine (**315**). In this process, the precise control of the reaction conditions (solvent, time, and temperature) is essential to obtain the products (**315**) satisfactorily. In some cases, isocoumarins (**315**) in the presence of a catalytic amount of acid suffer a rearrangement to give isoindolines (**313**) in quantitative yield (Scheme 59) [100].

This reaction was later reported by Ramazani to be catalysed by silica nanoparticles in solvent-free conditions, though the amine component was limited in this case to dibenzylamine [101]. The same protocol was also used to synthesise isochromenone-functionalised mesoporous silica hollow spheres from 2-formylbenzoic acid (**312**), 2,6-dimethylphenyl isocyanide and amine-containing silica spheres [102].

**Scheme 60:**
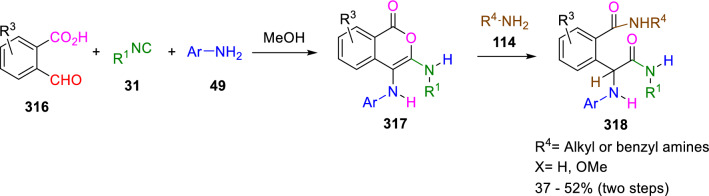
Synthesis of phenylglycine derivatives from primary Ugi adducts

Marcaccini also reported the reaction of isocoumarin enediamines (**317**) with amines (**114**), which promote a ring cleavage that gives new phenylglycine derivatives (**318**) in almost quantitative yields (Scheme 60). This process allows four diversity elements to be introduced through an U-4C-3CR. The reaction was performed in solventless conditions using an excess of the amine to prevent the decomposition of labile isocoumarins (**317**) [103].

**Scheme 61:**
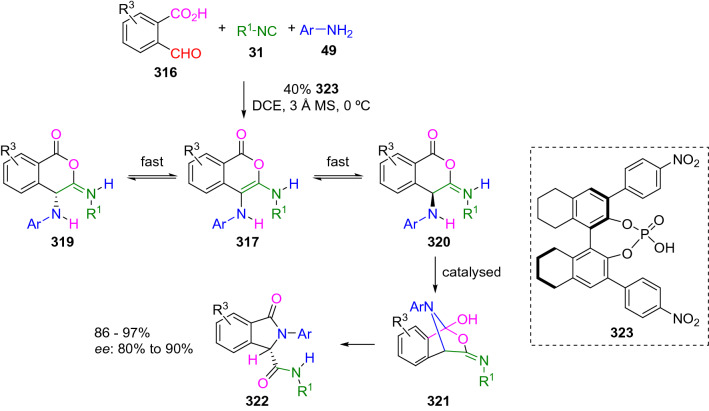
Enantioselective intramolecular Ugi reaction

An enantioselective version of this process has been described by Zhu using an octahydro (*R*)-BINOL-derived chiral phosphoric acid (**323**). The reaction of 2-formylbenzoic acids (**316**), isocyanides (**31**), and aromatic amines (**49**) in the presence of a catalytic amount of chiral phosphoric acid (**323**) affords the *S* isoindoline (**322**) with good enantioselectivity. The authors justify the observed enantioselectivity by a dynamic kinetic resolution process in which there is an imine-enamine tautomerisation equilibrium, much faster than acid-catalysed Mumm rearrangement (Scheme 61) [104].

**Scheme 62:**
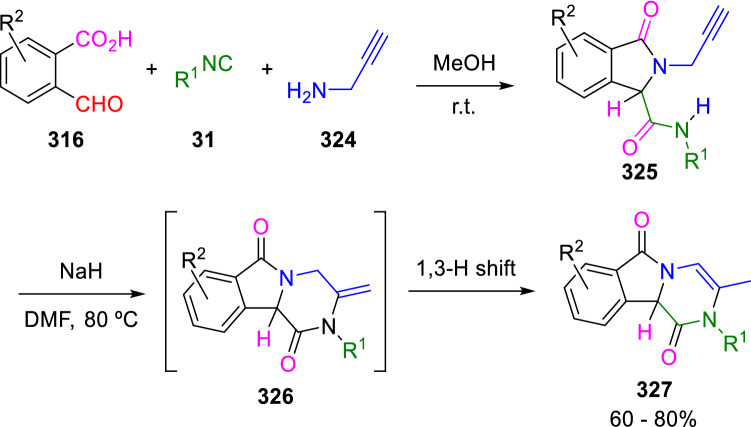
Synthesis of pyrazino[2,1-*a*]isoindolediones

When the propargyl amine (**324**) is used in the Ugi reaction of 2-formylbenzoic acids (**316**) and isocyanides (**31**), it is possible to obtain the pyrazino[2,1-*a*]isoindolediones (**327**) by 6-*exo-dig* intramolecular hydroamination on the Ugi adducts (**325**), followed by a 1,3-H shift (Scheme 62) [105].

**Scheme 63:**
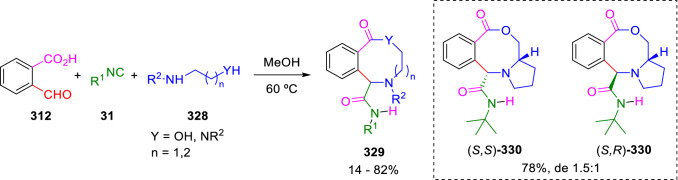
Synthesis of eight and nine-membered lactones and lactams

The reaction of 2-formylbenzoic acid (**312**) with isocyanides (**31**) and amino alcohols or bis-secondary amines (**328**) in methanol, under microwave irradiation, at 60 ºC permits the synthesis of eight and nine-membered lactones or lactams (**329**), probably through an isochromenone enediamine. When *L*-prolinol is used as the amine component, diastereomeric lactones (**330**) are obtained in a 1.5:1 ratio (Scheme 63) [106].

**Scheme 64:**
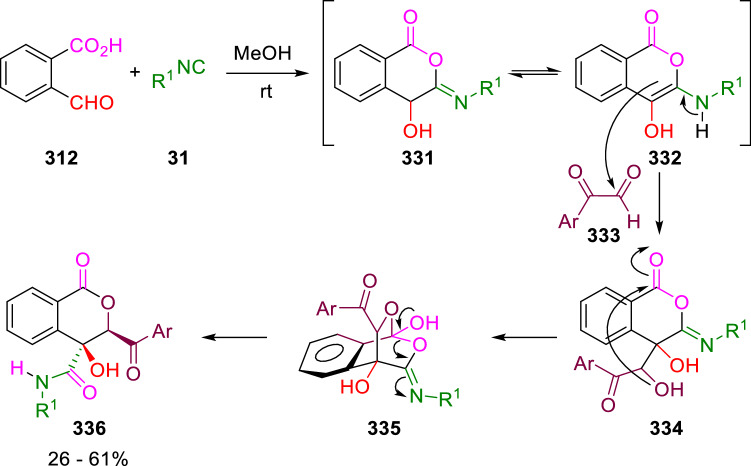
Synthesis of isocumarins by tandem P-3C-2CR / aldol reaction

A combination of a P-3C-2CR of 2-formylbenzoic acid (**312**) and isocyanides (**31**) and a subsequent aldol condensation with arylglioxals (**333**) was reported by Jiang and Tu. The Passerini reaction takes place in methanol to give an intermediate enamine (**332**). This reacts with arylglioxal (**333**) through an aldol condensation to give hydroxyaldehyde (**334**), which then undergoes an intramolecular nucleophilic addition and a ring-opening process to give the isocoumarins (**336**; Scheme 64) [107].

**Scheme 65:**
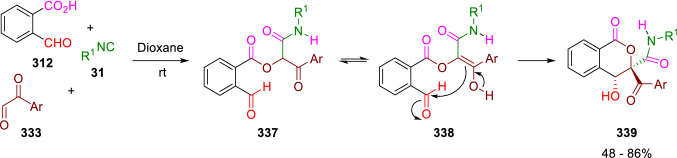
Synthesis of isocumarins by P-3CC followed by aldol reaction

The authors argue that arylglyoxals (**333**) are mainly in their hydrate form in methanol and therefore are not prone to participate in an intermolecular P-3CC. On the other hand, when the reaction is carried out in dioxane, arylglyoxals exist mostly in the aldehyde form and a P-3CC takes place swiftly to give (**337**), which then evolves through an intramolecular aldol reaction to yield isomeric isocoumarin (**339**; Scheme 65) [107].

**Scheme 66:**
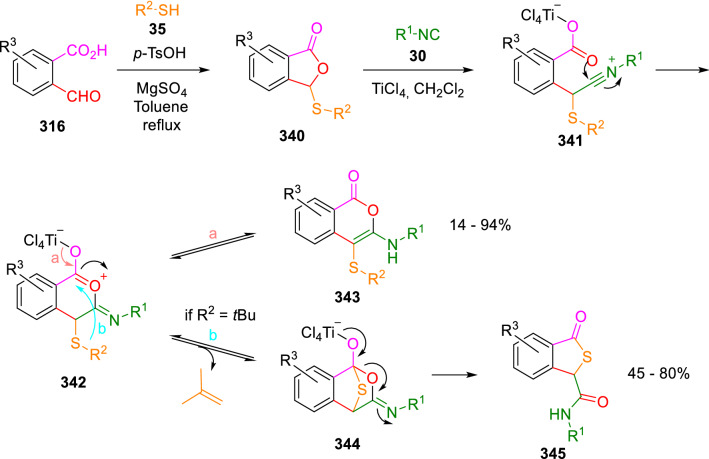
Reaction of formylbenzoic acids, thiols and isocyanides

A divergent preparation of isocoumarins (**343**) and thiophthalides (**345**) was described by El-Kaïm. Oxoacid (**316**) reacts with thiols (**35**) in the presence of *p*-toluenesulfonic acid catalyst and magnesium sulphate to give 3-sulfanyl-phthalides (**340**), which suffer an insertion reaction of isocyanide (**31**) mediated by titanium tetrachloride to give the isocoumarines (**343**). When *tert*-butyl thiol (**35**, R^2^ = ^*t*^Bu) is used, thiophthalides (**345**) are obtained by a formal thio-Passerini reaction. The deprotection of the *tert*-butyl group in intermediate (**342**) leads to the thiophthalides (**345**) by a 1,5-Mumm rearrangement. The process is also possible in one pot (Scheme 66) [108, 109].

**Scheme 67:**
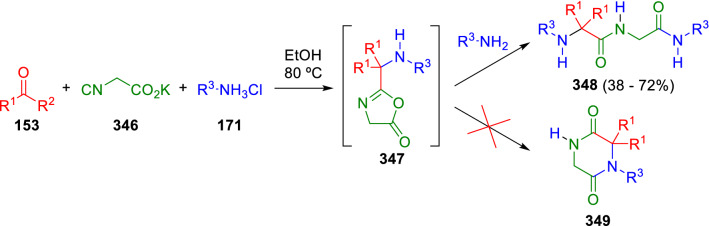
Synthesis of dipeptides from potassium isocyanoacetates

The experience with isocyanoacetates (**1**) in the synthesis of heterocycles, as mentioned above, surely encouraged Marcaccini to use isocyanoacetic acid in Ugi-type reactions. For example, an intramolecular Ugi reaction between ketones (**153**), amine hydrochlorides (**171**) and potassium isocyanoacetate (**346**) affords the Ugi primary adducts (**347**), which suffer the attack of a second amine molecule to give the unexpected dipeptide derivatives (**348**) in place of the expected ketopiperazines (**349**) product of the Mumm rearrangement (Scheme 67) [110].

**Scheme 68:**
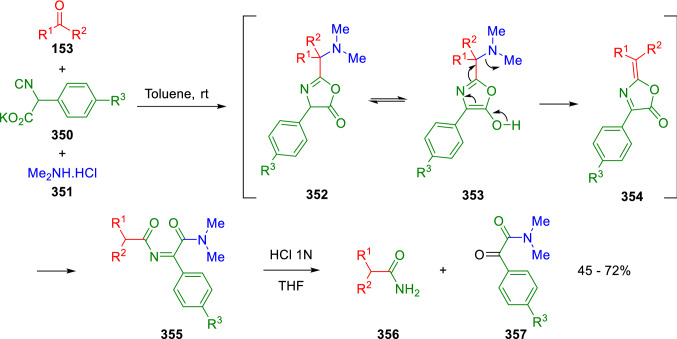
Transformation of oxocompounds to amides

Zhu et al. found that this reaction could be extended to the use of secondary amines and aldehydes or ketones if toluene is used as a solvent [111]. They also found that the presence of ammonium chloride as an additive is crucial for the success of the reaction. Conversely, the reaction of aldehydes or cyclic ketones (**153**) with dimethylamine hydrochloride (**351**) and potassium 2-isocyano-2-arylylacetates (**350**) gave the *N*-acyl imino amides (**355**) as the predominant product (Scheme 68). Subsequent treatment with aqueous acid produces amide (**356**) and ketoamide (**357**). According to the mechanism proposed by the authors, when an aromatic ring is attached to the α-position of isocyanide (**350**), the oxazolone (**352**) exists in the predominant enolic form (**353**) and suffers a 1,6-elimination of dimethylamine to give (**354**). The ensuing nucleophilic attack of dimethylamine results in the ring-opening to yield *N*-acyl imino amide (**355**), which gives by hydrolysis the amide (**356**) and ketoamide (**357**; Scheme 68). In this case, the reaction takes place in toluene at room temperature without ammonium chloride and only 1.2 equivalents of amine are used [112].

## Post-condensation transformations of Passerini and Ugi adducts

Marcaccini performed extensive work on the transformation of Passerini and Ugi adducts. His work makes clear that a well-planned sequence of classical Ugi or Passerini isocyanide multicomponent reactions and post-condensation transformations constitutes an extremely powerful synthetic methodology for the preparation of structurally diverse complex molecules, such as heterocyclic compounds with elaborate substitution patterns, constrained peptides, peptide mimetics, and pseudopeptides. The presence of complementary reactive groups in the multicomponent adducts may facilitate post-condensation transformations that depend on the nature and position of these groups. These reactive groups can easily be incorporated into the product as part of some of the MCR reagents. Generally, protecting group strategies are not necessary, as IMCRs are tolerant to a great diversity of functional groups. Interestingly, some functionalities created in the IMCR can be used in further transformations, meaning that only one extra functional group must be present in one of the starting reagents. Thus, two acidic positions in the Ugi and Passerini adducts –the NH amide group and the peptidyl CH position — can undergo deprotonation, generating new nucleophilic centres able to react intramolecularly with other functional groups.

### Enolisation of the peptidyl hydrogen

**Scheme 69:**
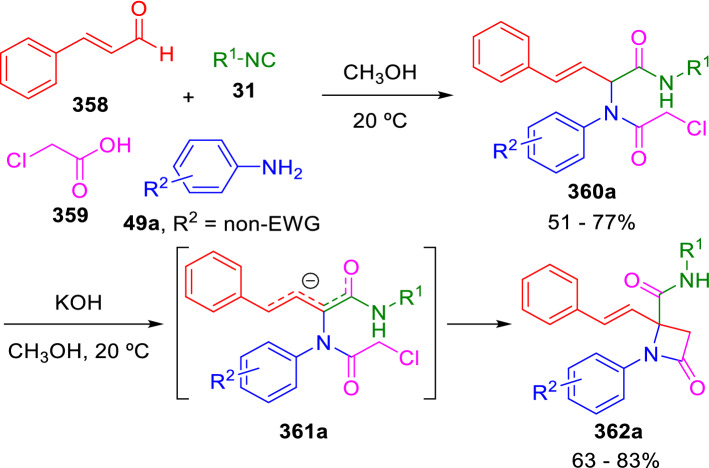
Synthesis of β-lactams from Ugi adducts

In some cases, the peptidyl hydrogen on the asymmetric carbon resulting from a Passerini or an Ugi reaction can be abstracted with a base to generate a nucleophilic carbanion able to react intramolecularly with electrophilic groups in the adduct. However, in most cases, this hydrogen is not acidic enough, despite being adjacent to an electron-withdrawing amide. Marcaccini succeeded in increasing the acidity of the peptide hydrogen by introducing functionalities capable of stabilizing the resulting carbanion. For example, the use of cinnamaldehyde (**358**) as one of the components in a four-component Ugi condensation affords an adduct (**360**) that it is easily deprotonated with moderate bases to give a highly delocalised anion (**361**), containing several reactivity centres. When a complementary electrophilic centre is introduced with one of the components of the U-4CC, such as chloroacetic acid (**359**), the adduct (**360**) can be easily cyclised with KOH in methanol to a *β*-lactam (**362**; Scheme 69) [113]. This is a rare example of β-lactam ring formation via a C_3_–C_4_ bond.

**Scheme 70:**
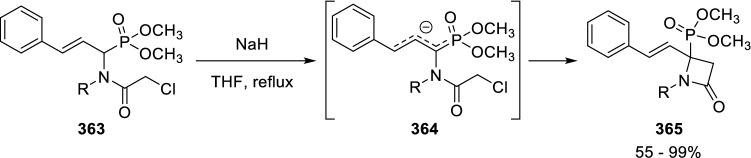
Synthesis of 4-phosphono-β-lactams reported by Stevens

Remarkably, a similar strategy for the synthesis of 4-phosphono-β-lactams (**365**) was later reported by Stevens et al. (Scheme 70) [114, 115].

It is interesting that in both Marcaccini’s and Stevens’ syntheses, cyclisation occurs by attack of the α-carbon rather than the γ-carbon. This seems counterintuitive, as when there is an intramolecular ring-closure competition between a four-and a six-membered ring, the latter is usually preferentially formed. However, in this case, the four-membered ring is selectively formed. Theoretical calculations carried out by Van Speybroeck, Stevens et al. suggest that the four-membered ring preference is due to a geometrically strained *S*_*N*_*2*-like transition state for the six-membered ring formation [116].

**Scheme 71:**
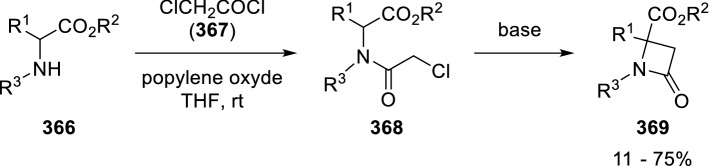
Synthesis of β-lactams from natural amino acids

Another very similar approach to *β*-lactams reported by González-Muñiz starts from amino esters (**366**) that are acylated with chloroacetyl chloride (**367**) to give (**368**) and then cyclised in a basic medium to the corresponding β-lactams (**369**; Scheme 71) [117, 118].

**Scheme 72:**
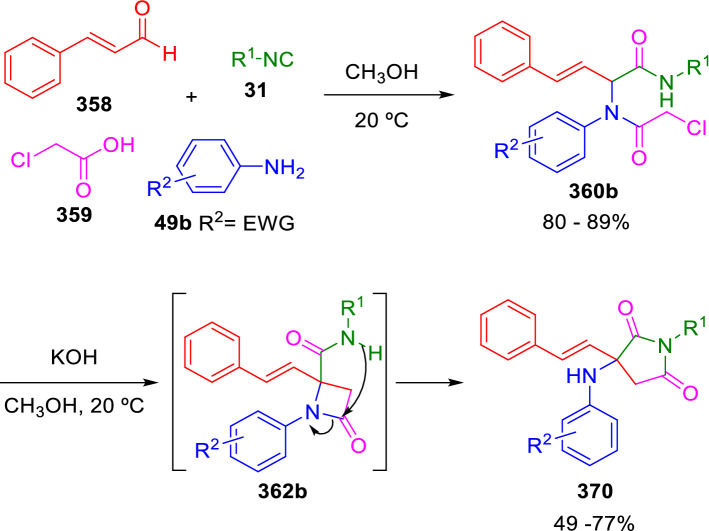
Synthesis of succinimides from Ugi adducts

Interestingly, the β-lactams obtained with Marcaccini’s procedure are stable when the amide nitrogen is substituted with a relatively electron-rich aromatic group resulting from non-substituted anilines or anilines containing electron-donating groups (**49a**; Scheme 69). On the other hand, the use of anilines with strong electron-withdrawing substituents (**49b**) results, after basic treatment of the adducts (**360b**), in β-lactams containing a good leaving nitrogen group (**362b**) that cannot be isolated and are spontaneously transformed to succinimides (**370**) in the reaction conditions (Scheme 72). Thus, the same synthetic procedure gives selective access to two important heterocyclic scaffolds depending on the particular reagents used in each case [119, 120].

**Scheme 73:**
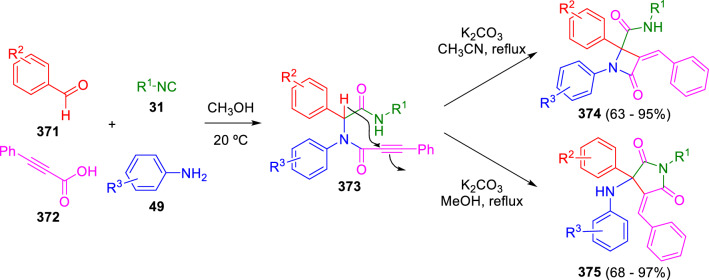
Balalaie’s version of Marcaccini’s synthesis of β-lactams and succinimides

Years later, Balalaie et al. used this same tactic for the synthesis of β-lactams (**374**) and succinimides (**375**) using the Ugi adducts (**373**) of phenylpropiolic acid (**372**). In this case, an aromatic ring is sufficient to stabilise the peptidyl anion, which is generated by K_2_CO_3_. These authors were able to isolate the *β*-lactams (**374**) when the cyclisation is performed in acetonitrile, while the isomerised succinimide products (**375**) are obtained in methanol (Scheme 73) [121]. Very recently, Wang, He and co-workers substituted but-3-ynoic acid derivatives for phenylpropiolic acid, obtaining γ-lactams by U-4CC followed by an intramolecular *5-exo-dig* cyclisation in the presence of Cs_2_CO_3_ [122].

**Scheme 74:**
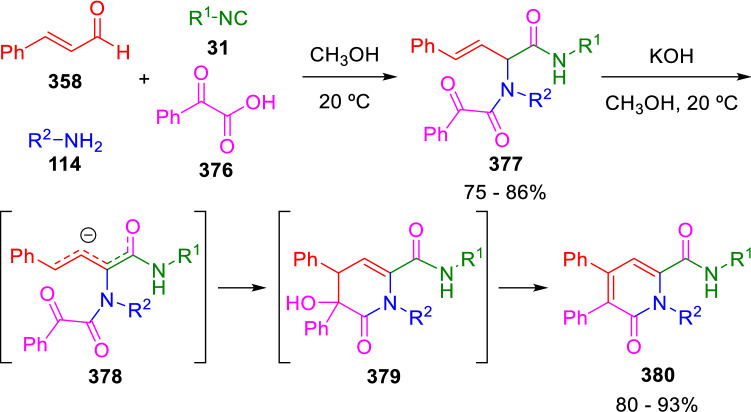
Synthesis of 1,6-dihydro-6-oxopyridines

Moreover, Marcaccini reported that the Ugi adducts of cinnamaldehyde (**358**), glyoxylic acids (**376**), amines (**114**) and isocyanides (**31**) can be easily transformed into oxopyridines (**380**) by basic treatment (Scheme 74) [123]. The key intermediate is again the highly stabilised anion (**378**) which, in this case, behaves as a benzylic anion in the intramolecular nucleophilic attack on the carbonyl group.

**Scheme 75:**
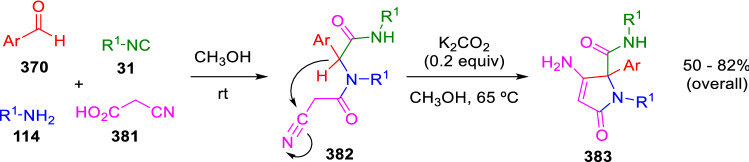
Gómez-Montaño—El Kaïm synthesis of aminopyrrolinones

Here again, a simple aromatic group can also sufficiently stabilise the peptidyl anion, making further cyclisation to heterocyclic products possible. Gómez-Montaño and El Kaïm were able to cyclise in this way the cyanoacetic acid-derived Ugi adducts (**382**), previously prepared by Marcaccini [124–127], to give aminopyrrolinone derivatives (**383**; Scheme 75) [128].

**Scheme 76:**
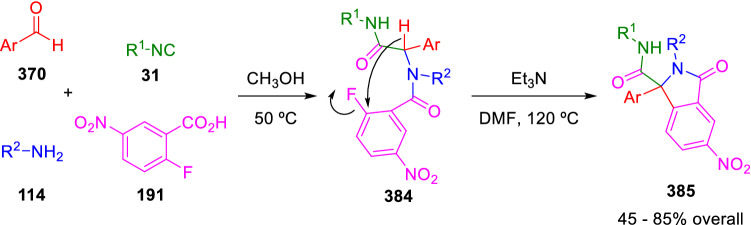
Ivachtchenko’s synthesis of 3-oxoisoindoline-1-carboxamides

Ivachtchenko et al. used the acidity of the Ugi peptidyl hydrogen to promote aromatic nucleophilic reactions leading to isoindole derivatives (**385**). The reaction takes place with triethyl amine in DMF at high temperatures. In this case the anion is stabilised either by a phenylvinyl group, as in the previous Marcaccini’s reactions, or by electron-deficient aromatics introduced as the aldehyde component in the U-4CC (Scheme 76) [129].

Liu et al. used a very similar strategy to prepare 3-(indol-2-yl)isoindolin-1-ones by the cyclisation of the adducts of 1*H*-indole-2-carbaldehyde, 2-iodobenzoic acid, amines and isocyanides. In this case the reaction was carried out in DMSO, under microwaves, at 80 ºC, using Cs_2_CO_3_ as a base [130].

**Scheme 77:**
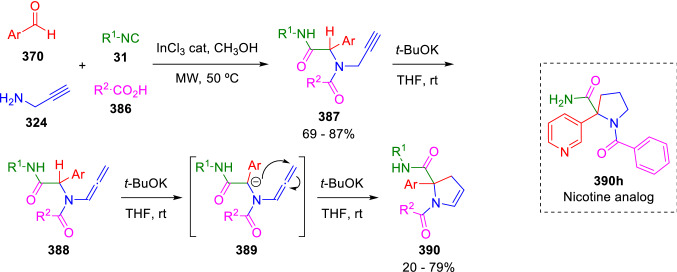
Miranda’s synthesis of 2,3-dihydropyrroles

The possibility of generating a nucleophilic peptidyl anion allows for the synthesis of a variety of scaffolds limited only by the nature of the electrophilic groups present in the Ugi adducts. For example, Miranda synthesised 2,3-dihydropyrroles (**390**) by the cyclisation of peptidyl anions generated from Ugi-allenamide adducts (**388**). The latter in turn are prepared in situ from propargyl amine U-4CC adducts (**387**; Scheme 77) [131]. The utility of this methodology was demonstrated by Vázquez, who used it in conjunction with catalytic hydrogenation of pyrroline products to synthesise a library of nicotine analogues (390h) [132].

**Scheme 78:**
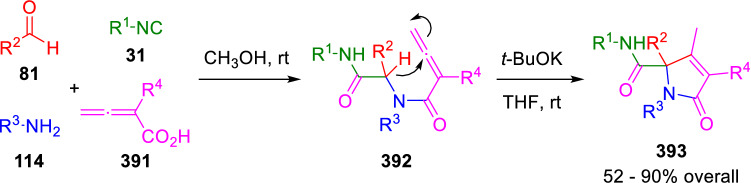
Balalaie’s synthesis of pyrrolidinones

The same strategy was recently applied by Balalaie et al., who cyclised the Ugi adducts (**392**) of allenic acids (**391**) to obtain pyrrolidin-5-one-2-carboxamides (**393**), this time through a 5-*exo*-dig approach (Scheme 78) [133].

**Scheme 79:**
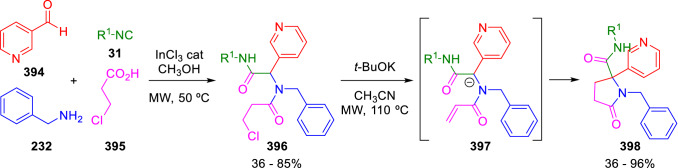
Vázquez’s synthesis of cotinine analogues

A similar method, which starts from 3-chloropropionic acid (**395**) was further developed by Vázquez et al. for the synthesis of cotinine and iso-cotinine analogues (**398**). Here, the direct displacement of the chlorine atom by the attack of the peptidyl anion could explain the formation of the products, but instead evidence supports a two-step base-mediated elimination/Michael addition mechanism (Scheme 79) [134]. A very similar approach to the synthesis of indolyl-substituted lactams by the cyclisation of Ugi-adducts in basic conditions has been also published by Shire et al. [135].

**Scheme 80:**
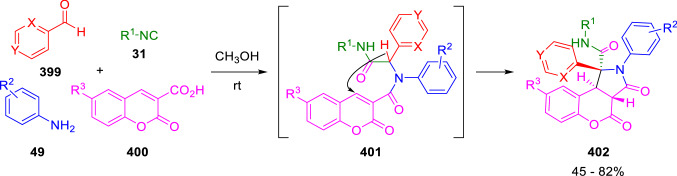
Diastereoselective synthesis of chromeno[3,4-*c*]pyrrole-3,4-diones

An interesting application of the previous reaction was developed by Yang et al. for the diastereoselective synthesis of chromeno[3,4-*c*]pyrrole-3,4-diones (**402**). The carboxylic component of the U-4CC is the *α,β*-unsaturated chromene-3-carboxylic acid (**400**) and the resulting adduct (**401**) spontaneously suffers an intramolecular Michael addition to the expected chromenopyrrole (**402**; Scheme 80) [136].

**Scheme 81:**
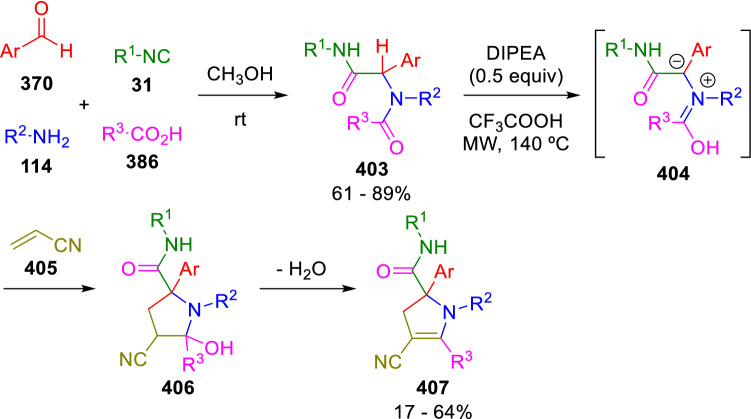
Formal [3 + 2] cycloaddition of Ugi adducts for the synthesis of pyrrolines

The intermolecular addition of Ugi peptidyl carbon to Michael acceptors has also been achieved by Abderrahim and El Kaïm in a two-step synthesis of pyrrolines (**407**). The authors suggest that a concerted [3 + 2] mechanism involving the formation of a dipolar derivative of the Ugi adduct (**404**) occurs (Scheme 81) [137]. Analogously, the peptidyl carbon on Passerini adducts has been shown to act as a nucleophile in Michael additions with acrylonitrile. The resulting γ-hydroxynitrile can be then be cyclised under acidic conditions to yield γ-butyrolactones [138].

**Scheme 82:**
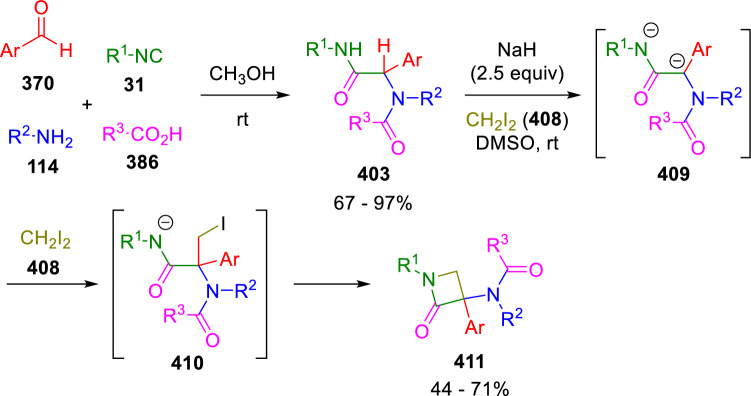
Synthesis of β-lactams by CH_2_I_2_ addition to Ugi adducts

El Kaïm’s group also achieved a simple and general synthesis of β-lactams (**411**) by the addition of diiodomethane (**408**) to amide dianions (**409**) obtained by deprotonation of the Ugi adducts (**403**; Scheme 82) [139]. Calculations suggest that CH_2_I_2_ (**408**) is first added to the peptidyl carbon and the resulting iodomethane-substituted amide (**410**) then cyclises to the desired β-lactam (**411**).

**Scheme 83:**
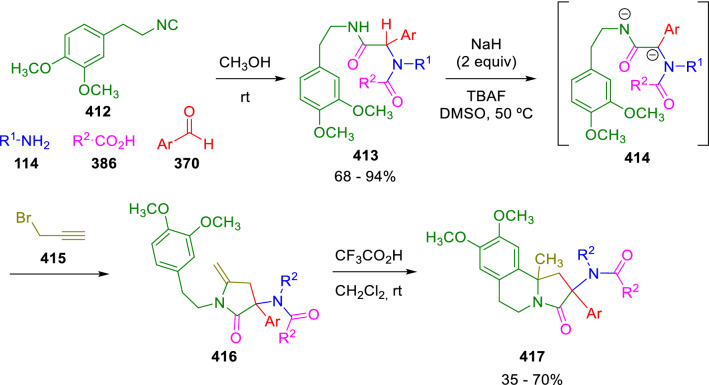
Ugi/propargylation/Pictet−Spengler cyclisation

Analogously, propargyl bromide (**415**) was used as biselectrophile to trap Ugi amide dianions (**414**), resulting in the formation of pyrrolidinone enamides (**416**). This reaction has been combined with a subsequent Pictet−Spengler cyclisation to give benzoindolizine scaffolds (**417**) present in the heterocyclic core of crispine alkaloids (Scheme 83) [140]

**Scheme 84:**
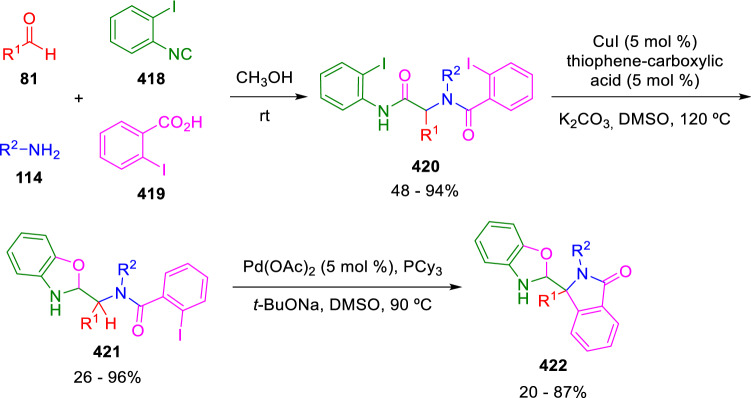
Palladium-catalysed intramolecular C-arylation of the Ugi peptidyl carbon

Other strategies to activate the peptidyl carbon of Ugi adducts have been developed by different groups. As part of their research in the elaboration of Ugi adducts by means of transition metal-catalysed reactions, Neuville and Zhu reported the synthesis of 3-substituted 3-benzoxazolylisoindolinones (**422**) starting from an Ugi-adduct having two aryl iodide units (**420**). The process takes place through a regiospecific sequential intramolecular copper-catalysed *O*-arylation and palladium-catalysed *C*-arylation of the adduct peptidyl position (Scheme 84) [141].

**Scheme 85:**
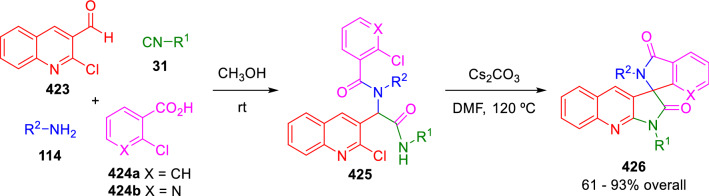
Ghandi synthesis of spiranes

A similar strategy was recently developed by Ghandi et al. for the synthesis of spiropyrroloquinoline isoindolinones and aza-isoindolinones (**426**). In this case, the double cyclisation takes place under metal-free conditions (Scheme 85) [142].

**Scheme 86:**
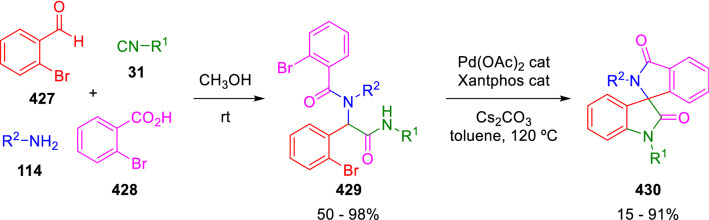
Van der Eycken synthesis of spiranes

A different double cyclisation was proposed by Van der Eycken for the synthesis of spiroindolinone-isoindolinone derivatives (**430**) from Ugi adducts (**229**) in the presence of a palladium catalyst and a base. The authors propose a reaction mechanism involving a first palladium-catalysed Buchwald–Hartwig C-N coupling, followed by a base-promoted addition of the peptidyl carbon to the remaining aryl halide (Scheme 86) [143]. Bromobenzoic acid (**428**) may also be replaced by propiolic acids in the Ugi reaction. In this latter case, spiroindolinone-pyrrolones are formed through a Buchwald–Hartwig/Michael addition sequence [144].

**Scheme 87:**
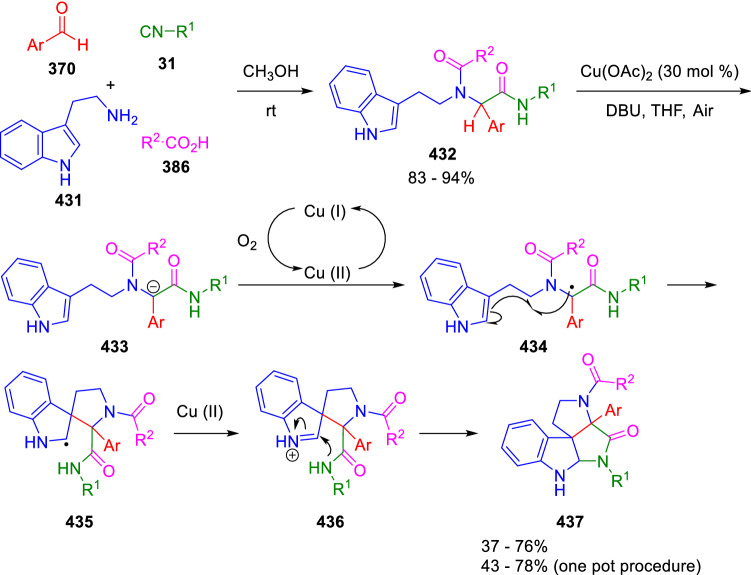
Radical peptidyl activation leading to spiroindolines

On the other hand, El Kaïm and Miranda achieved a copper-catalysed oxidative activation of the peptidyl position of Ugi adducts (**432**) leading to a double radical coupling to efficiently produce complex polycyclic spiroindolines (**437**; Scheme 87) [145].

More recently, Ali and El Kaïm carried out the allylation of Ugi adducts at their peptidyl position using allyl acetate in both the presence and absence of palladium catalysts. This strategy allows the preparation of bis-alkenyl derivatives suitable for subsequent ring-closing metathesis leading to nitrogen heterocycles [146].

**Scheme 88:**
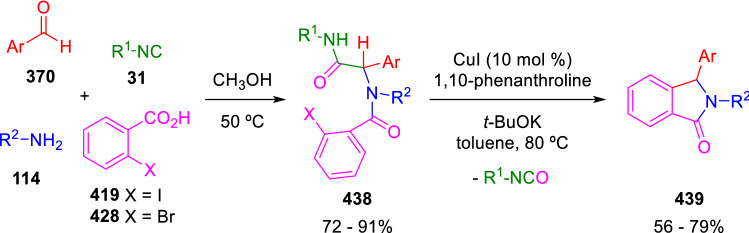
Deamidative C(sp^2^)–C(sp^3^) coupling for the synthesis of isoindolinones

An interesting copper-catalysed intramolecular coupling between the peptidyl carbon and aryl iodide was developed by Chauhan et al., providing a straightforward synthesis of isoindolinones (**439**). The coupling takes place with concomitant loss as isocyanate of the amide moiety originally introduced with the isocyanide component of the U-4CC (Scheme 88) [147]. The mechanism of this reaction is obviously different from the mechanism of Ivachtchenko’s approach to isoindolinones, in which no deamidation is produced (Scheme 76) [129]. Van der Eycken proposed a variant of this reaction using 2-chloronicotinic acid-derived Ugi adducts that cyclise in the presence of a base, with no need of metal catalysis [148].

**Scheme 89:**
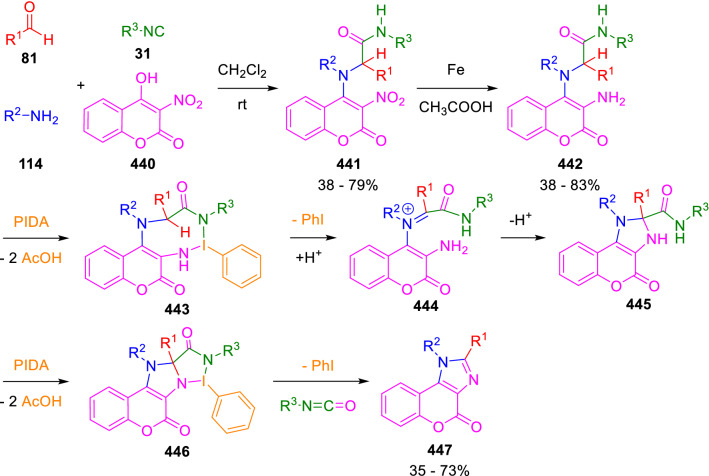
Amide-directed oxidative cyclisation of enol-Ugi derivatives

Marcos et al. have recently developed an oxidative C(sp^3^)–H intramolecular imination of hydroxycoumarin enol-Ugi adduct derivatives (**442**) which leads to imidazolocoumarins (**447**) [149]. Interestingly, the regioselectivity of the reaction is controlled by the amide group derived from the enol-Ugi isocyanide component, which directs the functionalisation of the adjacent C(sp^3^)–H and then is lost as an isocyanate (Scheme 89).

**Scheme 90:**
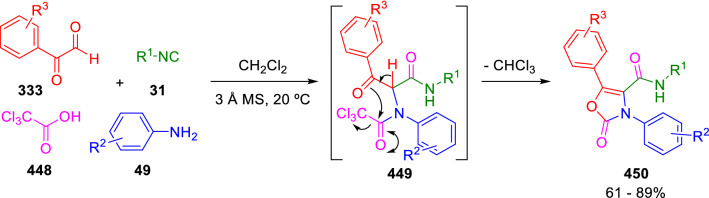
Synthesis of 2(3*H*)-oxazolone 4-carboxamides

In hopes of creating easily enolisable Ugi adducts, Marcaccini introduced the use of arylglyoxals (**333**) as the carbonyl component. The presence of an additional carbonyl group at the enolisable position of the adduct would obviously favour the formation of the corresponding enolate. Thus, the U-4CC of arylglyoxals (**333**), anilines (**49**), isocyanides (**31**) and trichloroacetic acid (**448**) led to highly reactive Ugi adducts (**449**) that spontaneously cyclised in the reaction medium to yield oxazolones (**450**; Scheme 90) [150]. Interestingly, Marcaccini introduced here for the first time the trichloromethyl functionality as a convenient leaving group in post-condensation transformations of IMCRs.

**Scheme 91:**
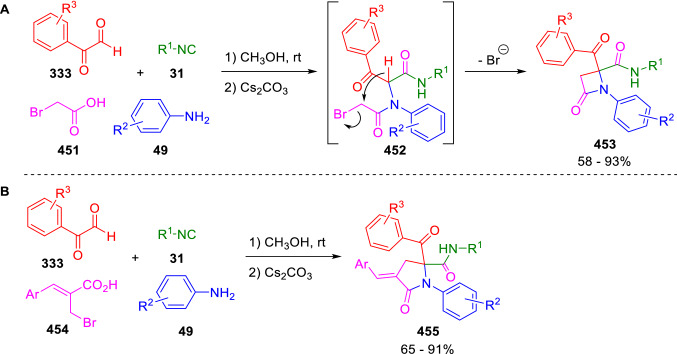
Synthesis of β- and γ-lactams by the Ding’s group

The ready enolisation of the Ugi adducts of arylglyoxals (**452**) was used by Ding in a synthesis of β-lactams (**453**) conceptually related to those published by Marcaccini in 1998 (Scheme 91, A) [119, 151]. In this case, the leaving group is a bromine atom from the bromoacetic acid (**451**) component used in the U-4CC. The use of a 3-bromopropionic acid derivative (**454**) permitted, in turn, the synthesis of the corresponding 5-membered lactams (**455**; Scheme 91, B) [152]. Analogously, the related Passerini reaction led to the corresponding γ-lactone [153].

**Scheme 92:**
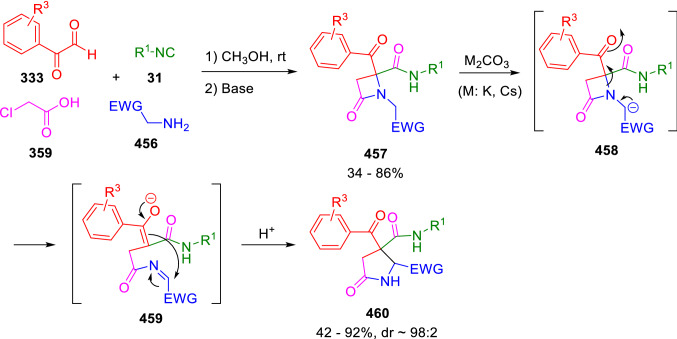
Ring expansion of β-lactams

Interestingly, the β-lactams (**457**) obtained from the Ugi adducts of α-activated amines (**456**), chloroacetic acid (**359**) and arylglyoxals (**333**), are easily transformed to highly functionalised γ-lactams (**460**) by treatment with simple and low-moisture-sensitive bases. The ring expansion is explained by an anionic rearrangement. Thus, the initial deprotonation of the acidic position in the *N*-substituent of the azetidinone (**457**) triggers a ring opening that yields a new intermediate containing an imine and an enolate, which cyclises to the γ-lactams (**460**; Scheme 92) [154].

**Scheme 93:**
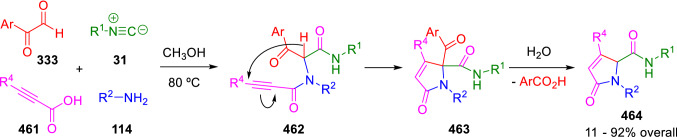
Peshkov and Van der Eycken synthesis of pyrrolone-2-carbox-amides

Peshkov and Van der Eycken proposed the use of arylglyoxals (**333**) and propiolic acids (**461**), as aldehyde and acid components, respectively, to prepare easily enolisable Ugi adducts also containing a Michael acceptor (**462**). The U-4CC, carried out in methanol at 80 ºC, was spontaneously followed by the intramolecular Michael addition of the peptidyl anion to the triple bond to give the corresponding pyrrolones (**463**). A subsequent retro-Claisen fragmentation results in the cleavage of the benzoyl moiety to yield the final pyrrolone-2-carboxamides (**464**; Scheme 93) [155]. Here, in contrast to Balalaie’s work cited above [121], a *5-endo-dig* rather than a *4-exo-dig*-carbocyclisation takes place.

**Scheme 94:**
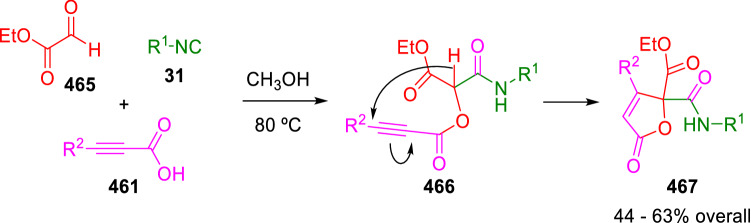
Peshkov and Van der Eycken synthesis of butenolides

In a subsequent work, the same group substituted heterocyclic aldehydes containing basic nitrogen atoms for the glyoxal carbonyl component of the U-4CC, obtaining the expected γ-lactams [156]. Moreover, an analogous Passerini/cycloisomerisation process allowed to conveniently obtain butenolides (**467**) in good yields (Scheme 94) [157].

**Scheme 95:**
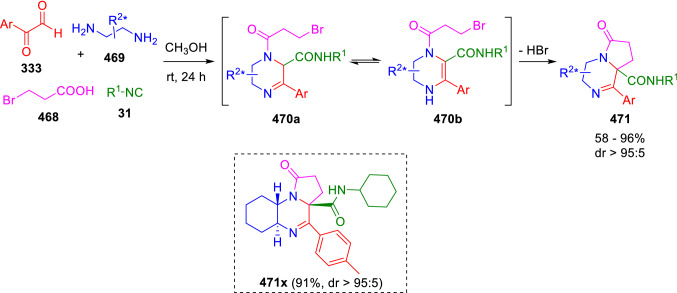
Multicomponent domino reaction in the synthesis of pyrrolopiperazines

Taking advantage of the ready enolisation of the Ugi adducts derived from arylglyoxals as well as their feature as doubly functionalised reactants, García-Valverde et al. have developed a one-pot synthesis of enantiopure pyrrolopiperazines (**471**) through a diastereoselective multicomponent domino reaction, Ugi/enamine alkylation, using non-protected 1,2-diamines (**469**) together to arylglyoxals (**333**) and 3-bromopropionic acid (**468**). Thus, after the formation of a cyclic diamine, the Ugi reaction takes place selectively over the aldimine position. The spontaneous alkylation of the cyclic enamine intermediate (**470**) affords the corresponding pyrrolopiperazine (**471**), with the generation of a new stereogenic centre in the last step (Scheme 95) [158].

### Amide NH as nucleophile

**Scheme 96:**
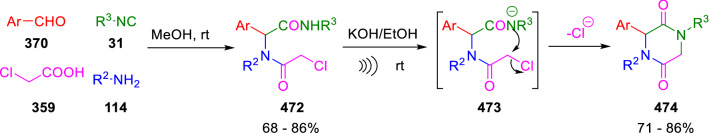
Synthesis of 2,5-diketopiperazines

The transformations shown above rely on the formation of a highly stabilised anion in the peptidyl position. This occurs when the appropriate substituents are introduced on Passerini or Ugi adducts with the initial reaction components. However, when the aldehyde employed as starting reactant is unable to stabilise the peptidyl anion, or this anion is not in the correct position to react with other groups in the molecule, a base-induced transformation involving the amide nitrogen may take place. An interesting example reported by Marcaccini is the basic treatment under ultrasonic sonication of the Ugi adduct (**472**) of aromatic aldehydes (**370**) and chloroacetic acid (**359**), which affords 2,5-diketopiperazines (**474**) as a result of an intramolecular *N-*alkylation (Scheme 96) [159].

**Scheme 97:**
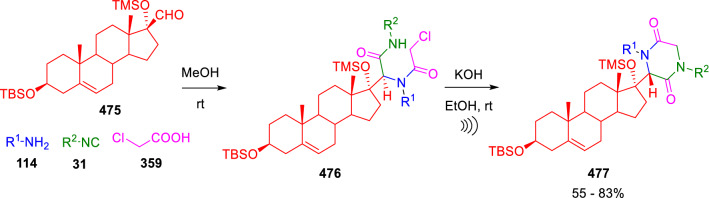
Diastereoselective synthesis of 2,5-diketopiperazine-derived steroids

Marcaccini described the limitations of this methodology. On one hand, the cyclisation was unsuccessful with aliphatic aldehydes and, on the other, epimerisation of the stereogenic centre occurs in the cyclisation conditions, as evidenced by the incorporation of deuterium at the C3 position when KOD in EtOD was employed. However, when the aldehyde group was directly linked to a steroidal framework (**475**), these drawbacks were not observed and, despite the aliphatic characteristic of the aldehyde employed, the reaction took place in good yield. Moreover, high diastereoselectivity was achieved in the Ugi reaction, possibly induced by the rigid steroidal frame, and mantained during the cyclisation (Scheme 97) [160].

**Scheme 98:**
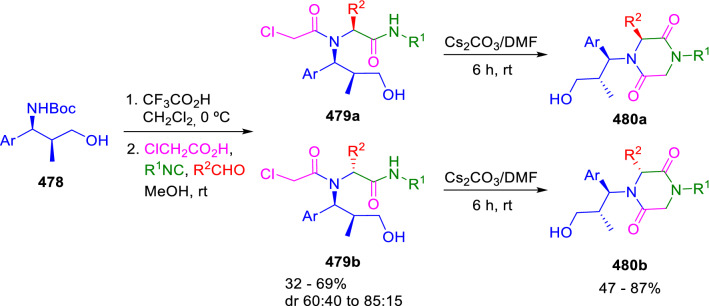
Synthesis of homochiral 2,5-diketopiperazines

Interestingly, Banfi et al. described a similar strategy for the synthesis of 2,5-diketopiperazines (**480a** and **480b**) from chiral amino alcohols (**478**) using caesium carbonate instead of potassium hydroxide in the final stage. The cyclisation was carried out on the separated diastereomers of the Ugi adducts (**479a** and **479b**), as under these reaction conditions the epimerisation on the C3 position of the diketopiperazines was not observed (Scheme 98) [161].

**Fig. 2:**
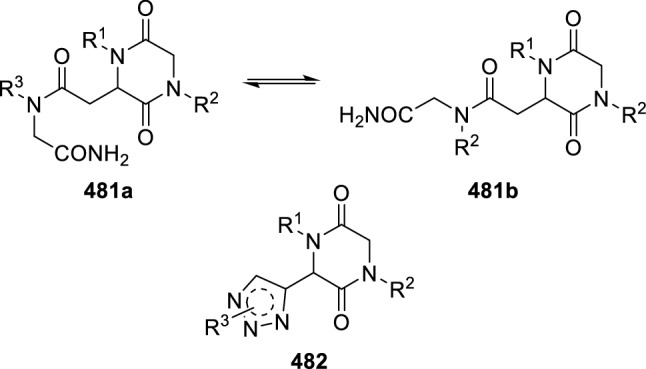
2,5-Diketopiperazine inhibitors of Apaf-1

Messeguer et al. employed this methodology in the design of analogues of linear *N*-alkylglycine oligomers (**481**), reported as apoptotic inhibitors [162]. These authors created cyclic motifs in these peptoids to introduce geometric constraints that reduce conformational freedom and increase their selectivity. Moreover, to avoid rotamers, they substituted an isostere triazole moiety for the tertiary amine (**482**; Fig. 2). The resulting peptoids were shown to be Apaf-1 inhibitors that decrease the apoptotic phenotype in mitochondrial-mediated models of cellular apoptosis.

**Scheme 99:**
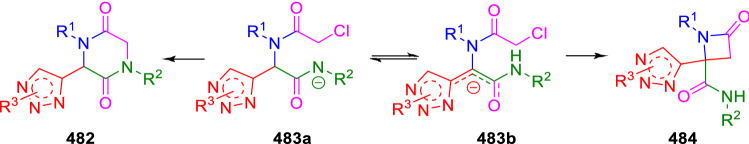
Divergent cyclisation of chloroacetic acid derived Ugi adducts

They carried out the synthesis of these restricted analogues (**482**) through an Ugi reaction combining chloroacetic acid (**359**) and triazole aldehydes, followed by a base-induced intramolecular cyclisation, which afforded, not only the expected 2,5-diketopiperazine (**482**), but also the corresponding β-lactam (**484**), because of the increased acidity on the peptidyl position (Scheme 99) [163]. The influence of the substitution on the secondary amide and the triazole core was determinant for the experimental results and was theoretically studied [164, 165]. Interestingly, the peptidomimetics bearing the β-lactam scaffold (**484**) turned out to be more potent apoptotic inhibitors than the diketopiperazine isomers.

**Scheme 100:**
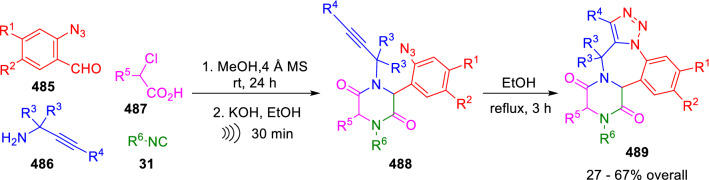
Synthesis of polycyclic diketopiperazines reported by Dehaen

Additionally, diketopiperazines fused with other heterocycles have been synthesised by Dehaen et al. using this methodology, combining chloroacetic acid with other functionalised reactants in the U-4CC. Thus, triazolobenzodiazepine diketopiperazines (**489**) were synthesised from 2-azidobenzaldehydes (**485**), propargyl amines (**486**), chloroacetic acid derivatives (**487**) and isocyanides (**31**). The Ugi adduct was cyclised under basic conditions to the diketopiperazine system bearing azide and alkyne functionalities (**488**), which was then subjected to intramolecular azide-alkyne cycloaddition (IAAC) in refluxing ethanol to give the fused system (**489**; Scheme 100) [166].

**Scheme 101:**
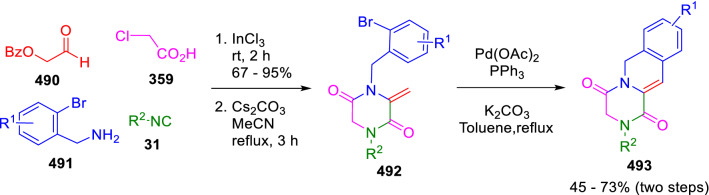
Synthesis of pyrazinoisoquinolines

Miranda used 2-bromobenzylamines (**491**) and benzoylacetaldehyde (**490**) as doubly functionalised reactants along with chloroacetic acid (**359**) in the synthesis of pyrazinoisoquinolines (**493**). The fused systems were obtained through a three-step protocol consisting of an Ugi reaction followed by a basic medium-promoted cyclisation/elimination and a 6-*endo* Heck cyclisation (Scheme 101) [167].

**Scheme 102:**
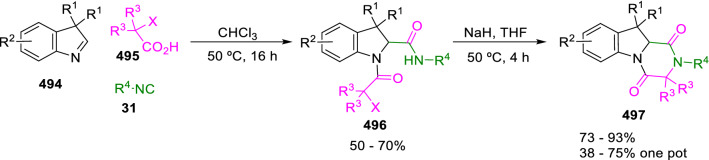
Synthesis of tetrahydropyrazino[1,2-*a*]indole-1,4-diones

The synthesis of tetrahydropyrazino[1,2-*a*]indole-1,4-diones (**497**) by the Joullié-Ugi reaction of indolenines (**494**), haloacetic acids (**495**) and isocyanides (**31**), followed by an intramolecular *N-*alkylation, was reported by Krasavin (Scheme 102) [168]. The starting indolenines (**494**) can be, in turn, easily obtained from phenylhydrazines and 2,2-dialkyl acetaldehydes following a Fischer protocol.

**Scheme 103:**
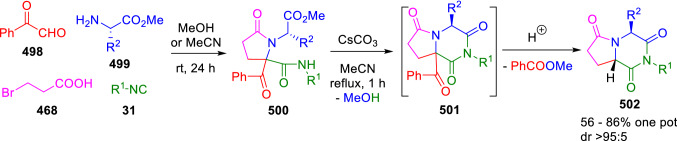
Synthesis of pyrrilopiperazine-2,6-diones

Following a one-pot two-step methodology, García-Valverde et al. described the diastereoselective synthesis of pyrrolopiperazine-2,6-diones (**502**). The synthesis is carried out through an Ugi/nucleophilic substitution/*N-*acylation/debenzoylation sequence using three doubly functionalised reactants –phenylglyoxal (**498**), α-amino esters (**499**) and 3-bromopropionic acid (**468**)- along with the isocyanide (**31**; Scheme 103) [169].

**Scheme 104:**
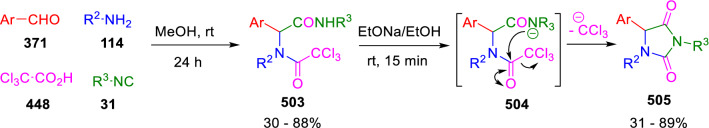
Marcaccini’s hydantoin synthesis

Furthering the studies on the cyclisations of Ugi adducts promoted by the deprotonation of the amide nitrogen, Marcaccini's group proposed the synthesis of Ugi adducts with a highly electrophilic α-acylamino substituent that would facilitate the intramolecular nucleophilic attack at this position. Therefore, they chose trichloroacetic acid (**448**) as one of the components of the U-4CC to facilitate the ring closure through an *N-*acylation leading to hydantoins (**505**). This synthesis is based on the enhanced electrophilic character of the trichloroacetamide group and the quality of trichloromethyl anion as leaving group, allowing the trichloroacetyl group to function as a masked carbonic acid surrogate. In this way, the Ugi adducts (**503**) synthesised from primary amines (**114**), aryl aldehydes (**371**), trichloroacetic acid (**448**) and isocyanides (**31**), upon treatment with sodium ethoxide, underwent a rapid ring-closure reaction to give hydantoins (**505**) in good yields. Moreover, the products were easily isolated from the reaction medium by precipitation from the mother liquors (Scheme 104) [170].

**Scheme 105:**
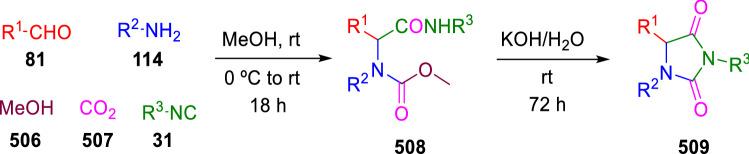
Hulme’s hydantoin synthesis

This synthesis constitutes a more efficient methodology than the route described by Hulme’s group for the synthesis of hydantoins (**509**) via an Ugi five-component condensation followed by a base-promoted cyclisation (Scheme 105) [171].

**Scheme 106:**
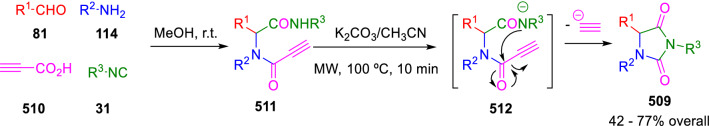
Chen’s synthesis of hydantoins

A similar strategy replacing trichloroacetic acid by propiolic acid (**510**) has been reported by Chen et al. Surprisingly the base treatment of the Ugi adduct (**511**) affords the corresponding hydantoin (**509**), resulting from the behaviour of the acetylide anion as the leaving group. In contrast, the expected intramolecular Michael addition that would give rise to a diazepine did not occur (Scheme 106) [172].

**Scheme 107:**
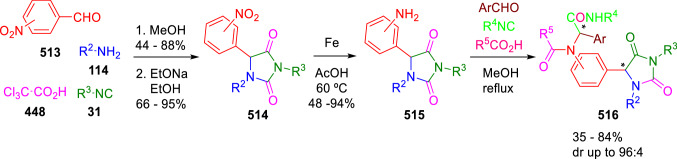
Diastereoselective synthesis of pseudopeptidic hydantoins

The methodology described by Marcaccini´s group has been employed for the synthesis of pseudopeptidic hydantoins (**516**) using Ugi/cyclisation/reduction/Ugi sequences, by inclusion of a nitro group as amine surrogate in the carbonyl component (**513**). Interestingly, the pseudopeptidic hydantoins (**516**) were obtained in many cases with a high diastereoselectivity (Scheme 107) [173].

**Scheme 108:**
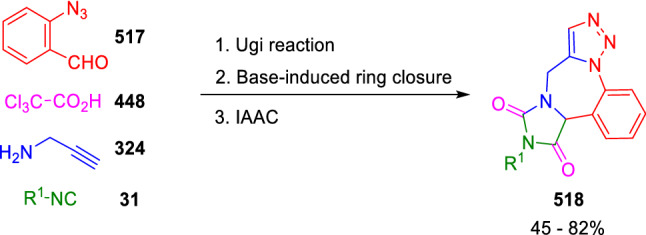
Dehaen tandem synthesis of hydantoin-fused benzodiazepines

This strategy has also been used for the synthesis of fused heterocycles, following two different approaches. The first approach starts with the synthesis of the hydantoin scaffold with the proper functionalisation for the subsequent synthesis of the corresponding fused heterocycle. This methodology was chosen by Dehaen et al. for the synthesis of hydantoin-fused triazolobenzodiazepines (**518**), using 2-azidobenzaldehyde (**517**), propargylamine (**324**), trichloroacetic acid (**448**) and isocyanides (**31**) as starting materials for the Ugi reaction. The treatment of the Ugi adducts with sodium ethoxide afforded the functionalised hydantoin intermediates, which, when subjected to intramolecular azide-alkyne cycloaddition (IAAC), afforded the expected hydantoin-fused benzodiazepine derivatives (**518**; Scheme 108) [166].

**Scheme 109:**
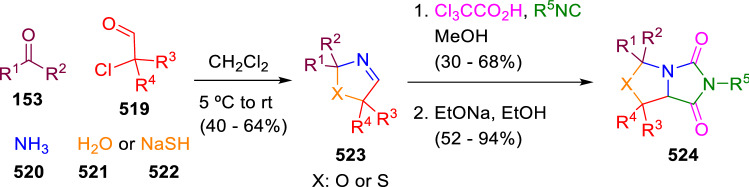
Asinger/Ugi-Joullié reaction for the synthesis of oxa(thia)zolidine-fused hydantoins

**Scheme 110:**
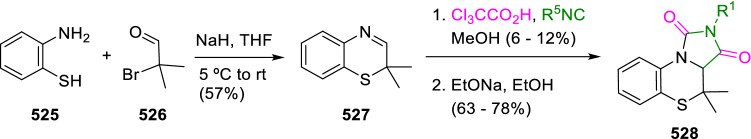
Synthesis of benzothiazine-fused hydantoins

The second approach starts with the synthesis of cyclic imines followed by an Ugi-Joullié reaction, which introduces the trichloroacetic acid (**448**) for the base-induced cyclisation to the hydantoin-fused system. In this manner, Martens et al. described the synthesis of two different families of fused heterocycles: hydantoins fused with oxa(thia)zolidines (**524**) and 1,4-benzothiazines (**528**). The methodology developed for the synthesis of oxa(thia)zolidine-fused hydantoins (**524**) combines two multicomponent reactions, an Asinger and an Ugi-Joullié reaction (Scheme 109), whilst the synthesis of the second family (**528**) began with the condensation of 2-bromo-2-methylpropionaldehyde (**526**) and 2-aminothiophenol (**525**) affording the 2*H*-1,4-benzothiazines (**527**) required for the ulterior Ugi-Joullié reaction (Scheme 110) [174].

**Scheme 111:**
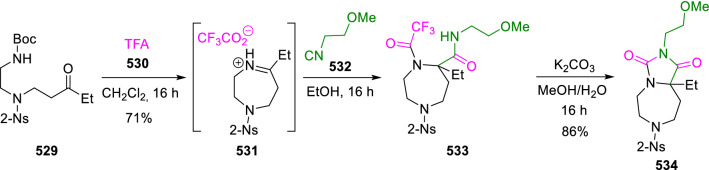
Synthesis of diazepine-fused hydantoins

Hydantoins fused with piperazines and diazepines have also been synthesised following this strategy. Thus, Nelson et al. used an *N-*Boc deprotection/Ugi-Jouillé condensation/cyclisation sequence. In this sequence the trifluoracetic acid (**530**) used as reagent in the deprotection of the amine group plays a double role, acting also as the acid component in the Ugi-Joullié reaction, and finally as a carbonic acid surrogate in the last stage of the synthesis (Scheme 111) [175].

**Scheme 112:**
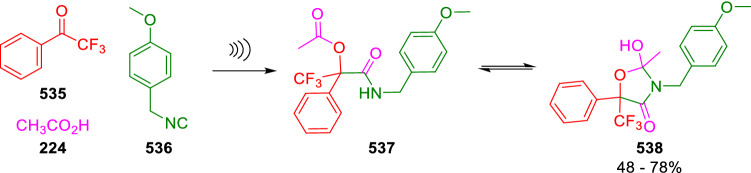
Cyclisation of Passerini adducts affording oxazole-derived orthoamides

Conversely, the intramolecular NH attack on Passerini three-component adducts (**537**) of trifluoromethyketones (**535**) gave rare orthoamides (**538**), the result being highly dependent on the nature of the isocyanide, as well as the temperature and catalyst charge (Scheme 112) [176]. These pentacyclic orthoamides (**538**) are structurally related to those obtained by Marcaccini (**307**) by the cyclisation of P-3C-2C adducts of 6-oxo-4-thiacarboxylic acids through a similar mechanism (Scheme 56) [92].

**Scheme 113:**
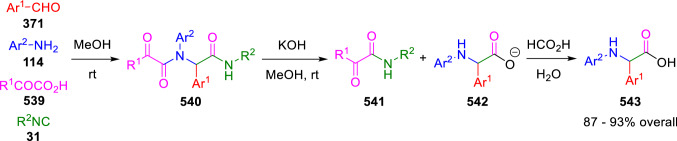
Synthesis of 2,*N*-diarylglycines by the cleavage of Ugi adducts of glyoxylic acids

A similar intermediate was proposed by Marcaccini et al. in the synthesis of 2,*N*-diarylglycines (**543**) from the basic treatment of Ugi adducts (**540**) synthesised from aromatic aldehydes (**371**), anilines (**114**), α-ketoacids (**539**) and isocyanides (**31**; Scheme 113) [177].

**Scheme 114:**
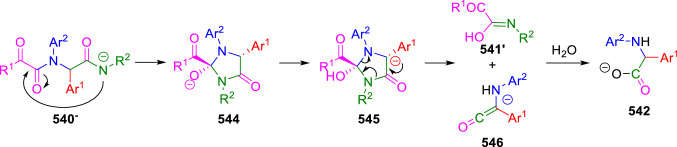
Mechanism for the cleavage of Ugi adducts leading to 2,*N*-diarylglycines

These chemical results were not achieved when aliphatic aldehydes or amines were used or when aldehydes were replaced by ketones. Based on these experimental results, the formation of the arylglycine derivatives was explained by the base-induced formation of imidazolidinone intermediates (**544**), which would undergo the deprotonation at C5 position. This key deprotonation step is not possible when ketones, aliphatic amines and/or aldehydes were employed in the Ugi condensation, since, either there is no hydrogen at the C5 position, or this is not acidic enough. Rearrangement of anion **545** would then lead to the intermediate ketene anions (**546**), which would give directly the α-amino acid salts (**542**) via the addition of water or methanol, followed by the hydrolysis of the corresponding methyl esters (Scheme 114).

### Amine NH as nucleophile on adducts of the hydrazoic acid variant of the Ugi reaction

A variant of the classic Ugi reaction uses hydrazoic acid in place of the usual carboxylic acids to obtain α-amino tetrazoles. Marcaccini took advantage of the nucleophilic character of the α-amino group on the adducts to design post-condensation transformations introducing the appropriate complementary electrophilic groups with one of the components of the U-4CC.

**Scheme 115:**
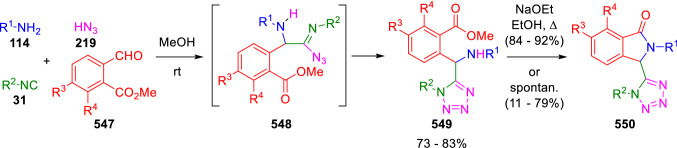
Synthesis of tetrazoloisoindolines by post-condensation of azide-Ugi adducts

Thus, the Ugi condensation involving methyl *o*-formylbenzoates (**547**), amines (**114**), isocyanides (**31**), and hydrogen azide (**219**; prepared in situ from trimethylsilylazide and MeOH or from dimethylamine hydrochloride and sodium azide) gives a primary adduct (**548**) that undergoes a spontaneous 1,5-dipolar cyclisation to give the α-amino tetrazole (**549**). Next, nucleophilic cyclisation affords isoindolinones (**550**). This latter reaction takes place spontaneously when benzyl or alkyl amines are used, and requires basic conditions with aromatic amines (Scheme 115) [178].

**Scheme 116:**
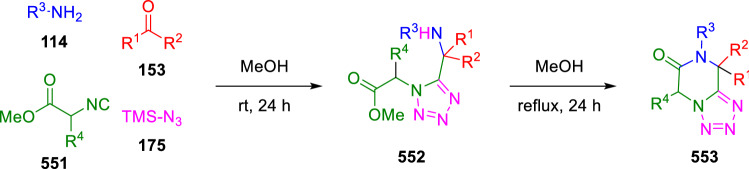
Synthesis of ketopiperazinetetrazoles by post-condensation of azide-Ugi adducts

Hulme used a similar strategy, in which the ester moiety necessary for the post-condensation amidation is localised in the isocyanide (**551**). This reacts with carbonyl compounds (**153**), amines (**114**) and trimethylsilylazide (**175**) to give α-amino tetrazoles (**552**)**,** which are heated in methanol to give fused ketopiperazinetetrazoles (**553**, Scheme 116) [179].

**Scheme 117:**
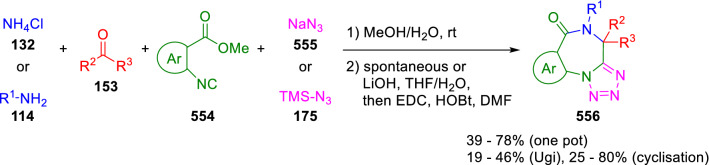
Synthesis of tetrazolobenzodiazepines by post-condensation of azide-Ugi adducts

Analogous tetrazolobenzodiazepines (**556**) were likewise synthesised by Voskressensky in a one-pot U-4CC of ketones (**153**), ammonium chloride (**132**), bifunctional isocyanides (**554**), and sodium azide (**555**) or trimethylsilylazide (**175**), followed by cyclisation (Scheme 117) [180]. Dömling used the same strategy, substituting primary amines (**114**) for ammonium chloride (**132**) [181]. In this case, ester hydrolysis followed by EDAC/HOBt mediated amide bond formation was required to obtain the final tetrazolobenzodiazepines (**556**; Scheme 117).

**Scheme 118:**
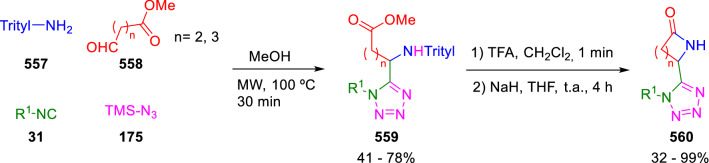
Synthesis of tetrazolo lactams

Recently, Dömling prepared unsubstituted tetrazolo γ- and δ-lactams (**560**) by the Ugi reaction of aliphatic ester-substituted aldehydes (**558**), trityl amine (**557**), isocyanides (**31**) and trimethylsilylazide (**175**), followed by deprotection with TFA and cyclisation with sodium hydride (Scheme 118) [182].

## Additional nucleophilic groups

### Nitrogen nucleophiles

**Scheme 119:**
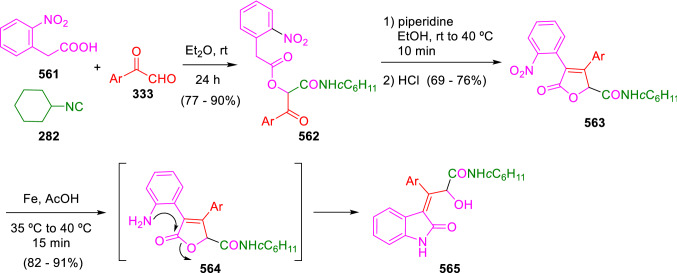
Marcaccini’s three-step synthesis of 2-oxoindoles

Nucleophilic groups present in the components of the Ugi and Passerini reactions may interfere with the condensations, leading to unexpected reaction pathways (See, for example, the reaction between 2-hydroxybenzaldehyde (**700**), isocyanides (**31**) and ammonium formate (**121**; Scheme 147) [183]. To avoid these competitive reactions, different strategies have been developed that use masked internal nucleophiles, such as *N*-protected amines or nitro or azide groups as amine surrogates. If these masked groups are properly located relative to complementary electrophilic sites, intramolecular reactions take place upon their activation, affording a variety of often pharmacologically relevant scaffolds. Thus, while Hulme et al. developed the UDC concept (Ugi reaction/Deprotection/Condensation) [184, 185], Marcaccini’s group pioneered the use of nitro and azide groups as amine surrogates in Ugi/Condensation sequences. Thus, in a seminal work where the nitro group was introduced as a masked amino nucleophile in an IMCR, Marcaccini developed an elegant synthesis of furan derivatives (**563**) through Passerini/Knoevenagel sequences. When 2-nitrophenyl acetic acid (**561**) was chosen as the carboxylic component in a P-3CC with glyoxals (**333**) and isocyanides (**282**), the nitro-group played a double role, increasing the acidity of the benzylic position on the Passerini adduct to favour the intramolecular Knoevenagel condensation in mild conditions and acting as a masked amino-group. In this way, 2,5-dihydro-2-(2-nitrophenyl)-5-oxofuranes (**563**) were obtained upon treatment of the Passerini adducts (**562**) with piperidine and additional treatment with acid. Then, the reduction of the nitro-group afforded 2-oxoindoles (**565**) as a result of the nucleophilic attack of the amine on the lactone carbonyl group with subsequent opening of the lactone ring (Scheme 119) [186]. Thus, the overall reaction pathway involves a first intramolecular reaction with a carbon nucleophile, followed by a ring-switching transformation of a furan into an indole triggered by the attack of the initially masked nucleophile.

**Scheme 120:**
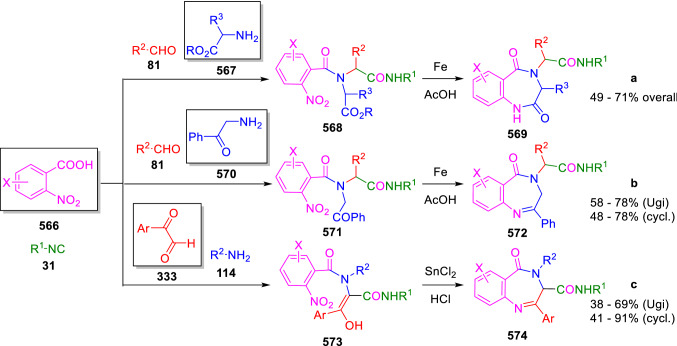
Marcaccini’s synthesis of benzodiazepines by Ugi/reduction/cyclisation sequences

Later, Marcaccini´s group described the synthesis of benzo[1,4]diazepine systems with different substitution patterns (**569**, **572**, **574**) through two-step Ugi/reduction/cyclisation (URC) sequences, varying the nature of the nitro derivative and/or the doubly functionalised reactant with the electrophilic site required for the cyclisation step. Thus, the combination of 2-nitrobenzoic acid (**566**) with α-amino esters (**567**) led to 1,4-benzodiazepine-2,5-diones (**569**; Scheme 120a) [187], while its combination with phenacylamine (**570**; Scheme 120b) [188] or arylglyoxals (**333**; Scheme 120c) [189] afforded stable 4,5-dihydro-3*H-*benzo[1,4]diazepin-5-ones (**572**, **574**). However, when arylglyoxals (**333**) were combined with 2-nitrobenzylamine (**583**), unstable 4-benzoyl-4,5-dihydro-3*H*-benzo[*e*][1,4]diazepines were obtained.

**Scheme 121:**
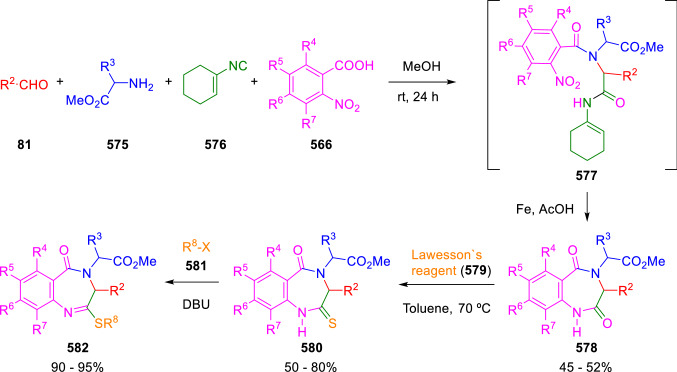
Synthesis of thiobenzodiazepines reported by Guo

Guo used a similar strategy to prepare thiobenzodiazepines (**582**) to test their antitumor activity as p53-MDM2 protein–protein interaction inhibitors. In this case, the use of convertible isocyanide (**576**) permits the obtention of benzodiazepine (**578**) by reduction of the nitro group on the non-isolated Ugi adduct (**577**). The treatment of **578** with Lawesson’s reagent (**579**) affords compounds **580**, which can be further alkylated in the presence of DBU to give **582** (Scheme 121) [190]

**Scheme 122:**
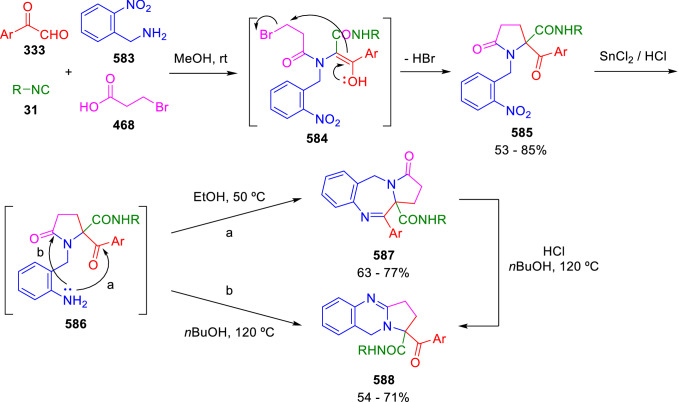
Three-step synthesis of pyrrolobenzodiazepines or pyrroloquinazolines developed by García-Valverde

Based on these results, García-Valverde et al. introduced new functionalised reagents to synthesise more complex systems. In this way, pyrrolobenzodiazepine and pyrroloquinazoline scaffolds have been synthesised from arylglyoxals (**333**) and amino or carboxylic reagents where the nitro group was attached [191, 192]. The reaction of glyoxals (**333**), 2-nitrobenzylamine (**583**), isocyanides (**31**) and 3-bromopropionic acid (**468**) and subsequent intramolecular nucleophilic attack of the peptidyl position onto the alkyl bromide leads to pyrrolidinones (**585**). Reduction with SnCl_2_ unmasks the latent aromatic amine, resulting in pyrrolobenzodiazepines (**587**) or pyrroloquinazolines (**588**), depending on the reaction conditions (Scheme 122) [191].

**Scheme 123:**
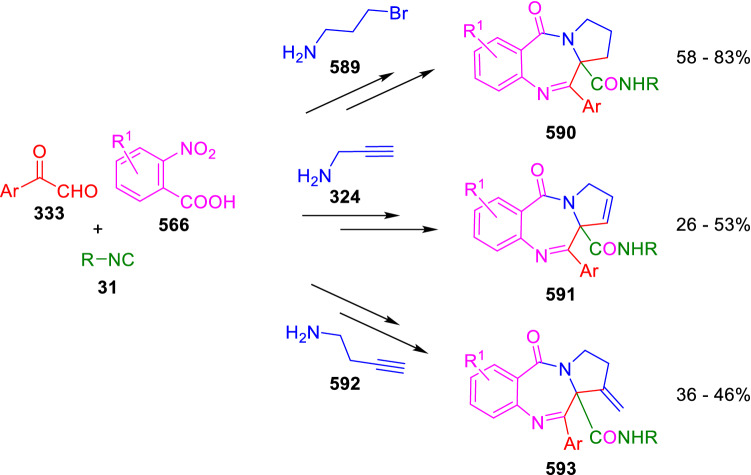
Post-Ugi transformations to diverse pyrrolobenzodiazepines

Similarly, the same authors were able to synthesise pyrrolobenzodiazepines with different degrees of unsaturation (**590**, **591**, **593**) by the post-condensation transformation of arylglyoxals (**333**), isocyanides (**31**), 2-nitrobenzoic acid (**566**) and bifunctional amines (**589**, **324**, **592**; Scheme 123) [192].

**Scheme 124:**
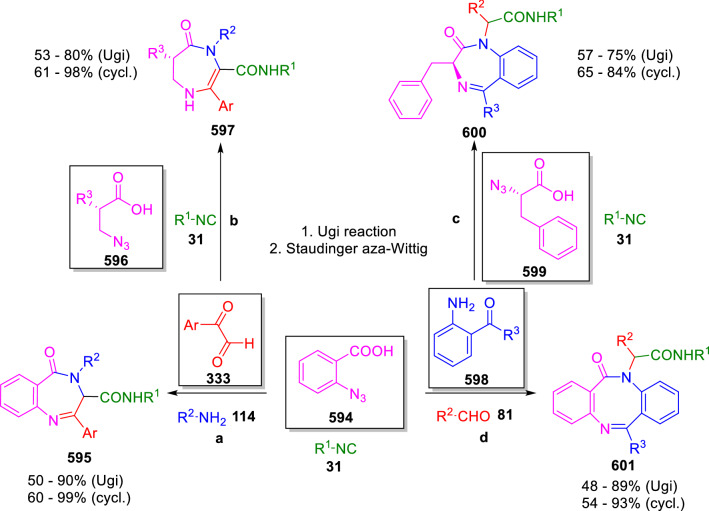
Synthesis of 7- and 8-membered nitrogen heterocycles by Ugi/Staudinger/aza-Wittig cyclisation

Using a complementary approach, Marcaccini synthesised some analogous structures, as well as other medium size nitrogen heterocycles, from azide derivatives as amine surrogates, following Ugi/Staudinger/aza-Wittig cyclisation sequences. Thus, seven-membered rings as 4,5-dihydro-3*H-*benzo[1,4]diazepin-5-ones (**595**) were obtained from 2-azidobenzoic acid (**594**) and arylglyoxals (**333**) (Scheme 124, a) [193]; 5-oxo-4,5,6,7-tetrahydro-1*H*-1,4-diazepines (**597**) from 3-azidopropionic acids (**596**) and arylglyoxals (**333**; Scheme 124, b), and 2-oxo-1,4-benzodiazepines (**600**) from 3-phenyl-2-azidopropionic acid (**599**) and 2-aminobenzophenone (**598**; Scheme 124), c) [194]. Likewise, eight-membered rings as [(5*H*)-6-oxodibenzo[*b,f*][1,5]diazocines (**601**) were synthesised from 2-azidobenzoic acid (**594**) and 2-aroylanilines (**598**; Scheme 124, d) [195].

The stereochemical outcome of these reactions have also been analysed, revealing uneven results. When the chiral information was incorporated into the acid component [194] the diastereoselectivity achieved was extremely poor, however high diastereoselectivities were observed when the chiral information was introduced in the amino component [187]. Moreover, reversal of diastereoselectivity was observed in the synthesis of 3-carboxamide-1,4-benzodiazepin-5-ones depending on the synthetic methodology employed [196].

**Scheme 125:**
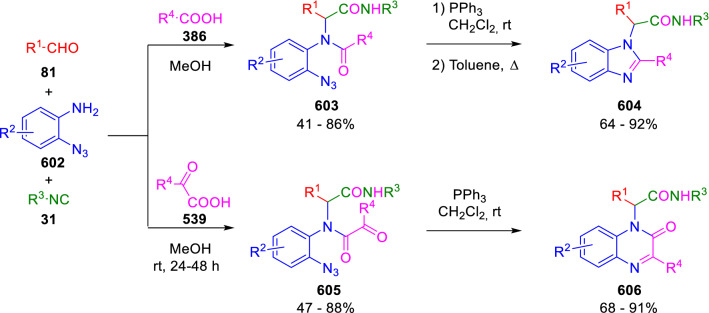
Amino-azides in the Ugi- Staudinger-aza-Wittig process

Yan used aromatic amines linked to an azide group (**602**) to prepare benzimidazoles (**604**) or quinoxalin-2(1*H*)-ones (**606**) using an Ugi/Staudinger/aza-Wittig sequence. When carboxylic acids (**386**) were used in the Ugi reaction, the Ugi-adducts (**603**) reacted with triphenylphosphine at room temperature and successive heating in toluene to give benzimidazoles (**604**). In turn, when glyoxylic acids (**539**) were used, quinoxalin-2(1*H*)-ones (**606**) were formed at room temperature (Scheme 125) [197].

**Scheme 126:**
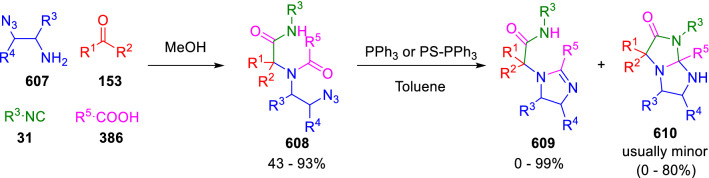
Ugi-Staudinger-aza-Wittig synthesis of imidazolines reported by Wessjohann

Wessjohann also employed the Ugi-Staudinger-aza-Wittig sequence to prepare imidazolines (**609**). Here, the Ugi adducts (**608**) of azidoalkylamines (**607**) were treated with triphenylphosphine or resin bound triphenylphosphine give the imidazolines (**609**). In some cases, *o*-amidines (**610**) were also observed (Scheme 126) [198].

**Scheme 127:**
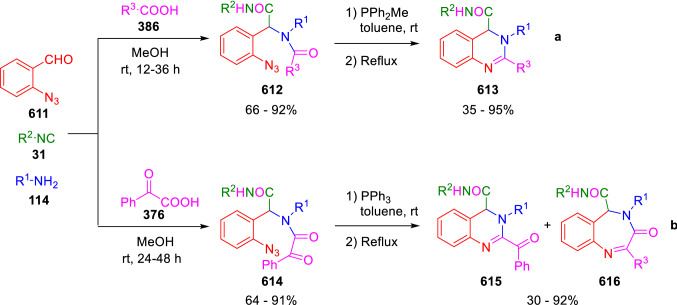
Ugi/Staudinger/aza-Wittig strategy employed by Ding

Ding et al. have used analogous Ugi/Staudinger/aza-Wittig sequences using 2-azidobenzaldehyde (**611**) for the synthesis of different heterocycles. The reaction of 2-azidobenzaldehyde (**611**), amines (**114**), isocyanides (**31**), and carboxylic acids (**386**), followed by treatment with methyldiphenyl phosphine yields the expected quinazolines (**613**; Scheme 127, a) [199]. When phenylglyoxylic acid (**376**) is used**,** 2-acylquinazolines (**615**) and/or 3*H*-1,4-benzodiazepin-3-ones (**616**) are obtained, depending on the steric and electronic nature of amine substituents (Scheme 127, a) [200].

**Scheme 128:**
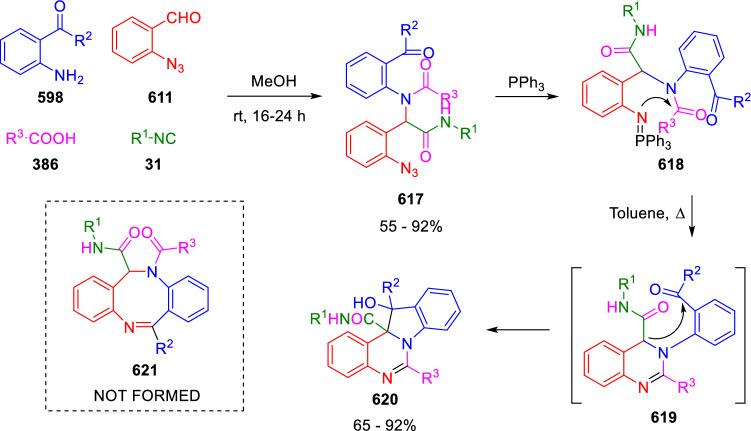
Synthesis of indolo[1,2-*c*]quinazolines

When the Ugi-adducts (**617**) of 2-azidobenzaldehyde (**611**) with 2-acylaniline (**598**), isocyanides (**31**), and carboxylic acids (**386**) were treated with triphenylphosphine, the expected benzodiazocines (**621**) were not formed. Instead, indolo[1,2-*c*]quinazolines (**620**) were obtained, probably through quinazoline intermediates (**619**), which upon heating in toluene were transformed in situ by nucleophilic addition of the peptidyl carbon on the carbonyl group (Scheme 128). The authors argue that this is probably due to a restricted conformation of the iminophosphorane (**618**) that would be entropically unfavourable for the cyclisation between the iminophosphorane moiety and the ketone carbonyl group [201].

**Scheme 129:**
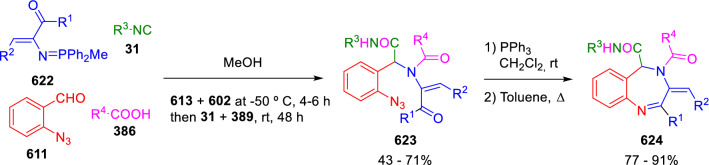
Synthesis of benzodiazepines developed by Ding

Ding has also used vinyliminophosphorane (**622**) as amine component in a U-4CC with 2-azidobenzaldehyde (**611**), isocyanides (**31**) and carboxylic acids (**386**). The resulting Ugi adducts (**623**) were treated with triphenylphosphine and afforded the 3-arylidene-substituted 3*H*-1,4-benzodiazepines (**624**) by the usual Staudinger/aza-Wittig sequence (Scheme 129) [202].

**Scheme 130:**
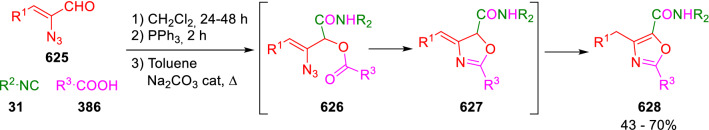
Tandem Passerini/Staudinger/aza-Wittig synthesis of oxazoles

Likewise, Ding carried out a tandem Passerini/Staudinger/aza-Wittig starting from α-azidocinnamaldehydes (**625**), to obtain 2,4,5-trisubstituted oxazoles (**628**) in a one-pot process. A catalytic amount of potassium carbonate is necessary to the complete transformation of dihydrooxazoles (**627**) into the final oxazoles (**628**) *vía* a 1,3-H shift (Scheme 130) [203]

**Scheme 131:**
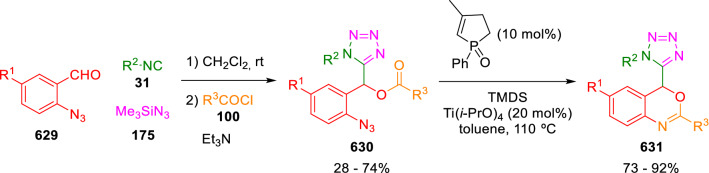
Passerini/acylation/Staudinger/catalytic aza-Wittig synthesis of benzoxazines

Similarly, the hydrazoic variant of the Passerini condensation of 2-azidobenzaldehydes (**629**) in tandem with an acylation/Staudinger/catalytic aza-Wittig reaction readily gave 4-tetrazolyl-substituted 4*H*-3,1-benzoxazines (**631**; Scheme 131) [204].

**Scheme 132:**
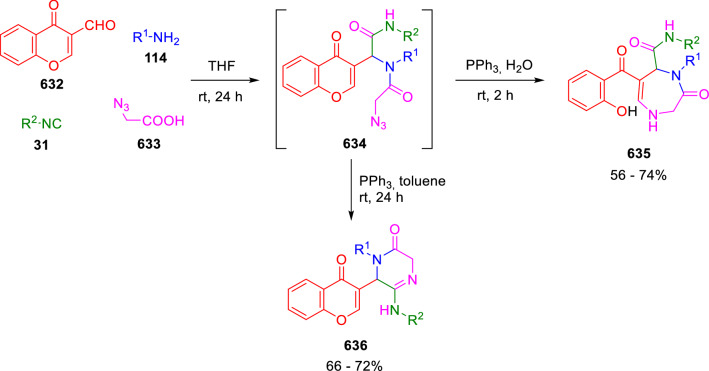
Synthesis of coumarinpyrazinones and oxazines reported by Bazgir

Bazgir used 2-azidoacetic acid (**633**) in an Ugi reaction with 3-formylchromones (**632**), isocyanides (**31**) and amines (**114**). The Ugi adduct (**634**) was not isolated and was treated in situ with triphenylphosphine. When the reaction was carried out in water, the intermediate iminophosphorane was hydrolysed and an intramolecular nucleophilic attack of the resulting amine, followed by a ring opening, took place to give the 3-oxo-1,4-diazepine-5-carboxamides (**635**). In turn, when dry toluene was used, the aza-Wittig reaction between the iminophosphorane and the amide group gave coumarinpyrazinones (**636**). The expected attack of the iminophosphorane on the chromone carbonyl group is probably not favoured due to the restricted conformation of intermediate (**634**) and it was not observed (Scheme 132) [205].

**Scheme 133:**
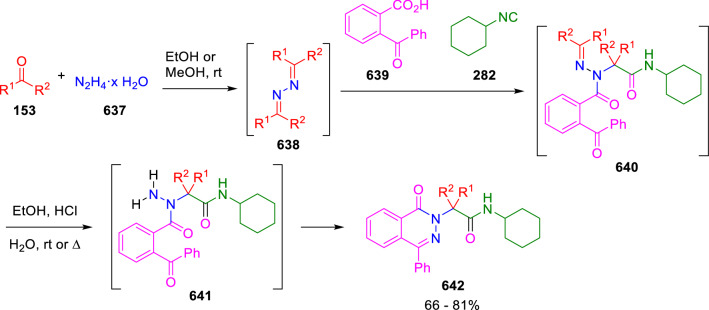
U-4CC with masked hidrazines

Marcaccini also used azines and semicarbazones as components for the Ugi condensation containing masked nucleophiles useful for post-condensation transformations. The reaction between hydrazine hydrate (**637**) and oxocompounds (**153**) affords the azine (**638**), which reacts with 2-benzoylbenzoic acid (**639**), and cyclohexyl isocyanide (**282**) to give the Ugi adduct (**640**). Acid hydrolysis of the hydrazone (**640**) liberates the hydrazine nucleophile and triggers an intramolecular cyclisation that results in 4-phenyl-1-(2*H*)phthalazinone-2-alkanoic acid amides (**642**; Scheme 133) [206].

**Scheme 134:**
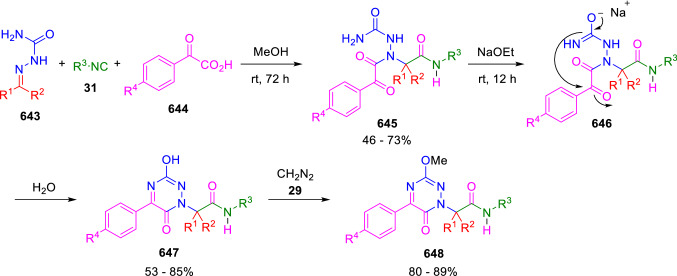
Use of semicarbazones in the Ugi reaction

Semicarbazones (**643**) were used for the first time in an Ugi reaction by Marcaccini for the synthesis of triazines (**648**). The reaction of semicarbazones (**643**) with isocyanides (**31**) and benzoyl-, or 4-methoxybenzoylformic acid (**644**) gave the expected Ugi adducts (**645**), which by treatment with sodium ethoxide resulted in a nucleophilic attack of the amide to the glyoxylic acid moiety leading to [1, 2, 4]triazines (**647**). Treatment of triazines **647** with diazomethane (**29**) led to the formation of the corresponding *O*-methyl derivatives (**648**; Scheme 134) [207]. Interestingly, compounds **647** and **648** constitute a new class of conformationally constrained pseudopeptides.

### Carbon nucleophiles

Carbon C–H centres contiguous to electron-withdrawing groups can act as internal nucleophiles in the presence of an appropriate base or, sometimes, spontaneously. This strategy has been extensively used by Marcaccini and other researchers to facilitate post-condensation reactions of Ugi and Passerini adducts.

**Scheme 135:**
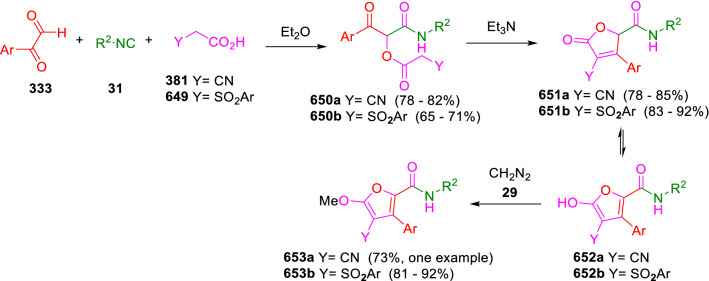
Synthesis of tetrasubstituted furans

Thus, Bossio and Marcaccini described the preparation of tetrasubstituted furans (**653**) by a Passerini reaction of arylglyoxals (**333**)**,** isocyanides (**31**)**,** and cyanoacetic (**381**) [208] or arenesulfonylacetic acids (**649**) [209] followed by subsequent treatment with base and diazomethane (**29**). In this process, the Passerini adducts (**650**) were formed and isolated, and then are subjected to a base triggered intramolecular Knoevenagel condensation between the oxo group and the activated methylene to give the furan derivatives (**651–652**). Methylated derivatives (**653**) were obtained by treatment of compounds **652** with diazomethane (Scheme 135).

**Scheme 136:**
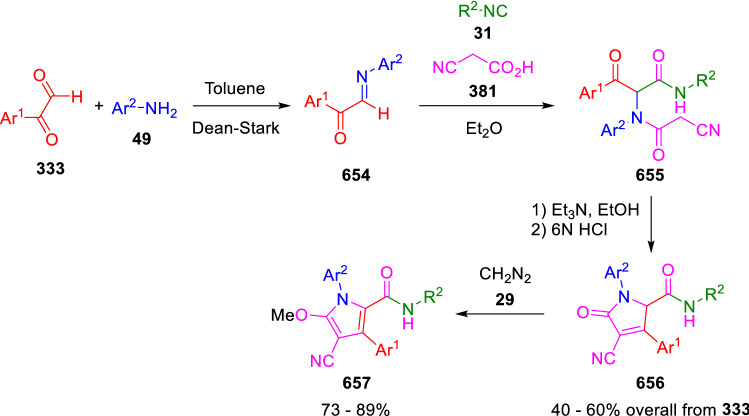
Synthesis of tetrasubstituted pyrroles

In order to extend this methodology to the preparation of pyrrole derivatives (**657**), these authors used arylglyoxal anils (**654**) in place of arylglyoxals (**333**). Arylglyoxal anils (**654**) were prepared from arylglyoxals (**333**) and anilines (**49**) and subjected to an Ugi condensation with cyanoacetic acid (**381**) and isocyanides (**31**). Cyclisation of the adducts (**655**) and methylation readily led to the desired pyrroles (**657**; Scheme 136) [124].

**Scheme 137:**
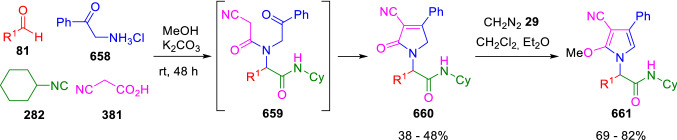
Synthesis of *N*-cyclohexyl-2-aryl-2-(3-cyano-2-methoxy-4-phenylpyrrol-l-yl)acetamides

Analogously, the Ugi reaction of aldehydes (**81**), phenacylamine hydrochloride (**558**), cyanoacetic acid (**381**), and cyclohexyl isocyanide (**282**) affords the intermediate Ugi adducts (**659**), which cyclise spontaneously to give pyrrolinones (**660**). Subsequent treatment with diazomethane (**29**) yields methylated pyrroles (**661**; Scheme 137) [210].

**Scheme 138:**
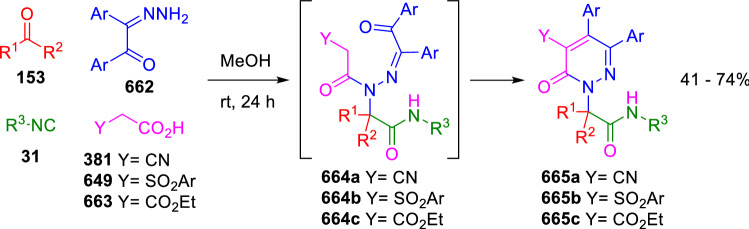
One-pot synthesis of pyridazinones

More recently, Marcaccini, Torroba and Marcos performed U-4CC employing diarylglyoxal monohydrazones (**662**) as the amino components, isocyanides (**31**), aldehydes or ketones (**153**), and carboxylic acids bearing an activated methylene group, such as cyanoacetic acid (**381**), tosylacetic acid (**649**) and malonic acid monoethyl ester (**663**). The resulting Ugi products (**664a-c**) spontaneously cyclised via an intramolecular Knoevenagel condensation to 3(2*H*)-pyridazinones (**665a-c**; Scheme 138) [125].

**Scheme 139:**
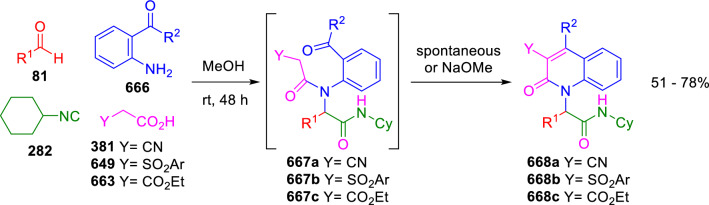
Ugi/Knoevenagel strategy for the synthesis of quinolinones

The tandem Ugi–Knoevenagel strategy was also applied to the synthesis of quinolin-2-(1*H*)-ones (**668**), starting from 2-aminophenylketones (**666**), aldehydes (**81**), cyclohexylisocyanide (**282**) and malonic or arylsulfonylacetic acid derivatives (**381**, **649**, **663**; Scheme 139) [126].

**Scheme 140:**
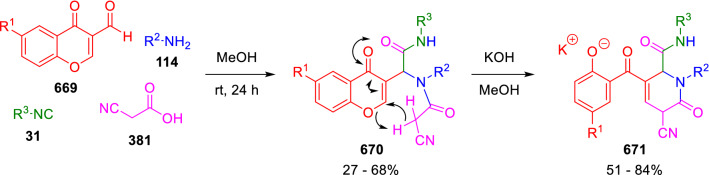
Synthesis of milrinone analogues

In continuation with these post-Ugi transformations, Marcos et al. synthesised the Ugi adducts (**670**) of 3-formylchromones (**669**) with cyanoacetic acid (**381**), amines (**114**) and isocyanides (**31**). Treatment of these adducts (**670**) with KOH in methanol led to a ring-opening/ring-closing process that produced efficiently polyfunctionalised pyridones (**671**) related to cardiotonic agent milrinone (Scheme 140) [127].

**Scheme 141:**
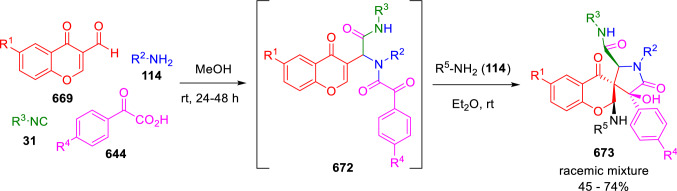
Diastereoselective synthesis of spiropyrrolidinochromanones

Going one step further, they developed a one-pot, two-step diastereoselective synthesis of spiropyrrolidinochromanones (**673**). The Ugi reaction between 3-formylchromones (**669**), amines (**114**), isocyanides (**31**), and glyoxylic acids (**644**) affords the Ugi adducts (**672**) that suffer a nucleophilic conjugate addition to the position 2 of the chromone ring of a second amine (**114**) and subsequent intramolecular cyclisation to give the spiranic structures (**673**) in diastereoselective manner. Remarkably, three new stereogenic centers are formed in the cyclisation process (Scheme 141). Ugi adducts are stable and can be isolated, *but *their purification is not necessary and can be directly used in the following step [211].

**Scheme 142:**
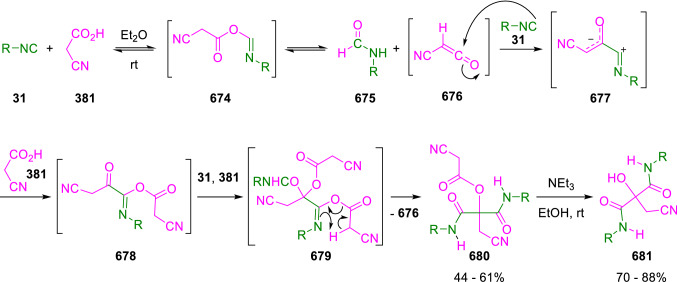
Reaction of isocyanides with 2-cyanoacetic acid

On the other hand, Marcaccini et al. also reacted isocyanides (**31**) with cyanoacetic acid (**381**) in the absence of carbonyl compounds affording *N*,*N*′-disubstituted 2-cyanoacetoxy-2-cyanomethylmalondiamides (**681**). According to the mechanism proposed by the authors, the key step is the formation of intermediate ketene (**676**) by deamidation of the adduct of cyanoacetic acid and isocyanide (**674**). Cyanoketene (**676**) is then attacked by a second molecule of isocyanide (**31**) and cyanoacetic acid (**381**) to form the intermediate (**678**). The carbonyl group in (**678**) undergoes a regular Passerini reaction and eliminates a further molecule of cyanoketene (**676**), ensuring the cyclic continuation of the reaction. It is also possible that cyanoketene elimination takes place before the final Passerini condensation (Scheme 142) [212].

### Nucleophilic groups introduced with external reagents

**Scheme 143:**
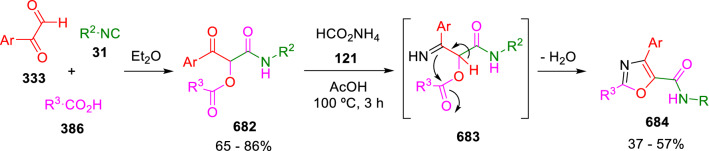
Synthesis of oxazole-5-carboxamides developed by Marcaccini

The addition of, usually bifunctional, nucleophiles to Passerini and Ugi adducts containing complementary electrophilic sites can lead to the formation of novel heterocyclic structures in a very efficient manner. With this aim Marcaccini developed different post-condensation transformations using ammonia or bisamine equivalents. For example, he treated arylglyoxals (**333**) with carboxylic acids (**386**) and isocyanides (**31**) to give *N*-alkyl-2-acyloxy-3-aryl-3-oxopropionamides (**682**). Upon heating of this Passerini adduct (**682**) with ammonium formate (**121**) in acetic acid, 5-oxazolecarboxamides (**684**) were obtained in fair yields (Scheme 143) [213, 214]. This strategy takes advantage of the reactivity of the peptidyl carbon on intermediate (**683**) and is in that way similar and complementary to the synthesis of oxazoles from Ugi adducts shown in Scheme 90. Interestingly, when arylthioacetic acids (**386**, R^3^ = CH_2_SAr) were used, sulfur-substituted oxazole-5-carboxamides (**684**) were obtained [215].

**Scheme 144:**
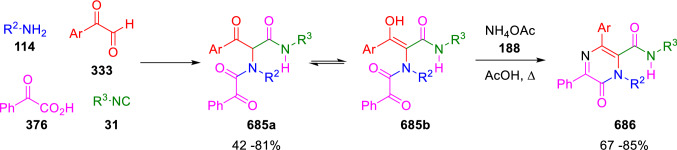
Synthesis of phenylpyrazine-2-carboxamides

Taking this idea further, the Ugi reaction between arylglyoxals (**333**), amines (**114**), benzoylformic acid (**376**) and isocyanides (**31**) afforded the Ugi adducts (**685**). The four-component reaction was carried out in two different manners: preforming the corresponding imines (**654**) from amines (**114**) and arylglyoxals (**333**) or following the classical one-pot procedure. The two methods led to the expected product with similar yields as an equilibrium mixture of tautomers (**685a** and **685b**). Treatment of these Ugi adducts (**685**) with ammonium acetate (**188**) afforded 1,*N*-disubstituted 4-aryl-1,6-dihydro-6-oxo-5-phenylpyrazine-2-carboxamides (**686**) by a Davidson type cyclisation (Scheme 144) [216].

**Scheme 145:**
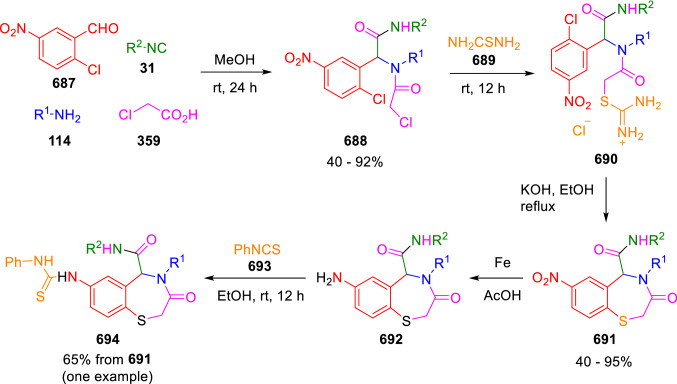
Synthesis of 4,5-dihydro-1,4-benzothiazepin-3(2*H*)-ones

Marcaccini also described the synthesis of benzothiazepinones (**691**) from 2-chloro-5-nitrobenzaldehyde (**687**), amines (**114**), isocyanides (**31**), and chloroacetic acid (**359**). Treatment of the Ugi adduct (**688**) with thiourea (**689**) resulted in the nucleophilic substitution of the aliphatic chlorine atom, followed by an aromatic nucleophilic substitution to give the benzothiazepinones (**691**). Then, the nitro-group could be reduced with iron in acetic acid to give an amine (**692**) that reacted with phenyl isothiocyanate (**693**) resulting in thiourea (**694**) (Scheme 145) [217].

## Adduct cleavage

**Scheme 146:**
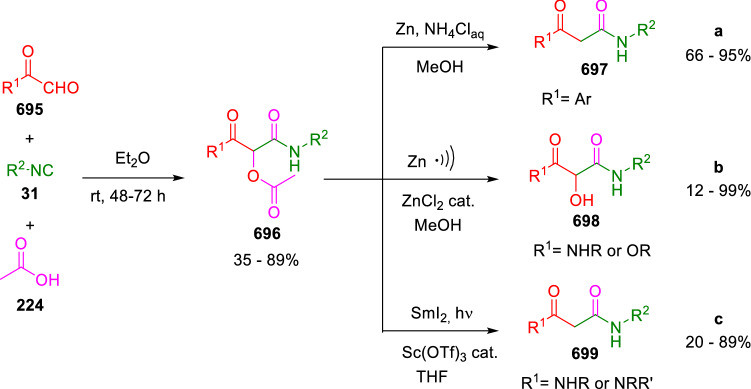
Post-Passerini transformations leading to 1,3-dicarbonylic compounds

Cleavage reactions of the IMCR adducts can lead to compounds difficult to obtain by other procedures. An example of this strategy used by Marcaccini for the synthesis of glycine derivatives (**543**) is shown in Scheme 113. Using a different approach, Marcaccini and Marcos synthesised different 1,3-dicarbonilic compounds in two steps by a Passerini/hydrolysis or a Passerini/reduction strategy. The reaction between glyoxals (**695**), isocyanides (**31**) and acetic acid (**224**) gave the expected Passerini adducts (**696**). When these α-acyloxyamides (**696**) were treated with activated zinc in aqueous methanol, the corresponding *β*-keto amides (**697**) were obtained (Scheme 146, a) [218]. When glyoxylamides or glyoxylesters (**695**, R^1^ = NHR or OR) were used in the Passerini condensation, tartronodiamides (**698**, R^1^ = NHR) and tartronoamidoesters (**698**, R^1^ = OR) were obtained by zinc catalysed solvolysis of the corresponding Passerini adducts (**696**; Scheme 146, b), [219]. Finally, when the Passerini adducts of glyoxylamides (**696**, R^1^ = NHR or NRR’) were reduced with samarium diiodide, malonodiamides (**699**) were obtained (Scheme 146, c) [220]. This protocol permits, in a simple and efficient manner, the synthesis of malonic subunits that can be useful in the synthesis of retro-peptides.

## Ugi reactions with phenols

**Scheme 147:**
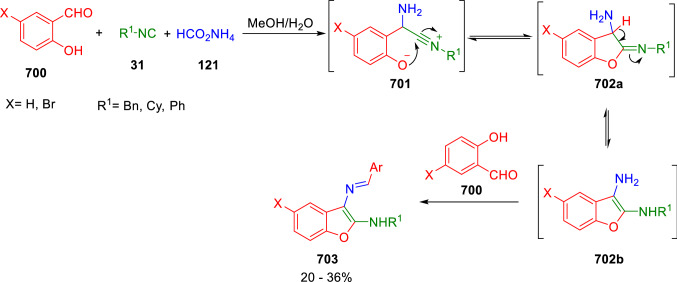
Synthesis of benzofurans by an intramolecular phenol-Ugi reaction

Marcaccini pioneered the use of phenols as substitutes for carboxylic acid in Ugi reactions [183]. Reaction of salicylaldehyde (**700**) with ammonium formate (**121**) and isocyanides (**31**) readily gave benzofuran derivatives (**703**; Scheme 147). In this case, the primary adduct (**702a**) is obtained through an intramolecular attack of the phenoxide on the nitrile cation in the reaction intermediate (**701**). The tautomerisation of this primary adduct produces diaminofuran (**702b**), which reacts with a second molecule of salicylaldehyde (**700**) giving benzofuran (**703**).

**Scheme 148:**
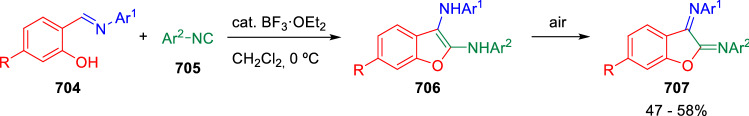
Kobayashi’s synthesis of diaminobenzofurans based on Marcaccini’s chemistry

Several years later, Kobayashi reported the preparation of 2,3-bis(arylamino)benzofurans (**706**) and/or 2,3-diimino-2,3-dihydrobenzofurans (**707**) based on the reaction of 2-aryliminophenols (**704**) and aryl isocyanides (**705**) with boron trifluoride catalysis (Scheme 148) [221]. A similar approach, using a secondary amine, electron-poor salicylaldehydes and aliphatic or aromatic isocyanides in the presence of catalytic SiO_2_ was reported by Ramazani [222]. In this latter case, the presence of a tertiary amine on benzofuran position 3 does not allow further oxidation to the corresponding diimine derivative and the 2,3-diaminobenzofurans are isolated as the only products. Very similar synthetic strategies were later published by Chattopadhyay’s [223] and Adib’s [224] groups, using respectively cerium ammonium nitrate as Lewis acid catalyst and water as solvent.

**Scheme 149:**
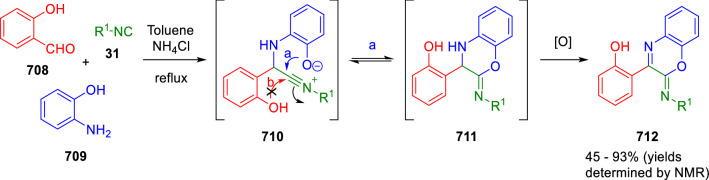
González-Zamora approach for the synthesis of 2-imino-1,4-benzoxazines

A related application, in which two phenol groups compete as acid components in an intramolecular reaction, was developed by González-Zamora et al. for the synthesis of 2-imino-1,4-benzoxazines (**712**; Scheme 149) [225].

**Scheme 150:**
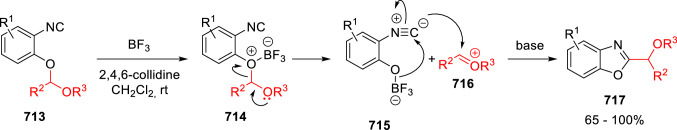
Wada’s synthesis of benzoxazoles

Wada et al. published a synthesis of 2-(alkoxyalkyl)-benzoxazoles (**717**) by a tandem migration/carboalkoxylation of *o*-isocyanophenyl acetals (**713**) catalysed by BF_3_·OEt_2_ and 2,4,6-collidine. The authors propose two alternative mechanisms for this transformation, one of which resembles a Passerini-type reaction with an ethylideneoxonium intermediate (**716**) and the phenol group acting as acid component (Scheme 150) [226].

**Scheme 151:**
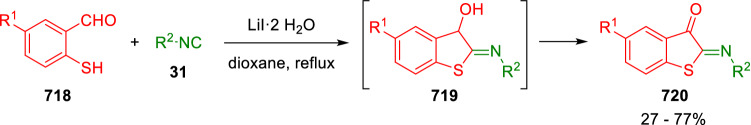
Ukaji’s synthesis of iminobenzothiophenones

The reaction of thiophenols (**718**) as acid components in intramolecular Passerini-type reactions has also been recently reported by Ukaji et al. (Scheme 151) [227]. The reaction is promoted by LiI, and in situ oxidation of the Passerini adduct (**719**) leads to benzothiophene-3(2*H*)-one (**720**).

**Scheme 152:**
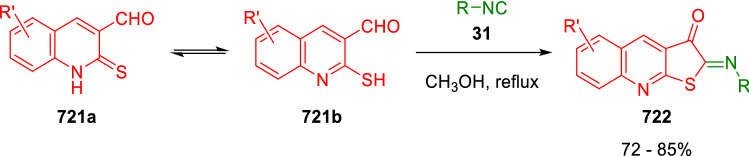
Shiri’s synthesis of iminothiophenone-fused quinolines

Shiri et al. performed a similar reaction starting from 2-mercaptoquinoline-3-carbaldehyde (**721**), resulting in tricyclic 2-(cyclohexylimino)thieno[2,3-*b*]quinolin-3(2*H*)-ones (**722**) through an intramolecular Passerini/oxidation tandem process (Scheme 152) [228].

**Scheme 153:**
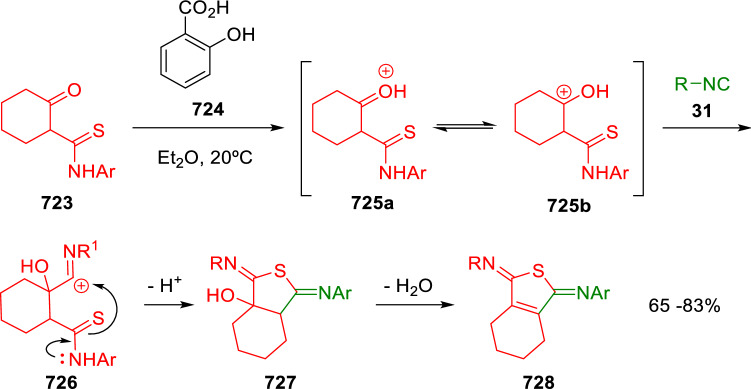
Formal [4 + 1] cycloaddition 3-oxothioamides and isocyanides

An interesting related precedent had been published by Marcaccini 20 years earlier. Thus, Marcaccini et al. allowed 2-(arylaminothiocarbonyl)cyclohexanones (**723**) and isocyanides (**31**) to react in an acidic medium to give novel diimino thioanhydrides (**728**; Scheme 153). Although the reaction can be considered as a formal [4 + 1] cycloaddition, the authors propose a two-step mechanism involving an initial protonation of carbonyl group, followed by the nucleophilic attack of the carbenoid carbon of the isocyanide and nucleophilic attack of the thioamide sulfur on the intermediate imidoyl cation (**726**). Final elimination of water takes place easily because of the highly conjugated nature of the final products (**728**). Salicylic acid (**724**) was found to provide a suitable acid catalysis to facilitate this process [229].

**Scheme 154:**
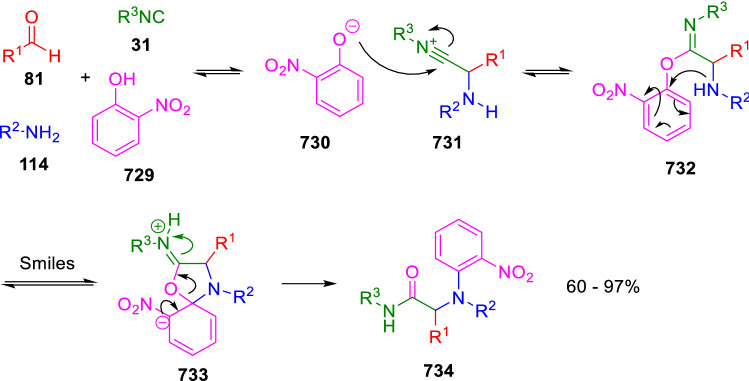
Ugi-Smiles reaction developed by El Kaïm

Later El Kaïm developed a general-purpose strategy in which a condensation of Ugi with phenols (**729**) is followed by a Smiles rearrangement, resulting in four-component adducts (**734**) in good yields (Scheme 154) [230, 231]. The reaction takes place with electron-deficient phenols, typically ortho or para-nitrophenols (**729**). The primary adduct (**732**) undergoes a Smiles rearrangement to give rise to nitrophenyl aminoamides (**734**).

This strategy certainly increases the diversity of structures obtainable in Ugi-type reactions, although it is limited to the use of a relatively small number of phenols containing electron-withdrawing substituents.

## Enol-Ugi reactions

**Scheme 155:**
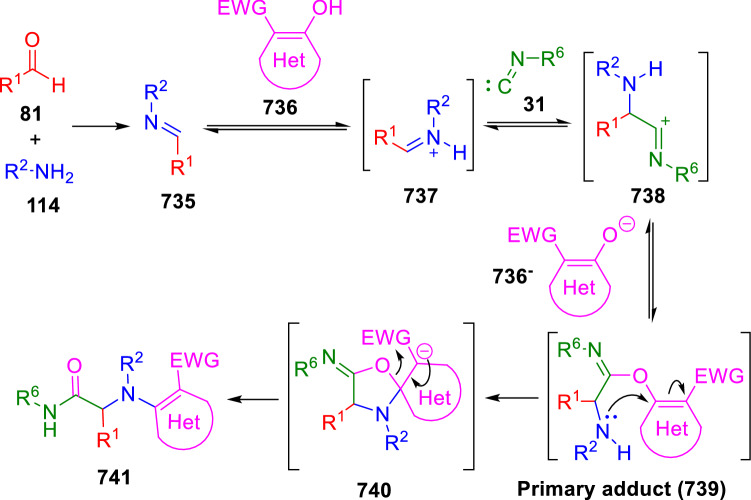
Mechanism of the enol-Ugi reaction

**Fig. 3:**
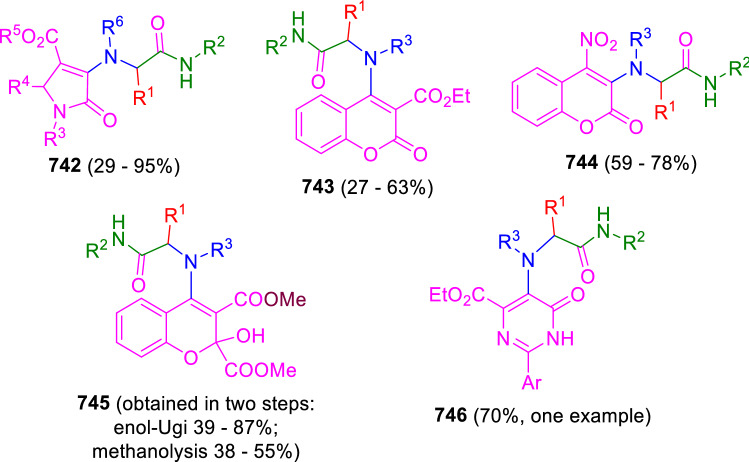
Heterocyclic enamines obtained by the enol-Ugi reaction

Enols have pK_a_ values comparable to that of phenols, while enolates are more nucleophilic than carboxylates. In 2012 Marcaccini and Marcos showed that heterocyclic enols (**736**) are feasible acid components in Ugi-type condensations [232]. The presence of an *α,β*-unsaturated electron-withdrawing group in the enol is a structural determinant that facilitates a Michael-retro-Michael rearrangement of the primary adduct (**739**; Scheme 155). This constitutes the driving force of the reaction and affords the heterocyclic enamine products (**741**). Different 5- and 6-membered enolic heterocycles can be used, including pyrrolidine-2,3-diones, furane-2,3-diones, 3- and 4-hydroxycoumarins and dihydropyrimidines, resulting in different heterocyclic enamines (**742**–**746**; Fig. 3) [232–234].

**Scheme 156:**
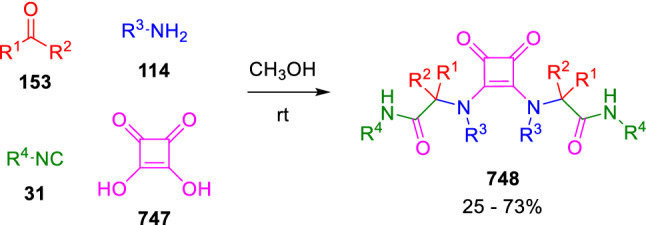
Charton’s synthesis of symmetrical squaramides

Independently and almost simultaneously, Julie Charton’s group published a related double 4-component Ugi-type reaction using squaric acid (**747**) as the acid component (Scheme 156) [235]. This reaction is also possible when substituting squaramic or squaramide acids for squaric acid, as it was later demonstrated by Mehrabi [236].

**Scheme 157:**
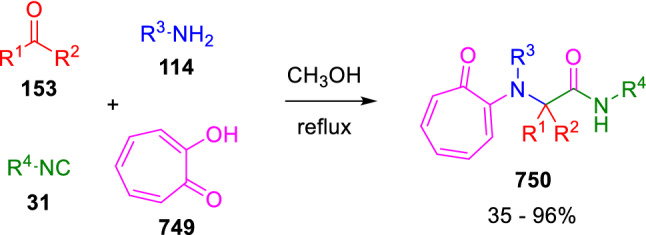
Ramazani’s enol-Ugi synthesis of tropolone-derived enamines

On the other hand, Ramazani et al. successfully used tropolone (**749**) as the enol component in enol-Ugi reactions leading to 2-(*N*,*N*-dialkylamino)-2,4,6-cycloheptatrien-1-one derivatives (**750**; Scheme 157) [237].

**Scheme 158:**
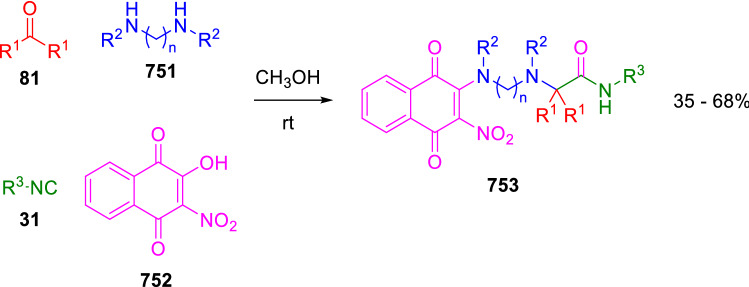
Rabêlo and Echemendía’s synthesis of substituted naphthoquinones

More recently, Rabêlo and Echemendía described an enol-Ugi reaction between 2-hydroxy-3-nitro-1,4 naphthoquinone (**752**), different secondary diamines (**751**) and isocyanides (**31**) to give 3-substituted 1,4 naphthoquinones (**753**; Scheme 158) [238].

**Scheme 159:**
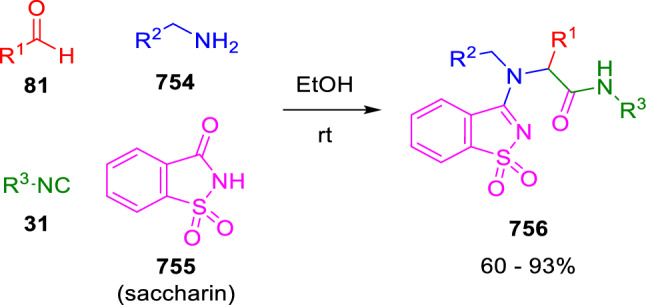
Ugi-type condensation with saccharin

In addition, Ramezanpour et al. reported a related variant of the Ugi condensation using saccharin (**755**) as acid surrogate (Scheme 159) [239].

**Scheme 160:**
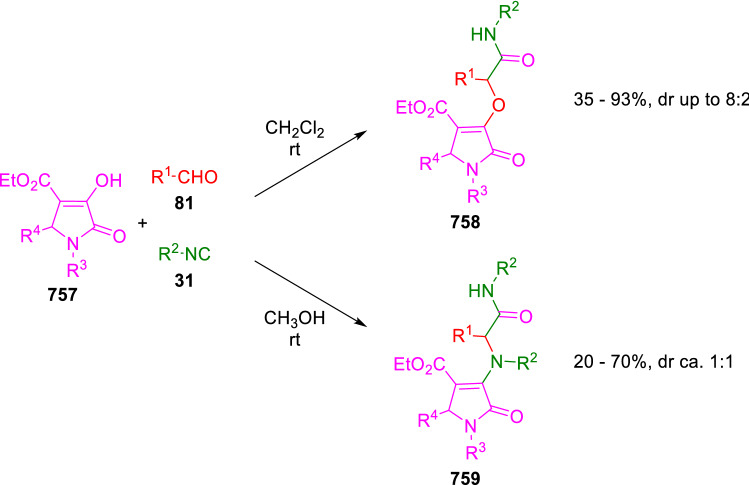
Enol-Passerini and pseudo-enol-Passerini reactions

In line with previous research, Marcos et al. developed analogous enol-Passerini and pseudo-enol-Ugi reactions from pyrrolidinodiones (**757**), isocyanides (**31**) and aldehydes (**81**; Scheme 160) [240].

**Scheme 161:**
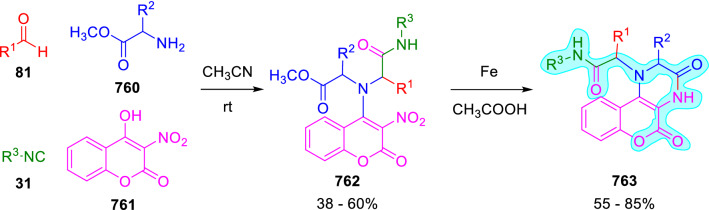
Chromenopyrazinone synthesis by an enol-Ugi post-condensation transformation

On the other hand, the post-condensation transformation of hydroxycoumarin enol-Ugi adducts (**762**) led to an efficient synthesis of geometrically restricted peptidomimetic chromeno[3,4-*b*]pyrazin-5-ones (**763**; Scheme 161) [241].

**Scheme 162:**
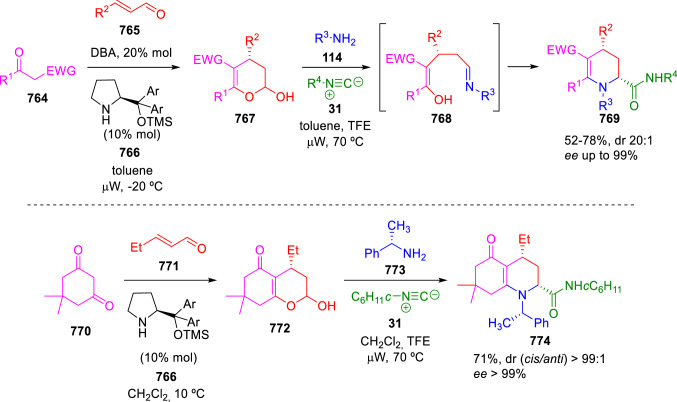
Intramolecular enol-Ugi condensation by Paixão and Rivera

Paixão et al. have developed an elegant stereoselective synthesis of tetrahydropyridines (**769**) [242] and related natural product hybrids (**774**) based on an intramolecular enol-Ugi condensation (Scheme 162) [243]. Interestingly, the reaction of hemiacetal (**767** or **772**) and an amine (**114** or **773**) generates a reactive intermediate (**768**) containing the enol and Shiff base moieties that make possible the ensuing enol-Ugi condensation.

## Cycloaddition reactions of isocyanides

### [3 + 2] Cycloadditions of bifunctional isocyanides

Isocyanoacetate derivatives (**1**) and other bifunctional isocyanides have been used by Marcaccini for the synthesis of heterocycles by α-addition to the isocyanide carbon and subsequent cyclisation on the acidic α position, as described earlier in this review. These bifunctional isocyanides can also be used in formal [3 + 2] cycloaddition reactions with dipolarophiles to afford 5-member nitrogen heterocycles.

**Scheme 163:**
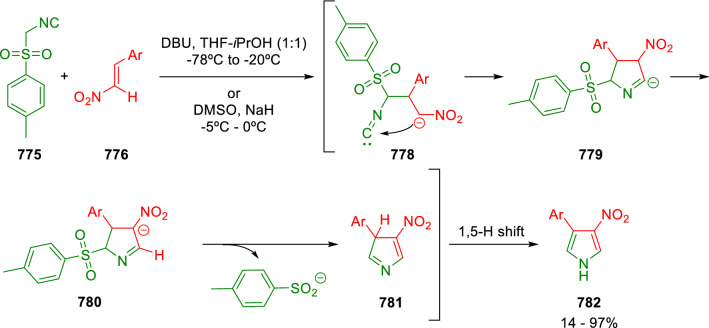
Barton’s synthesis of 3-nitropyrroles

The variation of the van Leusen reaction employing activated alkenes, such as *α,β*-unsaturated ketones, esters or nitriles, was reported in 1972 for pyrrole synthesis [244]. However, it was no until early 1990s, when Barton et al. made react aryl nitroolefins with TOSMIC in tetrahydrofuran-isopropanol, at – 78 °C, in the presence of DBU, to afford 3-nitropyrroles (Scheme 163) [245]. The formal [3 + 2] cycloaddition probably involves an asynchronous mechanism consisting in a first Michael addition of TOSMIC α carbon to the nitroolefin, followed by internal attack of the nitronate on the isocyano group. Proton exchange, elimination of the sulphone and aromatisation through a [1, 5] sigmatropic shift of hydrogen leads to the formation of a 3-nitropyrrole (**782**) in rather poor yields. The yields were though significantly improved by Ono et al., who carried out the reaction with NaH in DMSO [246].

**Scheme 164:**
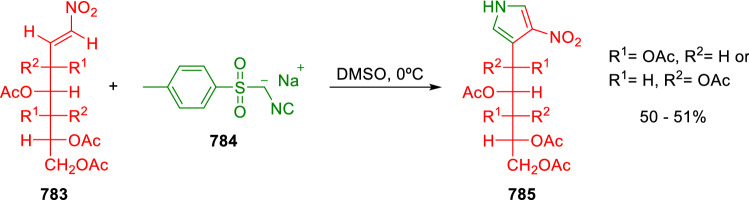
Synthesis of *C*-glycosil pyrroles

Marcaccini’s group applied this knowledge to the synthesis of sugar derivatives from TOSMIC sodium salt (**784**) and nitro-glucoside derivatives (**783**). In this way, nitropyrroles substituted with sugar functionalities were successfully obtained (Scheme 164) [247].

**Scheme 165:**
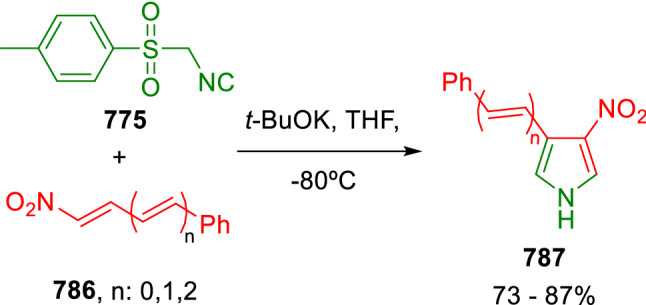
Van Leusen synthesis of 3-nitropyrroles

In a study contemporary to Marcaccini’s research, Van Leusen et al. also reported the synthesis 3-nitropyrroles from TOSMIC and nitrostyrenes, in almost quantitative yield and high-regioselectivities (Scheme 165) [248].

**Scheme 166:**
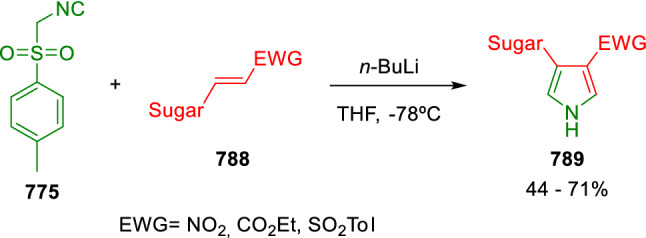
Synthesis of pyrrole *C*-Nucleosides

Later research by Krishna et al. expanded Marcaccini studies by reacting TOSMIC and activated alkene bonds to different ribose derivatives to obtain 3-nitropyrrole *C*-nucleosides (Scheme 166) [249, 250].

**Scheme 167:**
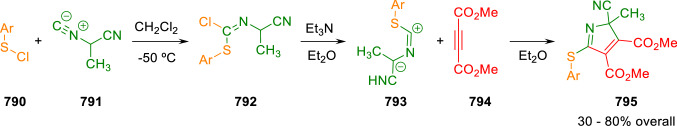
1,3-Dipolar cycloaddition of nitrile ylides derived from 2-isocyanopropionitrile

A different approach was developed by Marcaccini, based on the generation of a novel class of nitrile ylides by the treatment of isothiocarbamoyl chlorides (**792**) with triethylamine. These nitrile ylides react readily with dienophiles as acetylenedicarboxylate (**794**) through a 1,3-dipolar cycloaddition to give 2-*H*-pyrroles (**795**). The precursor isothiocarbamoyl chloride (**792**) was, in turn, easily prepared by the reaction of sulfenyl chlorides (**790**) and 2-isocyanopropionitrile (**791**; Scheme 167) [251].

**Scheme 168:**
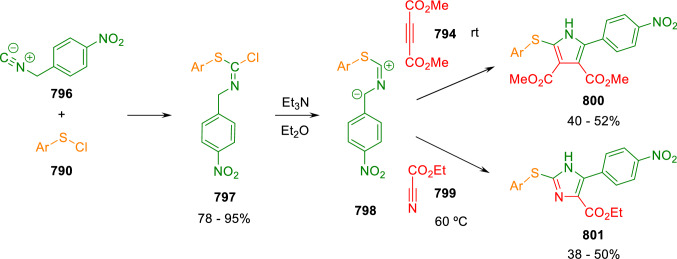
Synthesis of 1*H*-pyrroles and 1*H*-imidazoles via 1,3-dipolar cycloaddition

This strategy was extended to the cycloaddition of nitrile ylides (**798**) obtained from 4-nitrobenzoylisocyanides (**796**) and arenylsulfenyl chlorides (**790**). In this way, different 1-*H*-pyrroles (**800**) and 1-*H*-imidazoles (**801**) were readily prepared by the reaction with, respectively, dimethyl acetylenedicarboxylate (**794**) or ethyl cyanoformate (**799**; Scheme 168) [252].

### [4 + 1] Cycloadditions of isocyanides

The α-addition of 1,4-bifunctional reagents to isocyanides can result in 5-member heterocycles, product of a formal [4 + 1] cycloaddition process. Saegusa found that stoichiometric Et_2_AlCl promotes the [4 + 1] cycloaddition of isocyanides with *α,β*-unsaturated carbonylic compounds to afford unsaturated N-substituted iminolactones[253]. Later, this reaction was effectively carried out by Chatani in the presence of catalytic GaCl_3_ [254]. Yavari has similarly synthesised 2-aminofurans [255], although, several reports point out the instability of 2-aminofurans and their tendency to be quickly oxidized in the reaction medium [256]. On the other hand, Marcaccini and Marcos implemented different strategies to trap the elusive 2-aminofurans obtained by the [4 + 1] cycloaddition of isocyanides, resulting in tandem processes leading to diverse heterocyclic systems.

**Scheme 169:**
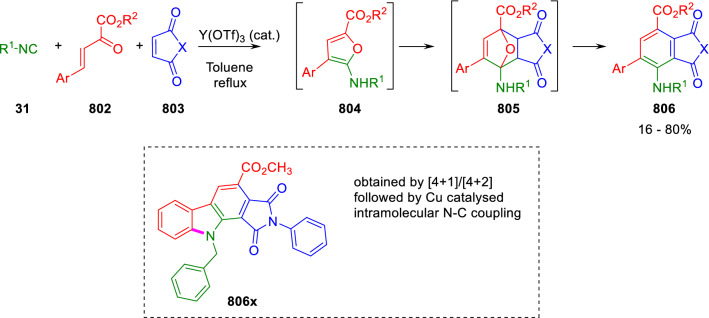
Synthesis of polysubstituted anilines by tandem [4 + 1]/[4 + 2] cycloaddition of isocyanides

Thus, an yttrium triflate catalysed tandem process involving the [4 + 1] cycloaddition of isocyanides (**31**) with *α,β*-unsaturated ketoester (**802**) and the ensuing [4 + 2] cycloaddition of the resulting unstable aminofuran (**804**) with maleimide derivatives (**803**) successfully gave polysubstituted anilines (**806**) in moderate to good yields (Scheme 169) [257]. The intermediate oxabycicle (**805**) was not isolated, but opening of the oxygen bridge and dehydratation take place inmediately in the reaction medium.

**Scheme 170:**
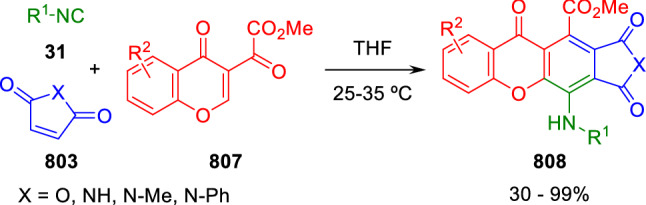
[4 + 1]/[4 + 2] Cycloaddition strategy for the synthesis of 4-aminoxanthones

Marcos et al. applied this methodology to a highly efficient synthesis of 4-aminoxanthones (**808**), starting from 3-carbonylchromones (**807**), isocyanides (**31**) and dienophiles (**803**; Scheme 170) [258]. The geometrical rigidity of the *α,β*-unsaturated carbonyl of the chromone drives the progress of the reaction, which proceeds without the need for catalysis at near room temperature.

**Scheme 171:**
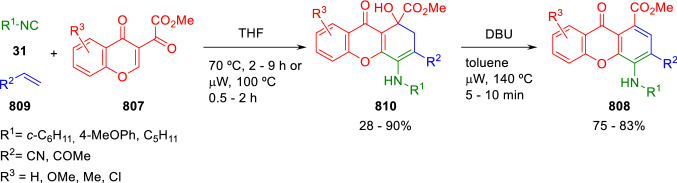
Synthesis of dixydroxanthones and xanthones

Interestingly, the use of non-symmetrical dienophiles, such as methylvinylketone or acrylonitrile (**809**), allows the isolation of the intermediate 1-hydroxydihydroxanthones (**810**), which in this case turn out to be stable products that can be further dehydrated in the presence of a base to give the corresponding xanthones (**808**; Scheme 171) [259, 260]. The reaction is fully regioselective so that the electron-withdrawing group R^2^ occupies the 3-position of the product xanthone or dihydroxanthone.

**Fig. 4:**
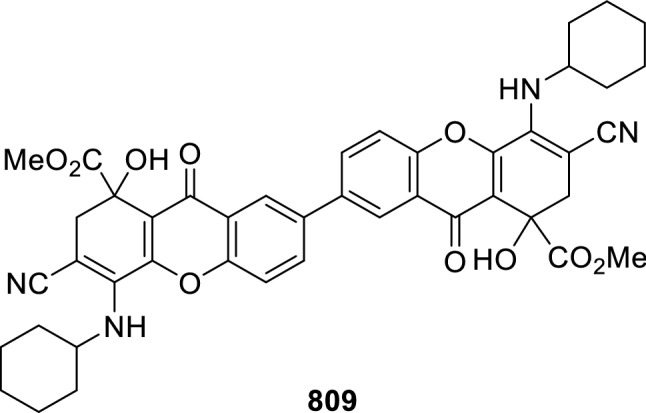
Secalonic acid analogue obtained by a tandem [4 + 1]/[4 + 2] cycloaddition of isocyanides

Xanthones related to natural products, such as secalonic acid analogue **809**, could be obtained using this simple procedure (Fig. 4).

A virtually identical synthesis of 4-aminoxanthones was later reported by Teimouri [261], who also used quinones as dienophiles to obtain 4 or 5 fused polycycles [262].

**Scheme 172:**
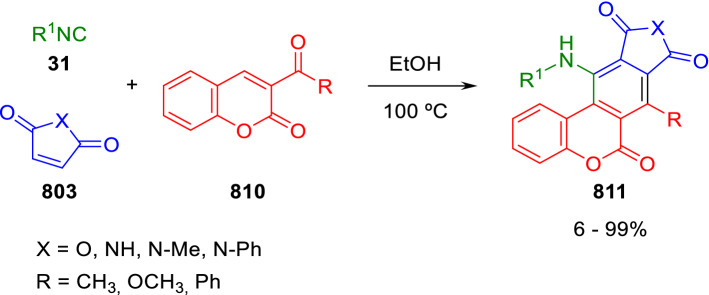
Tandem synthesis of benzocoumarins

Benzocoumarins (**811**) were also obtained by Marcos et al. by the tandem [4 + 1]/[4 + 2] cycloaddition of isocyanides (**31**), 3-carbonylcoumarins (**810**) and dienophiles (**803**; Scheme 172) [263].

**Scheme 173:**
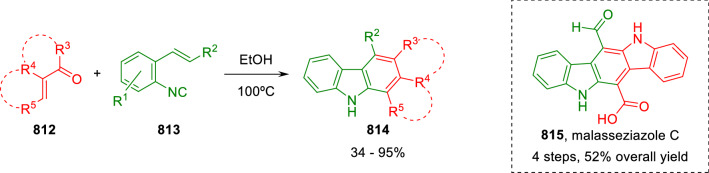
Tandem [4 + 1]/intramolecular [4 + 2] cycloaddition for the synthesis of carbazoles

Tang et al. have used *o*-alkenyl arylisocyanides (**813**), which react with *α*,*β*-unsaturated ketones (**812**) in a tandem [4 + 1]/intramolecular [4 + 2] process to afford carbazole and indolocarbazole alkaloid derivatives (**814**; Scheme 173) [264]. This strategy was recently used for the synthesis of the alkaloid malasseziazole C **815** [265].

**Scheme 174:**
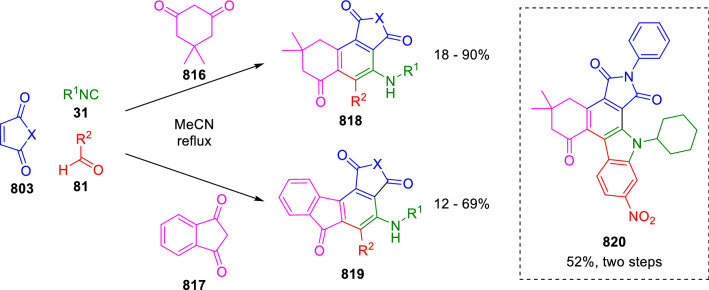
Tandem synthesis of polycyclic isoindoles

A different approach was also developed by Marcos’ group, where polycyclic isoindoles (**818** and **819**) were obtained in a tandem multicomponent synthesis starting from cyclic 1,3-dicarbonyls (**816** or **817**), aldehydes (**81**), isocyanides (**31**), and maleimides (**803**). The reaction involves a sequence of a Knoevenagel condensation, and [4 + 1] and Diels–Alder cycloadditions. Interestingly, a further microwave-promoted dehydrogenative N−C bond forming reaction allows the synthesis of a natural product-like isoindolocarbazoles (**820**, Scheme 174) [266].

**Scheme 175:**
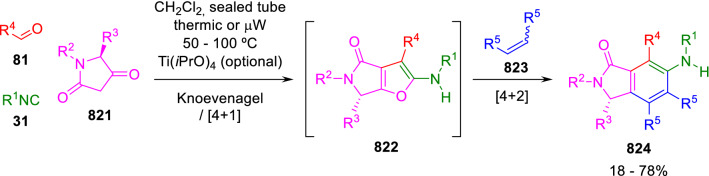
Byk’s synthesis of isoindoles

A related tandem Knoevenagel/double cycloaddition reaction was developed by Byk to obtain staurosporine analogues (**824**) [267]. This multicomponent reaction begins with the condensation of aldehyde (**81**) and substituted chiral tetramic acid (**821**), which under the reaction conditions suffer successive cycloadditions with an isocyanide (**31**) and a dienophile (**823**) to give the desired isoindoles (Scheme 175).

**Scheme 176:**
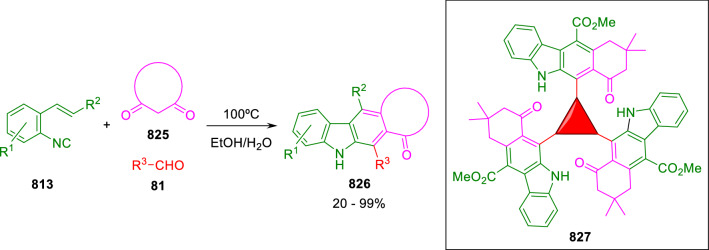
Tandem Knoevenagel/[4 + 1]/intramolecular [4 + 2] cycloaddition to cyclo[*b*]fused carbazoles

An intramolecular version of this type of process was again reported by the group of Tang in 2020, in which polyfunctionalised cyclo[*b*]fused carbazoles (**826**) are obtained in a catalyst-free aqueous media with yields up to 99% (Scheme 176) [268]. Di- and tricarbazoles (**827**) were obtained from di- and trialdehydes.

**Scheme 177:**
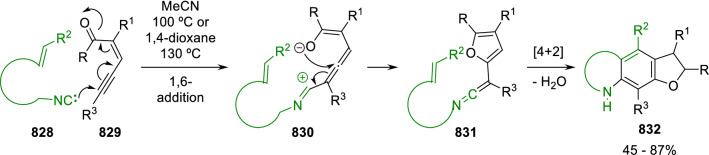
Tandem 1,6-addition/cyclisation/[4 + 2] cycloaddition

A related strategy, developed by Xu’s group consists in the formation of an intermediate furane (**831**) by the addition of alkenyl isocyanides (**828**) to ene-yne-ketones (**829**) and a subsequent intramolecular Diels–Alder cycloaddition (Scheme 177) [269].

**Scheme 178:**
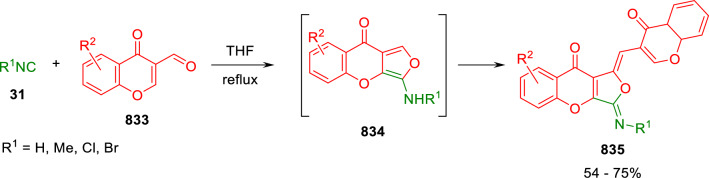
Tandem [4 + 1] cycloaddition/condensation for the synthesis of chromenylmethylene furochromones

In the absence of a dienophile, the [4 + 1] cycloaddition of formylchromones (**833**) and isocyanides (**31**) gives unstable aminofuranes (**834**) that can undergo a Friedel–Crafts-type addition to a second molecule of the aldehyde (**833**) and dehydrate to give chromenylmethylene furochromones (**835**; Scheme 178). Preliminary studies of this reaction were reported by the group of Marcos in 2008 [270, 271] and later published a complete study by spectroscopic and X-ray diffraction analyses in which the structure (**835**) was unequivocally confirmed [272]. Meanwhile, a report by Bandyopadhyay in 2010 [273] published the same reaction, but postulated a structure for the products that was later shown to be incorrect [274]. Teimouri also reported the same reaction with similar results [275].

**Scheme 179:**
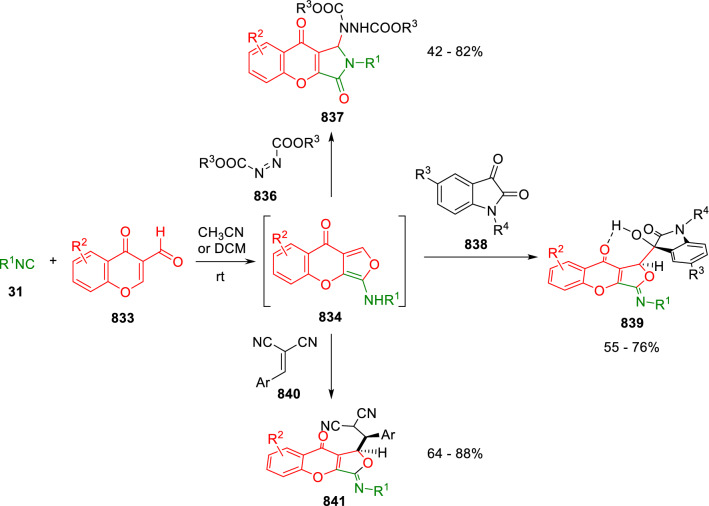
Tandem [4 + 1]/Friedel–Crafts reactions

The Friedel–Crafts trapping of 3-formylchromone derived aminofuranes with different electrophiles, such as azodicarboxilates (**836**) [276], isatines (**838**) [277] and arylidene malononitriles (**840**) [278], was later reported by Teimouri et al. (Scheme 179).

**Scheme 180:**
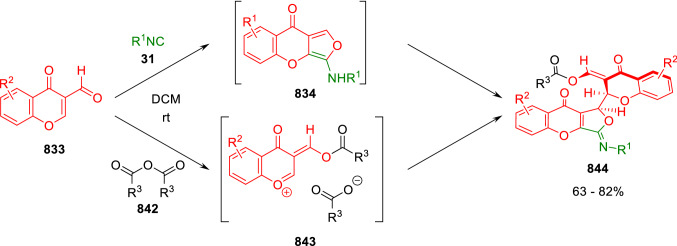
1,4-addition of intermediate aminofuranes to acylated formylchromones

They also performed the reaction of 3-formylchromones (**833**), isocyanides (**31**) and carboxylic acid anhydrides (**842**), which led to the formation of (acyloxymethylidene)chromonyl-furochromones (**844**) by the Friedel–Crafts attack of intermediate aminofurane (**834**) to an *O*-acylated chromone (**843**; Scheme 180) [279].

**Scheme 181:**
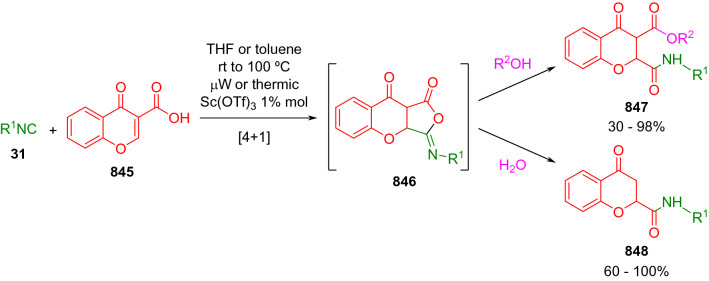
Conjugate addition of isocyanides to chromone 3-carboxylic acid

On the other hand, chromone 3-carboxylic acid (**845**), undergoes [4 + 1] cycloaddition with isocyanides (**31**) to give a highly reactive intermediate iminoanhydride (**846**), which in the presence of alcohol nucleophiles is opened to give chromone-2-carboxamido-3-esters (**847**; Scheme 181). If water is used as nucleophile, the resulting carboxylic acid decarboxylates spontaneously to give 2-amido-substituted chromanones (**848**) [280].

**Scheme 182:**
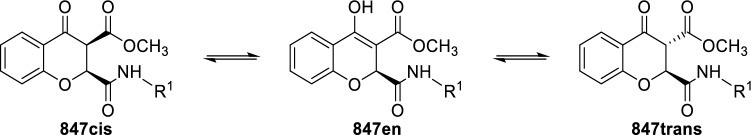
Equilibrium of isomers in chromone-2-carboxamido-3-esters

The product chromone-2-carboxamido-3-esters (**847**) are obtained as a mixture of the *cis* (**847cis**) and enol (**847en**) tautomers, which equilibrate in solution to a thermodynamic mixture of *cis* (**847cis**), *trans* (**847trans**) and enol (**847en**) isomers (Scheme 182).

**Scheme 183:**
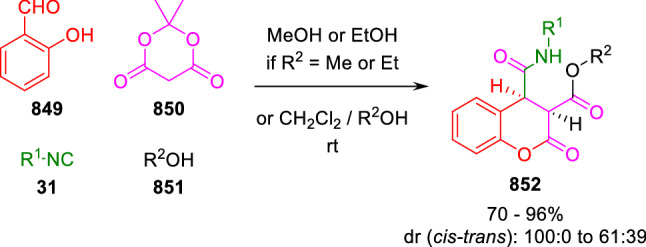
Four-component synthesis of 4-carbamoyl-2-oxochromane-3-carboxylates

A related-isocyanide four-component reaction was published by Shaabani in 2008 using Meldrum’s acid to obtain coumarin 4-carbamoyl-2-oxochromane-3-carboxylates (**852**) [281]. The mechanism involves the [4 + 1] cycloaddition of isocyanide (**31**) with the *α,β*-carbonyl intermediate, formed in situ through the condensation of aldehyde (**849**) and the Meldrum’s acid (**850**; Scheme 183). In this case, the major isomer was **852cis**, especially when bulky alcohols (**851**) and isocyanides (**31**) were used.

## Conclusions

Stefano Marcaccini is recognised as one of the foremost pioneers in isocyanide chemistry. The profound impact of his developments is clearly visible today in the work of those who follow his path and in the contributions of many first-rate research groups working in organic synthesis.

This review collects some of the overwhelming body of work developed by Stefano Marcaccini in the field of isocyanide chemistry. His encyclopaedic knowledge and his great creativity, as well as his scientific acumen, allowed him to utilise different strategies for the synthesis of heterocycles, some of which were still unpublished structures. Among other advancements, he made important contributions in the use of difunctional isocyanides, cycloaddition reactions of isocyanides, post-condensation transformations of adducts of classic multicomponent reactions, such as P3CC and U4CC, and development of new isocyanide multicomponent reactions using intramolecular methodologies and alternative reagents like phenols and enols.

With this review we want to gather the most important contributions of Stefano Marcaccini to organic synthesis and pay a fitting tribute to his memory.
